# Sensitized Disequilibration of Water‐Soluble Azopolymers

**DOI:** 10.1002/anie.202523447

**Published:** 2025-12-23

**Authors:** Henning Jörn Meteling, Julius Gemen, Satu Häkkinen, Rafal Klajn, Arri Priimagi

**Affiliations:** ^1^ Faculty of Engineering and Natural Sciences Tampere University P.O. Box 541 Tampere FI‐33101 Finland; ^2^ Organisch‐Chemisches Institut University of Münster Corrensstrasse 36 Münster 48149; ^3^ Institute of Science and Technology Austria (ISTA) Am Campus 1 Klosterneuburg 3400 Austria

**Keywords:** Azopolymers, Host‐guest complexes, Molecular photoswitches, Photosensitizers

## Abstract

Photo‐responsive systems based on azobenzenes usually require UV light for *E*→*Z* isomerization, limiting their applicability, especially in biomedical contexts. Disequilibration by sensitization of azobenzene under confinement (DESC) has recently emerged as a supramolecular strategy to bypass this limitation without the need to derivatize the azobenzene scaffold. Here, we expand DESC to water‐soluble azopolymers obtained by RAFT polymerization and systematically investigate the interplay between the polymer structure and DESC efficiency. Using this approach, we achieved as much as 85% of the direct photoexcitation (UV) switching efficiency, while utilizing low‐energy (yellow) light. These results establish general design principles for combining DESC with polymeric systems, opening new opportunities for the development of functional materials driven with low‐energy light.

## Graphical abstract

Disequilibration by sensitization under confinement (DESC) is a promising strategy for efficient visible‐light‐induced azobenzene *E*→*Z* isomerization. In this article, we explored key factors for adapting DESC to azopolymers, eventually achieving efficiencies up to 85% compared to direct excitation and laying the foundation for more complex systems.

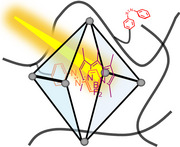

## Introduction

Functional materials, composed of synergistic (macro)molecular building blocks, can dynamically respond to environmental changes. Among the different external stimuli, light is particularly attractive due to its high spatiotemporal controllability and non‐invasive nature.^[^ ^1^, ^2^ ^]^ The light response is often based on isomerization of molecular photoswitches, harnessed to reversibly modulate the properties and function of systems they are incorporated into. This methodology has been exploited in various light‐driven phenomena such as light‐controlled catalysis^[^ ^3^, ^4^, ^5^ ^]^ but also in triggered self‐assembly and disassembly processes through pathways such as host‐guest complexation^[^ ^6^, ^7^, ^8^ ^]^ fibre formation^[^ ^9^, ^10^ ^]^ and alignment control of liquid crystals.^[^ ^11^, ^12^ ^]^

Arguably the most extensively studied and widely applied class of photoswitches is azobenzene and its derivatives, which undergo reversible *E*→*Z* photoisomerization.^[^ ^13^ ^]^ They are generally highly robust and photostable and can be incorporated into different kinds of materials. As a result, azobenzene‐based systems have been applied in various fields, including solar thermal energy storage technologies,^[^ ^14^, ^15^ ^]^ photobiology, and photopharmacology.^[^ ^16^, ^17^, ^20^, ^21^ ^]^ They are also widely utilized in soft materials, where they serve as powerful tools for controlling the properties of polymeric systems. In solution, azobenzene‐containing block‐copolymers exhibit photoinduced microscopic structural changes such as swelling or deformation of micellar assemblies.^[^ ^22^, ^23^, ^24^ ^]^

In bulk polymers, azobenzene photoisomerization enables the manipulation of macroscopic properties such as the glass transition temperature and hardness, or invokes phase transition, and has been exploited for reversible adhesion or surface pattern formation.^[^ ^25^, ^26^, ^27^, ^28^ ^]^ Isomerization of azobenzene can also alter self‐assembled structures, enabling actuation in liquid crystal networks^[^ ^29^, ^30^ ^]^ or stiffness changes and drug release in hydrogels.^[^ ^31^, ^32^, ^33^, ^34^, ^35^ ^]^ In all these examples, the performance or response of a given system depends on the isomerization characteristics of the photoswitch, and high conversion and long metastable‐state lifetimes are usually desired.

Traditionally, *E*→*Z* isomerization around the N═N double bond in azobenzene derivatives required UV light, which poses significant limitations in their applicability especially in biological contexts. To overcome this, considerable efforts have been made to red‐shift their absorption by changing their electronic and steric structure through different substitution patterns. Tetra‐*ortho*‐fluorinated and tetra‐*ortho*‐methoxylated azobenzenes marked milestones that have enabled visible‐light switching in both directions.^[^ ^36^, ^37^ ^]^ However, structural modifications inevitably alter the nature of the photoswitch, which may compromise performance for a given application. Hence it would be desirable to address azobenzene isomerization using visible light while preserving the intrinsic properties of the molecule in question.

An alternative approach to tailoring the photoswitch itself is to employ indirect isomerization. In this approach, energy transfer from a photosensitizer excited with visible or near‐infrared light to the azobenzene enables its isomerization without the need for irradiation with high‐energy photons.^[^ ^38^, ^39^, ^40^ ^]^ Until recently, this technique was only effective for triggering the relaxation from the metastable *Z* isomer to the thermodynamically stable *E* isomer. A significant advancement came with the introduction of a water‐soluble supramolecular cage capable of encapsulating otherwise insoluble *E*‐azobenzene derivatives along with a photosensitizer. This spatial confinement ensures the proximity necessary for the energy transfer to occur, thereby enabling near‐quantitative *E*→*Z* isomerization using visible light via “disequilibration of azobenzenes by visible light sensitization under confinement” (DESC).^[^ ^41^ ^]^ DESC opens new possibilities for creating water‐based light‐activated materials operated with low‐energy light, while retaining the qualities of the employed photoswitch. In a recent example demonstrating its versatility, DESC has been adopted in azoheteroarene‐based micellar systems to modulate surface tension using visible light.^[^ ^42^ ^]^

In this study, we extend the DESC concept into macromolecular systems by integrating it into linear, water‐soluble azopolymers based on *N,N*‐dimethylacrylamide. The polymers were synthesized in a two‐step protocol involving reversible addition‐fragmentation chain transfer (RAFT) polymerization followed by post‐modification to incorporate azobenzene pendants. We systematically investigate the effect of degree of polymerization (DP), functionalization density, and the substitution pattern of the incorporated azobenzene, on the DESC efficiency. Our findings reveal that a lower DP and, more significantly, lower functionalization density favor DESC. Furthermore, subtle modifications to the azobenzene structure, such as variations in the linker or terminal functional group enabled us to achieve up to 77 % *Z*‐isomer content using DESC. We anticipate that this study will lay the foundation for the development of water‐soluble light‐responsive macromolecular systems operating fully under visible light while retaining the desirable characteristics of the employed photoswitch.

## Results and Discussion

**Figure 1:**
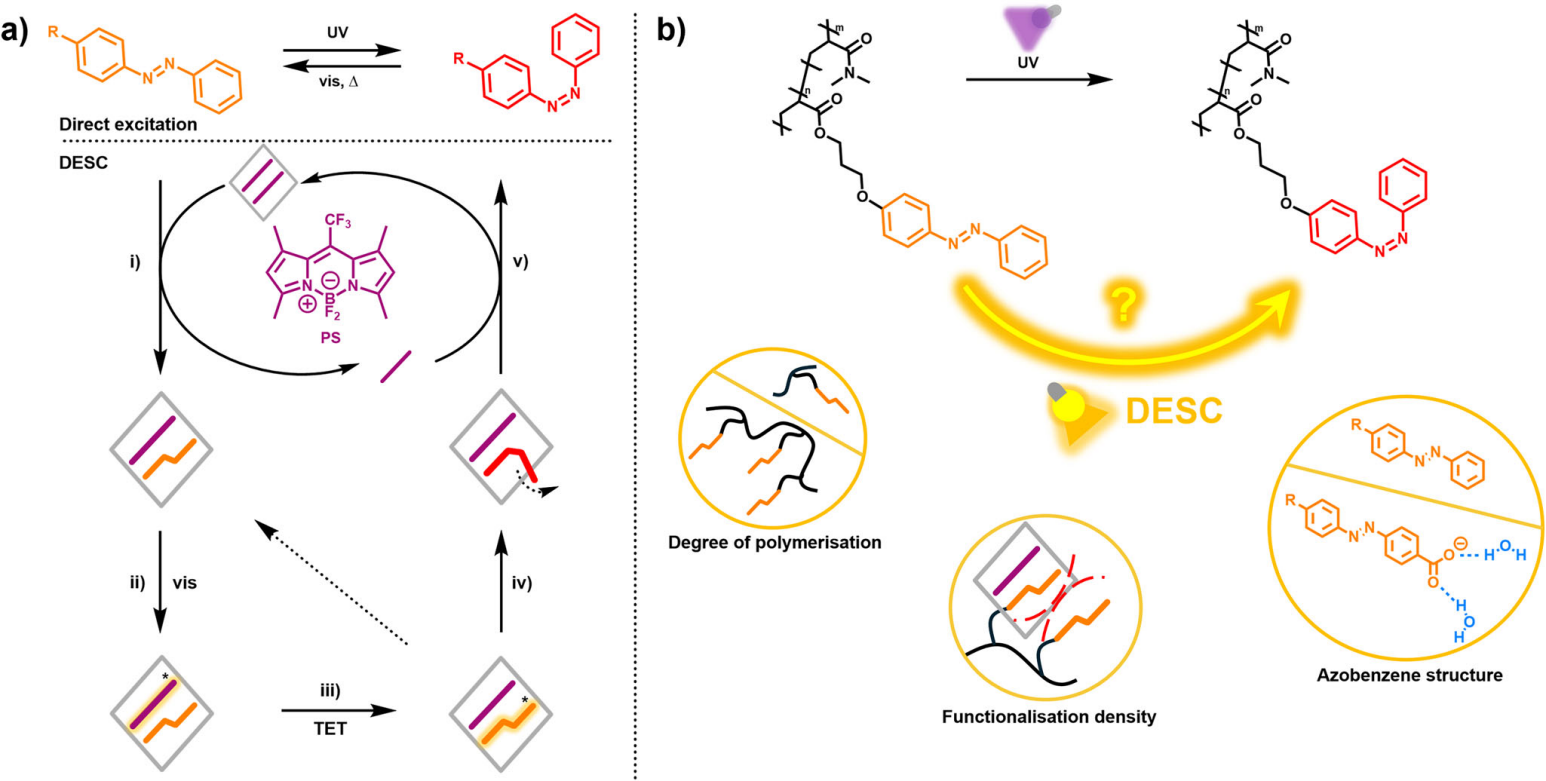
a) The working principle of DESC: i) heterodimer formation, ii) excitation of **PS**, iii) energy transfer to azobenzene, iv) *E*→*Z* isomerization, and v) expulsion from the host. b) Scheme for adapting DESC to polymer systems, tuning i) the degree of polymerization, ii) functionalization density, iii) azobenzene structure.

DESC in solution is executed by mixing the desired azobenzene (**Azo**) with a photosensitizer (**PS**) and a supramolecular host (**H**) capable of encapsulating two molecules of either guest. Rapid guest exchange can lead to the transient formation of **Azo**·**PS** heterodimers within the host (i.e., the (**Azo**·**PS**)⊂**H** ternary inclusion complex). By bringing **Azo** and **PS** into close proximity, host **H** enables efficient triplet energy transfer from the excited **PS** to **Azo** upon irradiation with yellow light (in our chosen system). The resulting encapsulated triplet‐azobenzene can relax back to the initial (*E*) state or undergo *E*→*Z* isomerization; in the latter case, the expulsion of *Z*‐azobenzene from **H** effectively removes it from the equilibrium. Regeneration of *E*‐azobenzene can be accomplished via direct excitation of the *Z* isomer with blue light, as in conventional systems (Figure 1a).^[^ ^41^ ^]^

We anticipated that the implementation of DESC in azopolymers would follow the same mechanism, provided that steric hindrance does not prevent the complexation between the azobenzene units and the supramolecular host. To systematically investigate the parameters governing the translation of DESC into polymers, we employed RAFT polymerization to ensure precise control over the polymer structure. We modified the azopolymer structure to tune its solubility and behavior in water. Our study centres on the interplay between the azopolymer, the supramolecular cage, and the photosensitizer, with the goal of maximizing the DESC efficiency and deriving general guidelines for its integration into more complex systems. Following a literature procedure^[^ ^43^ ^]^ we chose *N,N‐*dimethylacrylamide (DMA) as the main repeating unit, due to its good water solubility.

**Scheme 1:**
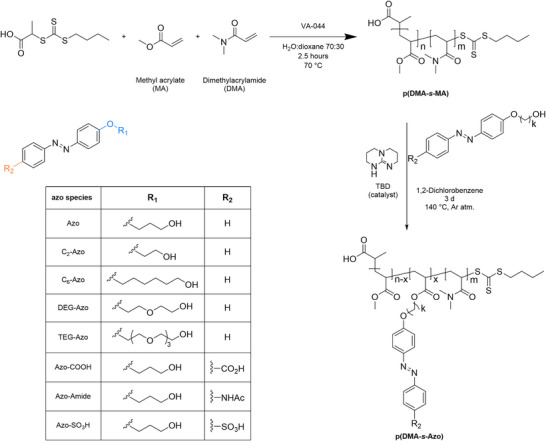
General route for the two‐step synthesis of p(DMA‐*s*‐Azo)‐copolymers and the structural formulas of azobenzene derivatives introduced as sidegroups.

We adopted a two‐step synthesis approach, introducing azobenzene side groups via post‐polymerisation modification. This strategy was inspired by the work of Sumerlin and co‐workers, who developed a facile organocatalytic method for functionalizing polyacrylates.^[^ ^44^ ^]^ To ensure this approach is feasible in our system, we first copolymerised methyl acrylate (MA) and DMA in a 1:10 molar ratio and monitored the monomer conversion over time (Figure S1). Similar conversion rates were observed for both monomers, suggesting an even distribution of the two monomers along the polymer backbone. For the post‐modification step, we synthesised C_6_‐Azo (see Scheme 1) containing an aliphatic hydroxy group. Sumerlin's procedure for the post‐modification of p(DMA_90_‐*s*‐MA_10_) yielded functionalisation efficiencies of 90% when conducted in 1,2‐dichlorobenzene at 140 °C for 3 days (Figure S2). Unreacted azobenzene was recovered and purified via flash column chromatography for further use.

**Figure 2:**
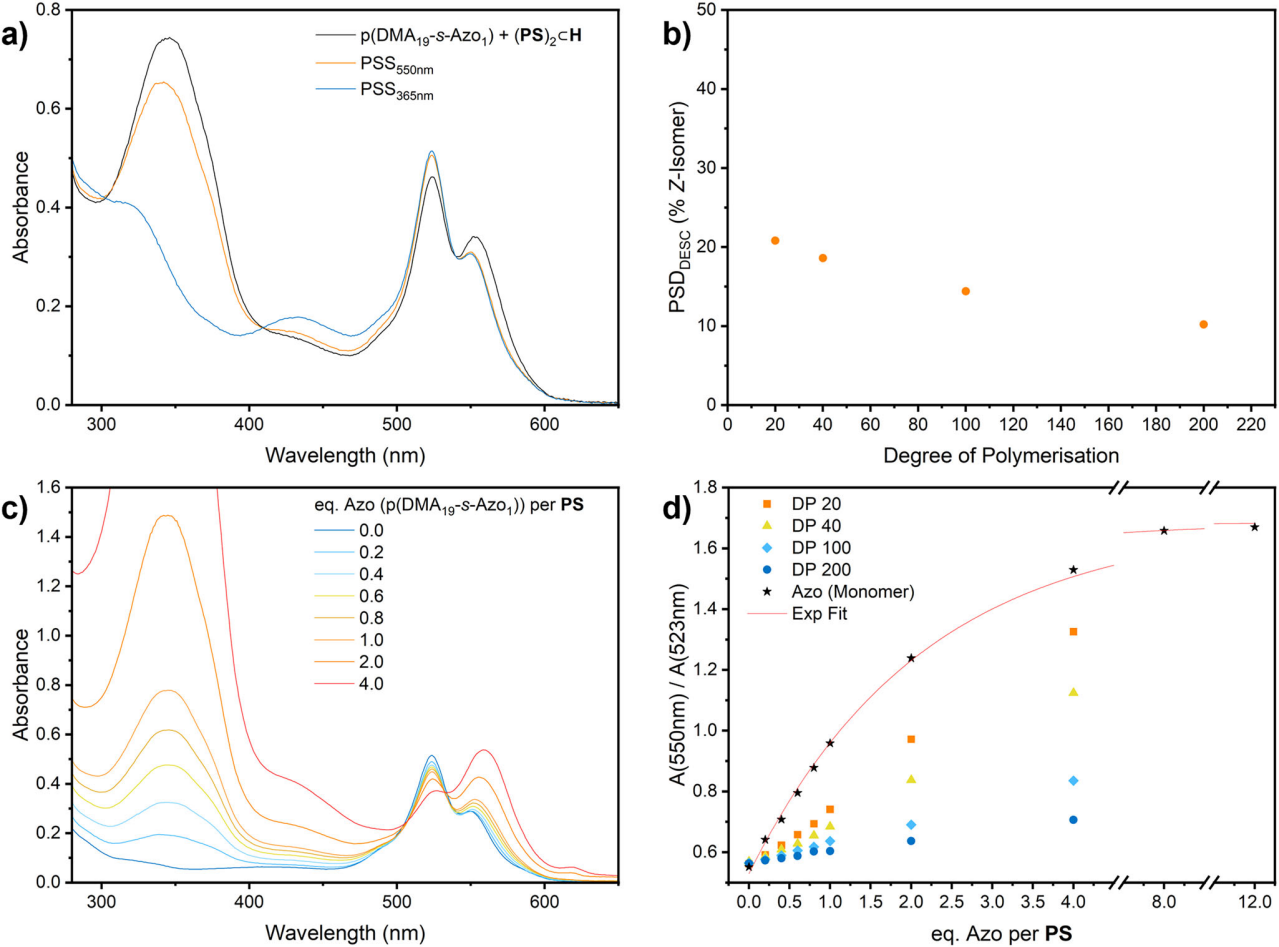
a) Absorption spectra of an aqueous p(DMA_19_‐*s*‐Azo_1_) / (**PS**)_2_⊂**H** (20 µM) solution with an **Azo**:**PS** ratio 1:1 (black) and the photostationary states after irradiation with yellow (550 nm) and UV (365 nm) light. b) Dependence of DESC efficiency on the degree of polymerization, keeping functionalization density at ∼5%. c) Absorption spectra of (**PS**)_2_⊂**H** (20 µM) in the presence of varying amounts of p(DMA_19_‐*s*‐Azo_1_). d) Ratios of absorbances of the (**Azo**·**PS**) heterodimer (*λ*_max_ = 550 nm) and the (**PS**)_2_ homodimer (*λ*_max_ = 523 nm) formed inside **H** during titration of polymers with varying DP and constant functionalisation density of 5%. Photoswitching and titration studies for the other polymers are shown in Figure S3.

With the synthesis of azopolymers established, we investigated their photoisomerization through DESC, starting with p(DMA_19_‐*s*‐Azo_1_). We studied its photoswitching behavior in water by UV–vis absorption spectroscopy (Figure 2a). For quantification of sensitized switching a combination of NMR‐ and absorption spectroscopy was employed. The photostationary distribution (PSD) obtained by direct excitation was determined by ^1^H NMR spectroscopy, yielding a high *Z*‐isomer content of 95%. Indirect (sensitized) isomerization was quantified by comparing absorbance changes upon irradiation with yellow (550 nm) light and UV (365 nm) light, referenced against the PSD_365nm_. Upon irradiation at 550 nm in the presence of one equivalent encapsulated **PS** in water, p(DMA_19_‐*s*‐Azo_1_) exhibited photoswitching, reaching a PSD of 21% *Z*‐isomer. In addition, notable changes were observed in the 500–550 nm range of the absorption spectrum; the peak at 523 nm, which is assigned to the BODIPY‐homodimer (**PS**)_2_⊂**H** increased, while the 550 nm peak, attributed to the (**PS**·**Azo**)⊂**H** heterodimer, diminished (Figure 2a). These spectral changes serve as an additional indicator of the host–azopolymer interaction and heterodimer formation, indicating accessibility of the azobenzene pendant units for the host **H**.

Next, we investigated the influence of DP on the DESC efficiency, using polymers with DP ranging from 20 to 200 while maintaining the azobenzene functionalization density at 5 mol%. Each of the polymers of this series (DP 20, 40, 100, 200) was subjected to photoisomerization in water, using a fixed azobenzene concentration of 40 µM and (**PS**)_2_⊂**H** concentration of 20 µM. Both direct excitation (365 nm) and DESC (550 nm) were employed. The obtained PSD_DESC_ displayed an inverse correlation with polymer length, decreasing from 21% *Z*‐isomer for p(DMA_19_‐*s*‐Azo_1_) to 10% for p(DMA_190_‐*s*‐Azo_10_). This corresponds to a 50% decrease in switching efficiency upon a tenfold increase in DP (Figure 2b). We attribute this trend to more pronounced coiling or entanglement and aggregation of longer polymer chains in solution, which may limit the accessibility of the supramolecular cage to the azobenzene units, thereby hindering heterodimer formation.

To study the supramolecular heterodimerization efficiency as a function of polymer chain length, we performed UV–vis titrations of all azopolymers against 20 µM (**PS**)_2_⊂**H** in aqueous solution. In addition to the expected increase in absorption at 350 nm caused by the increasing azobenzene content in the solution, titration led to an increase in absorption of the 550 nm band accompanied by a decrease of the band at 523 nm, similarly indicating the disassembly of the (**PS**)_2_⊂**H** homodimer at the expense of the (**Azo**·**PS**)⊂**H** heterodimer (Figure 2c). We plotted the ratio of absorbances between 550 nm and 523 nm as a function of the equivalents of azobenzene added per photosensitizer; the resulting saturation curves (Figure 2d) reveal the impact of DP on binding efficiency. For example, two equivalents of p(DMA_19_‐*s*‐Azo_1_) produce a similar extent of heterodimerization as 0.8 equivalents of the monomeric azobenzene in solution, or four equivalents of the longer‐chain p(DMA_38_‐*s*‐Azo_2_).

We quantified the heterodimer formation by fitting the titration data (Figures S14–S16) using the exponential function:

$$y=y_{0}+B\cdot e^{-\alpha x}$$

where *α* is a coefficient reflecting the efficiency of heterodimer formation. To facilitate comparison between the polymers, the *α* values were normalized to the corresponding fit obtained for the Azo monomer in solution. This approach enables direct assessment of the heterodimer formation for azobenzene units tethered to polymer chains, expressed as a percentage of the monomeric reference. Table 1 summarizes the findings for each polymer studied, pointing towards a clear trend of decreasing heterodimer formation efficiency with increasing DP.

**Table 1:** Structural characterization details of statistical copolymers before and after functionalization. Overview of DESC performance and heterodimer formation determined.

Polymer	DP (DMA / MA)^a)^	Azopolymer (post‐modified)	Mn_theor_ (g/mol)^b)^	Mn_SEC_ (g/mol)^c)^	Mw_SEC_ (g/mol)^c)^	*Đ* ^c)^	DESC efficiency (%)^d)^	PSD_DESC_ (mol% Z‐isomer)^e)^	Heterodimer formation tendency *α* (%)
p(DMA_19_‐*s*‐MA_1_)	20 / 1		2310	79	114	1.44			
p(DMA_38_‐*s*‐MA_2_)	41 / 2		4480	275	414	1.51			
p(DMA_95_‐*s*‐MA_5_)	91 / 4		9600	2040	2570	1.26			
p(DMA_190_‐*s*‐MA_10_)	186 / 8		19 400	6280	7590	1.21			
		p(DMA_19_‐*s*‐Azo_1_)	2530	292	396	1.35	22	21	43
		p(DMA_38_‐*s*‐Azo_2_)	4920	345	641	1.86	20	19	27
		p(DMA_95_‐*s*‐Azo_5_)	10 500	1780	2550	1.43	15	14	11
		p(DMA_190_‐*s*‐Azo_10_)	21 200	5320	7070	1.33	11	10	6
p(DMA_99_‐*s*‐MA_1_)	96 / 1		9760	1910	2360	1.23			
p(DMA_98_‐*s*‐MA_2_)	100 / 2		10 300	1900	2370	1.24			
p(DMA_97_‐*s*‐MA_3_)	97/3		10 100	n.d.	n.d.	n.d.			
p(DMA_95_‐*s*‐MA_5_)	91 / 4		9600	2040	2570	1.26			
p(DMA_90_‐*s*‐MA_10_)	96 / 9		10 500	1830	2290	1.25			
		p(DMA_99_‐*s*‐ Azo_1_)	10 100	2310	3290	1.43	43	41	35
		p(DMA_98_‐*s*‐ Azo_2_)	10 800	2120	2810	1.33	33	31	19
		p(DMA_97_‐*s*‐ Azo_3_)	10 800	1860	2350	1.27	21	19	16
		p(DMA_95_‐*s*‐ Azo_5_)	10 500	1780	2550	1.43	15	14	11
		p(DMA_90_‐*s*‐ Azo_10_)	12 600	720	1220	1.69	0	0	4
p(DMA_99_‐*s*‐MA_1_)	104 / 1		10 600	1730	2160	1.25			
		p(DMA_99_‐*s*‐[C_2_‐Azo]_1_)	10 800	2100	2940	1.40	43	41	22
		p(DMA_99_‐*s*‐Azo_1_)	10 900	2060	2760	1.34	54	51	35
		p(DMA_99_‐*s*‐[C_6_‐Azo]_1_)	10 900	1690	2710	1.60	43	41	29
		p(DMA_99_‐*s*‐[DEG‐Azo]_1_)	10 900	2140	2850	1.34	63	59	29
		p(DMA_99_‐*s*‐[TEG‐Azo]_1_)	11 000	n.d.	n.d.	n.d.	65	61	17
		p(DMA_99_‐*s*‐[Azo‐COOH]_1_)	10 900	2510	3380	1.35	39	37	124
		p(DMA_99_‐*s*‐[Azo‐SO_3_H]_1_)	10 900	2760	3570	1.29	85	77	180
		p(DMA_99_‐*s*‐[Azo‐Amide]_1_)	10 900	2450	3340	1.36	0	0	11
p(DMA_90_‐*s*‐MA_10_)	96 / 9		10 500	1830	2290	1.25			
		p(DMA_90_‐*s*‐MA_7_‐*s*‐[Azo‐SO_3_H]_3_)	11 400	3540	4400	1.24	71	64	

^a)^By ^1^H NMR spectroscopy of the isolated polymer in CDCl_3_.

^b)^By ^1^H NMR spectroscopy of the purified polymer.

^c)^By SEC (DMSO + 0.01 M LiBr, poly(sodium styrene sulfonate) standard, see Figure S16).

^d)^In relation to direct excitation of the 1/1 mixture of azobenzene and **PS**.

^e)^By multiplying the DESC efficiency and PSD_365nm_ obtained by NMR.

Following the alteration of DP, we studied the effect of azobenzene functionalization density on DESC performance, using polymers with DP 100 and functionalization densities from 1 to 10%. Titrations against (**PS**)_2_⊂**H** revealed a strong correlation between the homodimer/heterodimer equilibrium and azobenzene content (Figure S4). Polymers with lower azobenzene content favor the formation of (**Azo**·**PS**)⊂**H**. At 1% functionalization density, the degree of heterodimer formation reaches 36% compared to the (**Azo**)_2_⊂**H** homodimeric complex. This value is reduced to 4% at the functionalization density of 10%. In line with the findings on heterodimer formation efficiency, the DESC efficiency depended strongly on the functionalization density (Figure S5). While p(DMA_90_‐*s*‐Azo_10_) exhibited a poor switching efficiency of 2%, p(DMA_99_‐*s*‐Azo_1_) yielded 43% switching relative to direct excitation, corresponding to a PSD of 41% *Z* isomer. We hypothesize that this pronounced dependency arises from polymer aggregation and increased hydrophobicity at higher azobenzene contents. The results are supported by dynamic light scattering (DLS) analysis, which revealed a significant decrease in the critical aggregation concentration (CAC) from 0.05 to 0.006 mg·mL^−1^ with increasing functionalization density (from 1% to 10%, Figure S6). We speculate that this trend is linked to the formation of more stable aggregates at higher functionalization densities, which may hinder the exchange kinetics.

**Figure 3:**
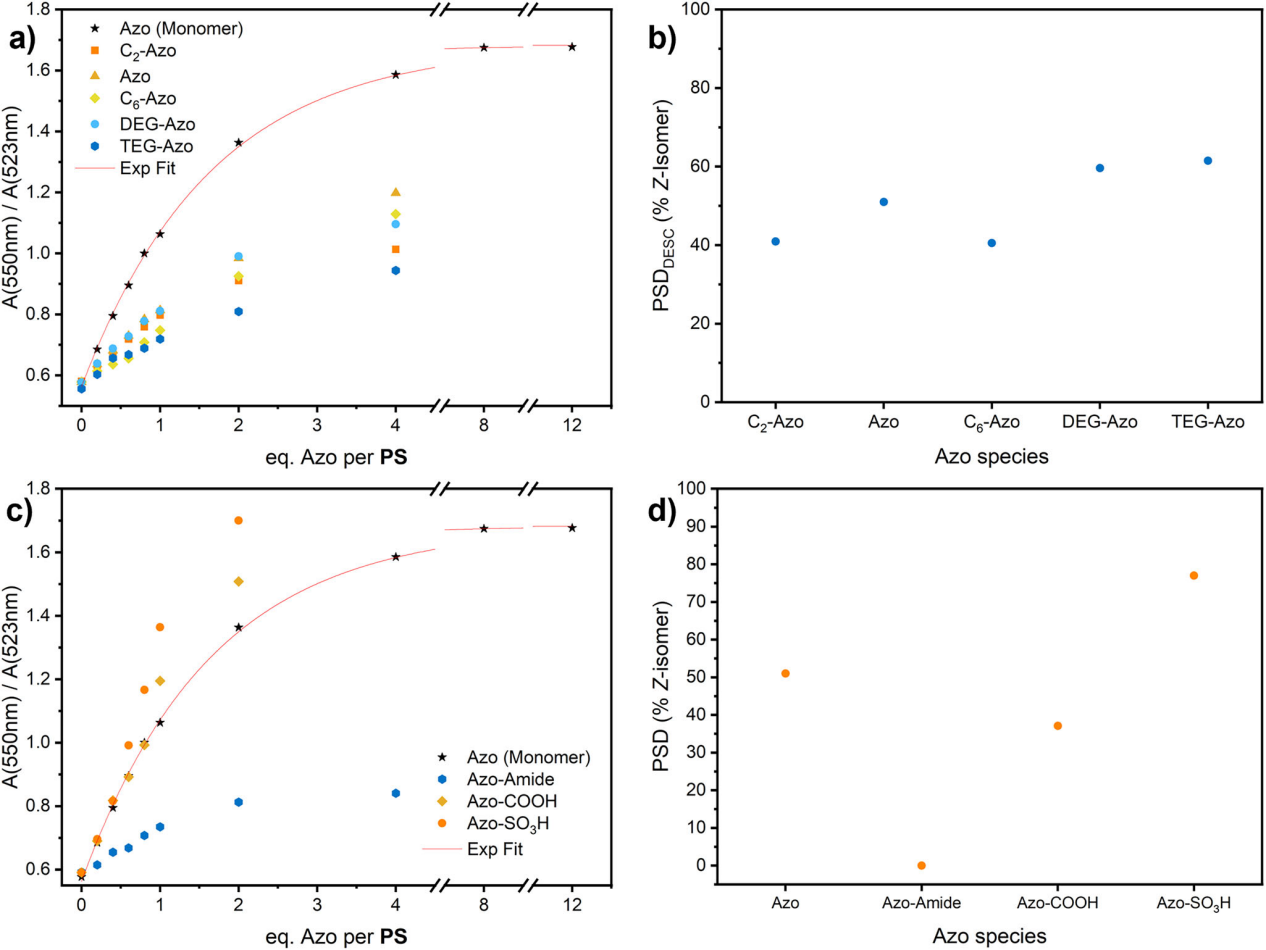
Dependence of the heterodimer formation efficiency and DESC efficiency on structural variations of the azobenzene side chains, at DP 100 and a functionalisation density of 1%. a) Ratios of absorbances of (**Azo**·**PS**) heterodimers (550 nm) and the (**PS**)_2_ homodimer (523 nm) within **H** during titration with polymers having different linkers between azobenzene and the polymer backbone. b) Dependence of the DESC efficiency on the identity of the linker between azobenzene and the polymer backbone. c) Ratios of absorbances of (**Azo**·**PS**) heterodimers (550 nm) and the (**PS**)_2_ homodimer (523 nm) within **H** during titration of polymers with different hydrophilic substituents on azobenzene. d) Dependence of DESC efficiency on the hydrophilic substituent on azobenzene. For the UV–vis spectra, see Figures S7–S10.

As the above observations suggest a direct relationship between hydrophilicity and DESC efficiency, we sought to investigate the effect of modifying the azobenzene units. To this end, we incorporated different spacers between the azobenzene and the polymer backbone, differing in both length and polarity, and modified the terminal phenyl ring with hydrophilic functional groups, such as carboxylic acid and acetylamide. These changes were undertaken to promote polymer–solvent interactions while simultaneously reducing potential intra‐ and intermolecular interactions between the azobenzene units. Overall, these modifications aim to increase the propensity for **Azo**–**H** interactions and hence, the DESC efficiency. The structural formulae of the synthesized azobenzene derivatives are given in Scheme 1. Polymers with DP100 and a functionalization density of 1% were studied. Modifying the spacer between the azobenzene and the polymer backbone had little effect on the homodimer/heterodimer equilibrium. The observed variations in *α* ranged mostly between 29% and 35%, with a few outliers at 17% (TEG‐Azo) and 22% (C_2_‐Azo) (Figure 3a). However, upon irradiation, distinct differences in DESC efficiency emerged. Polymers with the hydrophilic di(ethylene glycol) (DEG) and tetra(ethylene glycol) (TEG) spacers reached higher PSDs between 59% and 61% *Z* isomer, respectively, whereas the previously used Azo with C_3_‐spacer yielded 51% (Figure 3b). Interestingly, while showing the highest DESC efficiency, heterodimer formation with p(DMA_99_‐*s*‐[TEG‐Azo]_1_) is least pronounced, at 17%. We postulate that the TEG linker increases the accessibility of the azo moiety to **H** while decreasing the interaction strength and thus shifting the equilibrium between (**PS**)_2_⊂**H** and (**Azo**·**PS**)⊂**H** to the homodimer, presumably due to the higher polarity of the linker. As previously shown, minute amounts of heterodimer at equilibrium can afford near‐quantitative *E*→*Z* isomerization owing to the rapid guest exchange.^[^ ^41^ ^]^ Other aliphatic spacers (C_2_ and C_6_) yielded slightly lower conversions of up to 41% of the Z‐isomer.

The introduction of hydrophilic terminal substituents onto the azobenzene scaffold yielded more complex correlations between its structure and DESC efficiency (Figure 3c and d). p(DMA_99_‐*s*‐[Azo‐COOH]_1_) exhibited significant heterodimer formation and appeared to displace **PS** from the cage, as evidenced by the occurrence of turbidity and the appearance of an absorption band at 625 nm upon addition of ≥ 0.6 equivalents of azobenzene (Figure S9). The 625 nm band is characteristic of *J*‐aggregates of BODIPY.^[^ ^42^ ^]^ The addition of excess “empty” host **H** suppressed the precipitation of **PS** and enabled the acquisition of the saturation binding curve. This curve exhibited a notably steeper slope compared to the Azo‐monomer reference, with an apparent *α*‐value of 124%, indicating a high binding propensity. However, under 550 nm irradiation, DESC‐triggered isomerization (**Azo**:**PS **= 1:1) yielded only 39% conversion in relation to direct 365 nm excitation. We assume that the apparently low conversion is predominantly caused by the competition between the *E*→*Z* isomerization via DESC and the *Z*→*E* relaxation, which can both occur under irradiation with yellow light (550 nm) in the presence of (**PS**)_2_⊂**H**, limiting the net conversion and the apparent DESC efficiency. To support this hypothesis, we performed irradiation studies of p(DMA_99_‐*s*‐[Azo‐COOH]_1_) in the absence of any photosensitizer (Figure S11). Indeed, these experiments showed significantly faster recovery of the π–π* absorption band at 360 nm upon irradiation with yellow light compared to the previously used azobenzenes. Similarly, p(DMA_99_‐*s*‐[Azo‐Amide]_1_) does not exhibit any observable *E*→*Z* isomerization under DESC conditions (Figure S10b), presumably due to the combination of 1) poor host–guest compatibility (and thus suppressed formation of the heterodimer; Figure S9b) and 2) fast relaxation of the potentially formed *Z* isomer under 550 nm light (Figure S11).

The incorporation of Azo‐SO_3_H into the polymer proved more difficult, requiring harsher reaction conditions and a larger excess of the nucleophile and catalyst, resulting in a modest modification yield of ca. 25% (yielding one azobenzene group per four chains on average). However, p(DMA_99_‐*s*‐[Azo‐SO_3_H]_1_) was identified as a highly promising candidate for DESC. We found that it exhibits a strong interaction with the host **H**, with an 80% increase in the tendency to form heterodimers compared to the azobenzene‐monomer. It also showed a remarkably efficient isomerization via DESC, with about 85% of the switching efficiency obtained through direct excitation, corresponding to 77% *Z*‐isomer in the PSD. To ensure that the high efficiency is not only caused by the low azobenzene content, we also prepared p(DMA_90_‐*s*‐MA_7_‐*s*‐[Azo‐SO_3_H]_3_) from p(DMA_90_‐*s*‐MA_10_) and characterized it. This polymer achieved 63% *Z*‐isomer content via DESC, which is 71% of that obtained through direct excitation. These results clearly demonstrate a general improvement in switching efficiency, even at higher functionalization densities. Interestingly, the dependency of DESC efficiency on functionalization density in p(DMA‐*s*‐[Azo‐SO_3_H]) appears less pronounced compared to p(DMA‐*s*‐Azo), where a three‐fold increase in functionalization led to a 50% reduction in indirect isomerization efficiency (see Table 1).

**Figure 4:**
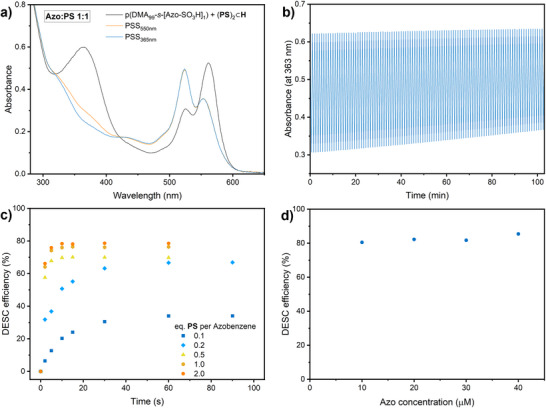
Photophysical analysis of p(DMA_99_‐*s*‐[Azo‐SO_3_H]_1_) switching using DESC. a) Absorption spectra of p(DMA_99_‐*s*‐[Azo‐SO_3_H]_1_) + (**PS**)_2_⊂**H** (20 µM) solution with an **Azo**:**PS** ratio 1:1 and subsequent changes upon irradiation with yellow (550 nm) light and UV (365 nm) light. b) Following the absorbance at 363 nm during 100 switching cycles using DESC. c) Progression of *E*→*Z* isomerisation via DESC depending on irradiation time and **PS** loading at the fixed azobenzene concentration of 40 µM. d) Dependence of DESC efficiency on azobenzene concentration.

To assess the robustness of p(DMA_99_‐*s*‐[Azo‐SO_3_H]_1_), we subjected it to 100 switching cycles of alternating irradiation with yellow (550 nm, 30 s) and blue (435 nm, 30 s) light. The switching process was monitored by following the absorbance at 363 nm, the wavelength of maximum absorbance of the π–π* band (Figure 4b). Over these cycles, essentially no change in the *Z→E* isomerization efficiency was observed, with the absorbance after 435 nm irradiation ranging from 0.62 to 0.63. However, the efficiency of DESC reduced over time, as evidenced by a gradual increase in absorbance from 0.31 to 0.37 after the 100 switching cycles, corresponding to a 10% decrease in the *Z*‐isomer content at the PSD. Comparison of the absorption spectra of the first and last switching cycle of the repeated experiment (Figure S12) revealed that the absorption bands of the photosensitizer decreased by 40% over the total light exposure of 2 h, while the absorption in the UV region increased by up to 67%, indicating photodegradation of **PS**. Indeed, direct *E*→*Z* isomerization using UV (365 nm) light performed after these irradiation cycles confirmed that the performance of the photoswitch remained practically unchanged. Thus, we conclude that the main factor in the loss of DESC efficiency is related to bleaching of the **PS**. We also studied the effect of **PS** loading on switching efficiency by varying it from 0.1 to 2.0 equivalents with respect to azobenzene (Figure 4c). Both the isomerization rate and the *Z*‐isomer population in the PSD increase with higher **PS** content, ranging from 34% at 0.1 equivalents to 79% at 2.0 equivalents, showcasing that the *Z*‐isomer content of 77% can be further enhanced under optimized conditions. The obtained PSD with 0.5 equivalents of **PS** amounts to 70% *Z‐*isomer, which is also in relatively good agreement with the above reduction in DESC efficiency after 100 irradiation cycles. Interestingly, PSD_DESC_ decreases slightly at lower concentrations (Figure 4d). This effect was even more pronounced in p(DMA_99_‐*s*‐[DEG‐Azo]_1_), which was studied under similar conditions and consistently exhibited the same trends (Figure S13). We postulate that, owing to the relatively low binding constants^[^ ^42^, ^45^ ^]^ the fraction of azobenzene within **H** at this concentration regime increases at higher concentrations, leading to higher PSDs.

Our results prove that DESC can be successfully integrated into water‐soluble, dimethylacrylamide‐based azopolymers. While a low DP and even more so a low azobenzene content are generally beneficial for maximizing DESC efficiency, a broad substrate tolerance can be achieved through strategic modification to the polymer structure. An increase in one of these parameters can be partially compensated for by reducing the other, allowing flexible tuning over polymer properties. Modification of the azobenzene structure, particularly increasing its hydrophilicity, further enhances the DESC efficiency in polymeric systems. These modifications increase the overall DESC efficiency to nearly match the performance under direct excitation with UV light while retaining reasonably good robustness over at least 100 switching cycles. An optimal system contains a hydrophilic linker between the azobenzene and the polymer backbone, and a hydrophilic (sulfonate) terminal group on the azobenzene, while maintaining spectral separation between the azobenzene's and photosensitizer's absorption bands. Still, the observed attenuation upon repeated stimulation remains a challenge. This issue might possibly be overcome by selecting different polymer architectures and changing their assembly behavior, thereby ensuring reliable complex formation between the host and guests. Also, the feasibility of combining DESC with other supramolecular motifs for stronger responses upon irradiation remains to be investigated.

## Conclusion

To summarize, we have thoroughly studied the visible‐light‐sensitized *E*→*Z* isomeriation of azobenzenes in macromolecular systems via the recently introduced DESC strategy and optimized the process by systematically tailoring the polymer structure. In general, the hydrophilicity and length of the polymer chain, as well as its assemblies, determine DESC performance. While low degrees of polymerization and low functionalization densities are beneficial for sensitized isomerization, an increase in one of those parameters could be off‐set by altering the other, allowing for structural adjustments. Structural modifications of the azobenzene scaffold further influenced sensitized switching. In particular, incorporating a sulfonic acid group was found to significantly enhance DESC, yielding isomerization performance close to that under direct excitation, with little fatigue over at least 100 switching cycles.

We found that the interaction between the azopolymer and the host is primarily determined by the same factors that govern sensitized isomerization. Thus, synergistic optimization of both host–guest interaction and azobenzene addressability via sensitization is vital for designing visible‐light‐responsive complex supramolecular assemblies and materials. We believe that the principles developed herein can be generally applied to other aqueous macromolecular systems. Depending on the system's intrinsic properties, even higher DESC performance is probably achievable. The understanding gained in this study will pave the way for the development of complex crosslinked systems and soft materials driven entirely by visible light.

## Supporting Information

The authors have cited additional references within the Supporting Information.^[^ ^45^ ^]^

## Conflict of Interests

The authors declare no conflict of interest.

## Supporting information



Supporting Information

## Data Availability

The data that support the findings of this study are available in the Supporting Information of this article.
