# Supplementary material for: Sensitized Disequilibration of Water‐Soluble Azopolymers
Source: Angew Chem Int Ed Engl. 2025 Dec 23;65(7):e23447. doi: 10.1002/anie.202523447 (PMC12887633; doi:10.1002/anie.202523447)
Supplement: Supplementary file 1 — Supporting Information [file ANIE-65-e23447-s001.pdf]

Supporting Information for

# Sensitised Disequilibration of Water-Soluble Azopolymers

Henning Jörn Meteling[a], Julius Gemen[b], Satu Häkkinen[a], Rafal Klajn[c], Arri Priimagi*[a]

[a] H. J. Meteling, Prof. Dr. S. Häkkinen, Prof. Dr. A. Priimagi
Faculty of Engineering and Natural Sciences
Tampere University
P.O. Box 541, Tampere FI-33101, Finland
E-mail: arri.priimagi@tuni.fi

[b] Dr. J. Gemen
Organisch-Chemisches Institut
University of Münster
Correnssstrasse 36, 48149 Münster

[c] Prof. Dr. R. Klajn
Institute of Science and Technology Austria (ISTA)
Am Campus 1, 3400 Klosterneuburg, Austria

1. Development of copolymerization 2
2. Photophysical characterization of azopolymers 4
3. Materials and methods 19
4. Synthesis of Azobenzene derivatives and polymers 22
5. Supplementary references 34
6. NMR-spectra + SEC traces 35

# 1. Development of copolymerization

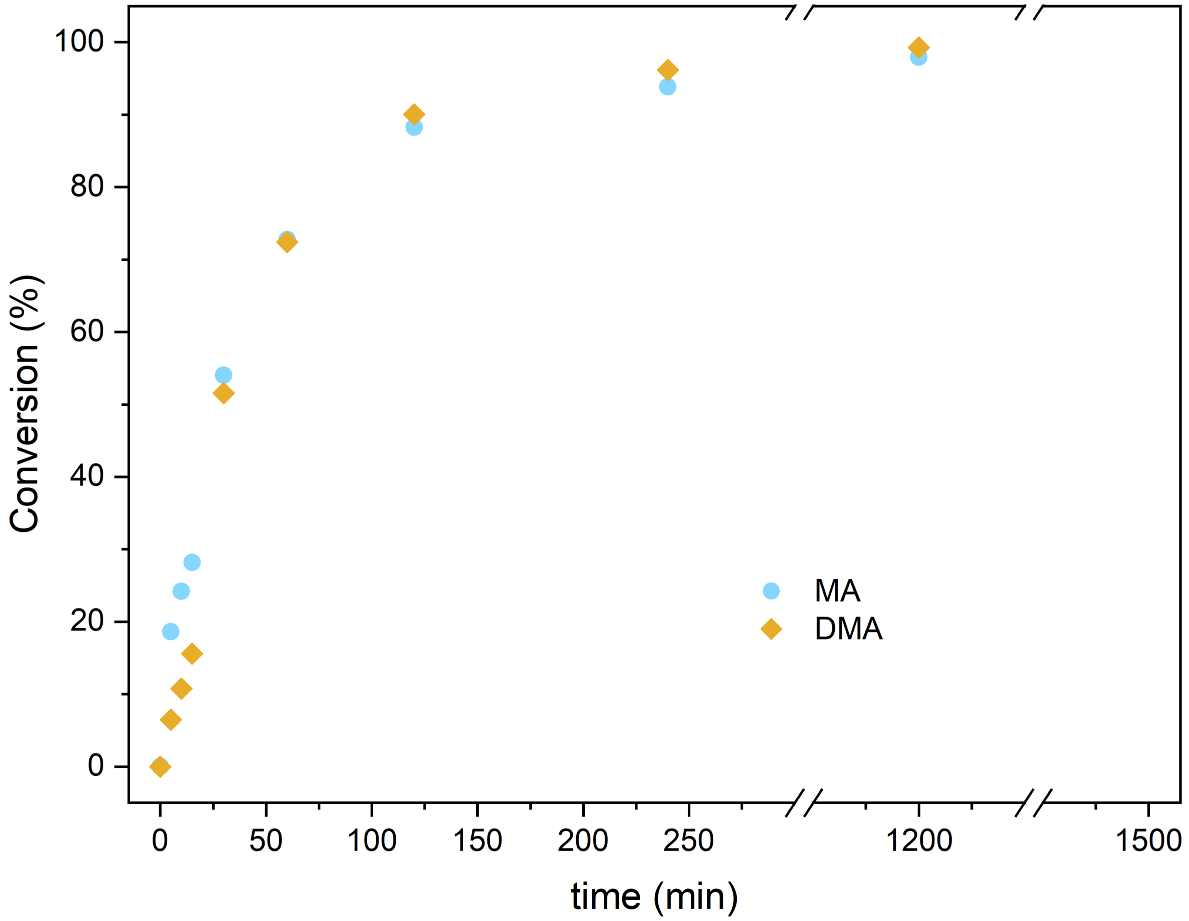


Figure S1: Conversion time plots for the copolymerisation of DMA and MA at 70 °C, with AIBN as initiator and PABTC as CTA, obtained by $^1$H NMR spectroscopy.

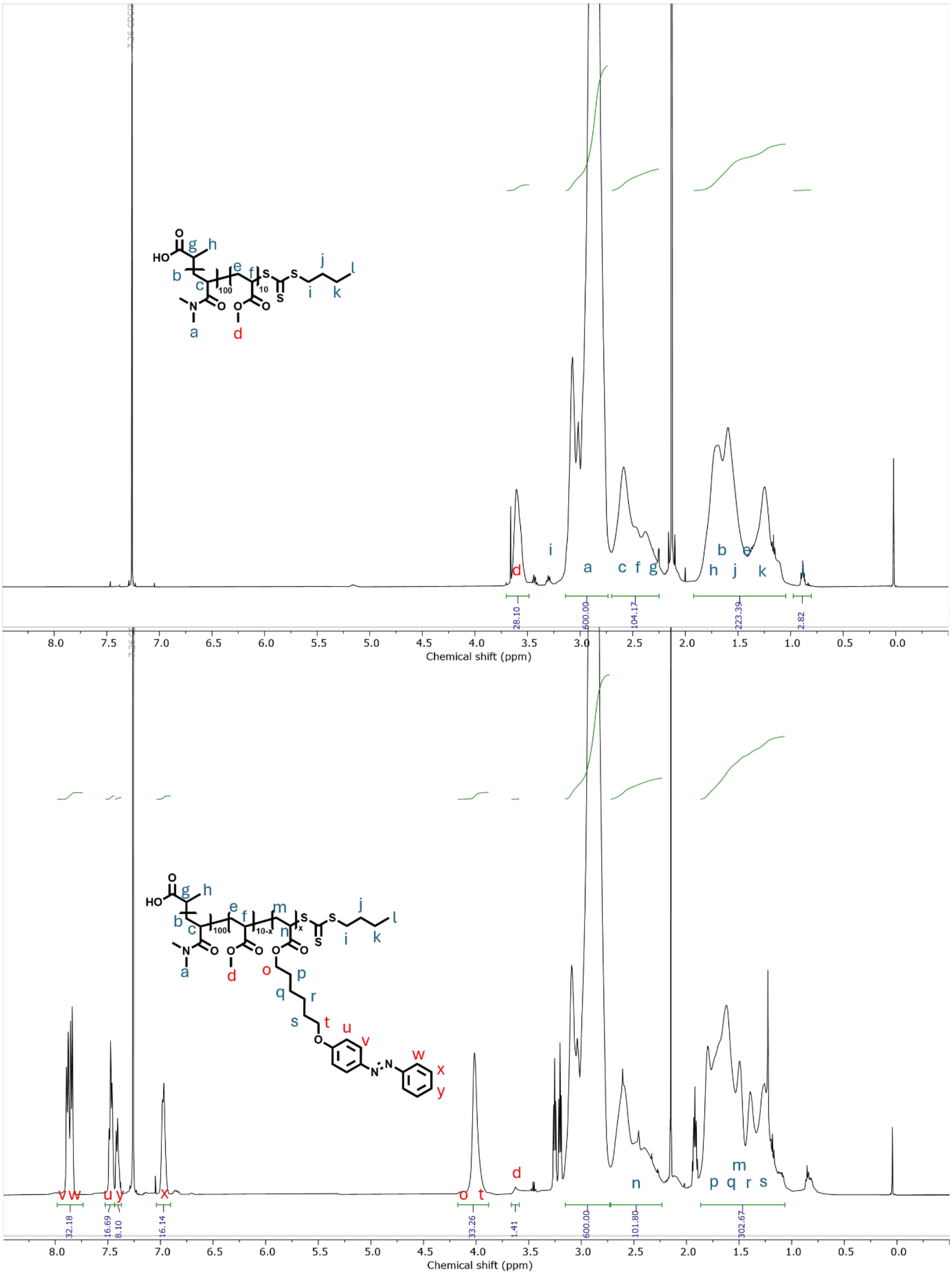


Figure S2: [1]H NMR spectra following the post-modification of p($DMA_{100}$-s-$Azo_{10}$) with $C_6$-Azo, with the peaks to determine modification efficiency assigned in red.

TUNI Luottamuksellinen - Confidential (3Y)

## 2. Photophysical characterisation of azopolymers

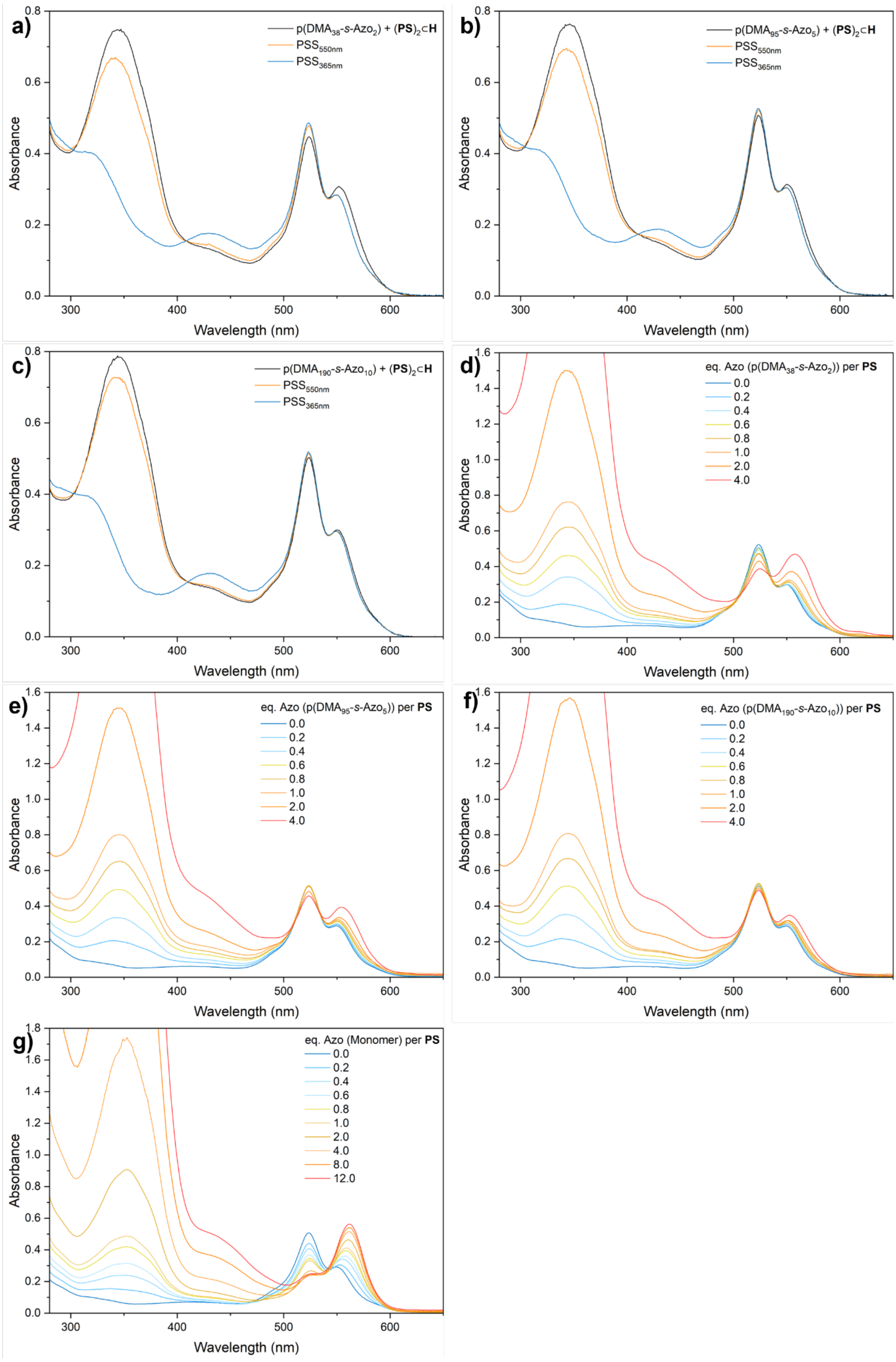


Figure S3: a) – c) Switching studies of azopolymers with varying DP (5 % Azo density) in a 20 µM (**PS**)$_2$⊂**H** solution with Azo/**PS** ratio: 1/1. d) – g) Titration of azopolymers with varying DP and Azo-monomer against a 20 µM solution of (**PS**)$_2$⊂**H**. Fitting curves are shown in Figure S13.

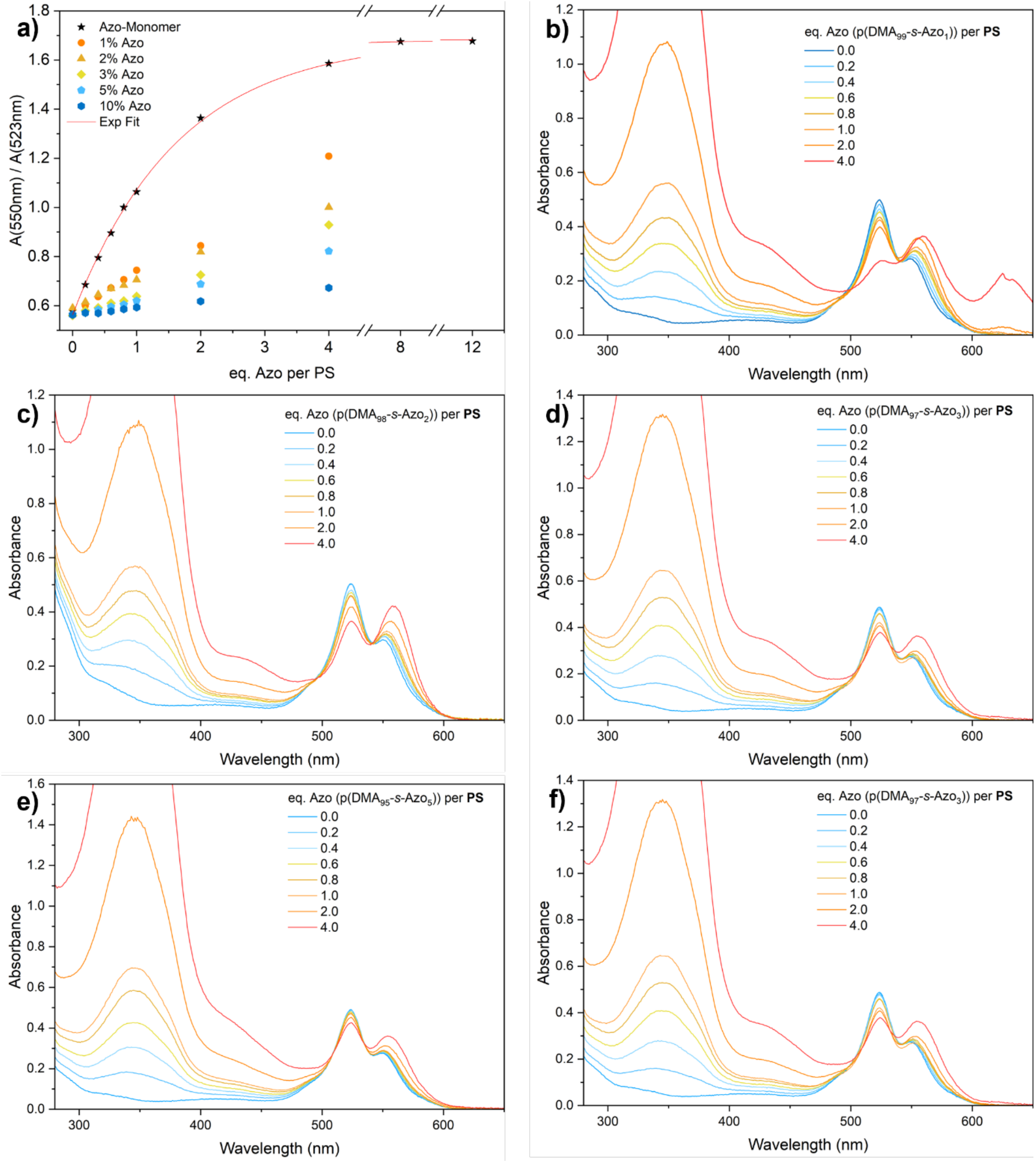


Figure S4: a) Ratios of absorbances of (**PS**·Azo) heterodimer (550 nm) and $(\mathbf{PS})_2$ homodimer (523 nm) inside **H** during titration of polymers with varying functionalisation density and constant DP = 100. b–f) Titration of azopolymers with varying functionalisation density against $(\mathbf{PS})_2 \subset \mathbf{H}$ (20 µM). Fitting curves are shown in Figure S14

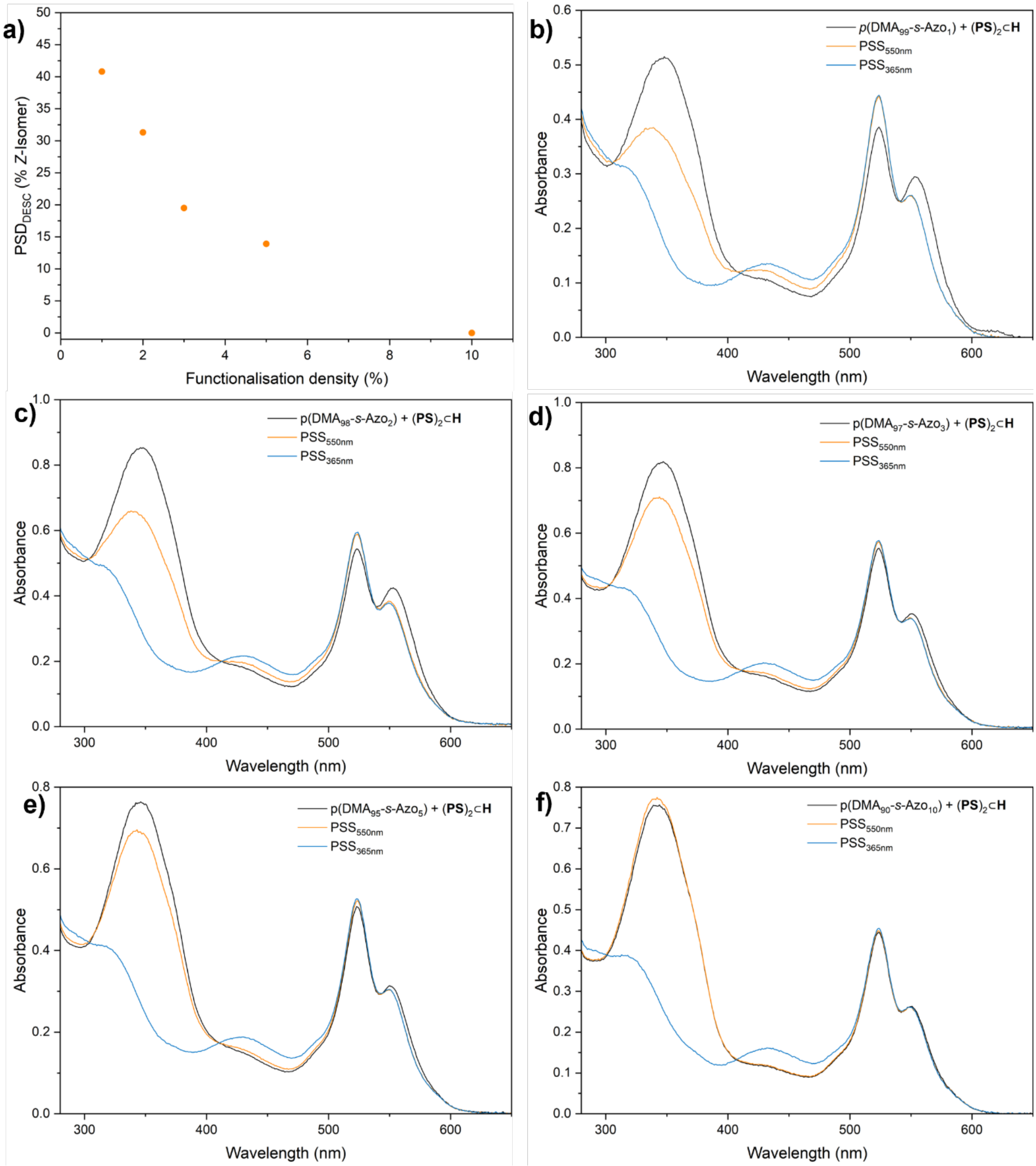


Figure S5: a) Dependence of DESC efficiency on the functionalisation density, keeping DP at 100. b–f) Switching studies of azopolymers with varying functionalisation density (DP 100) in a 20 μM (**PS**)$_2$⊂**H** solution with Azo/**PS** ratio: 1/1.

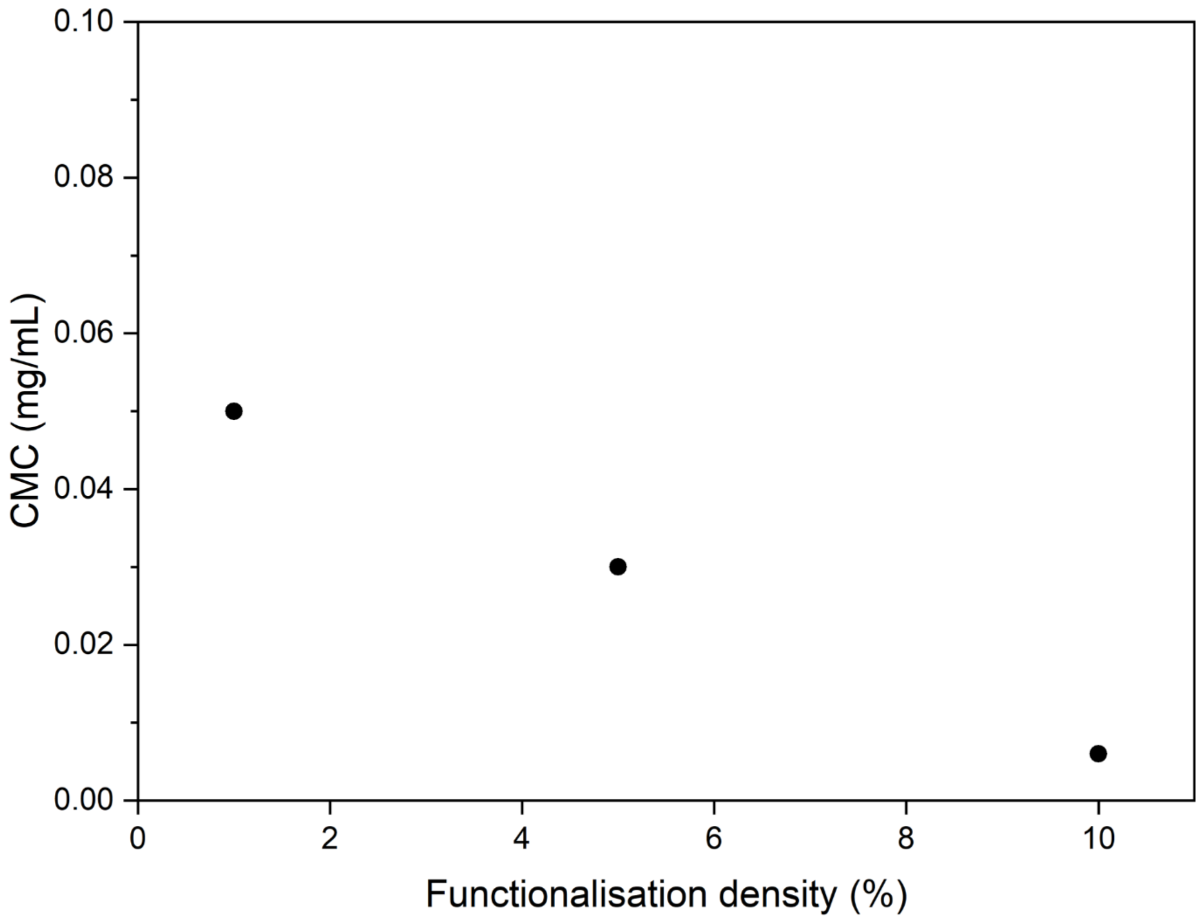


Figure S6: Determined CACs depending on functionalisation density of azopolymers with DP = 100, obtained by DLS analysis.

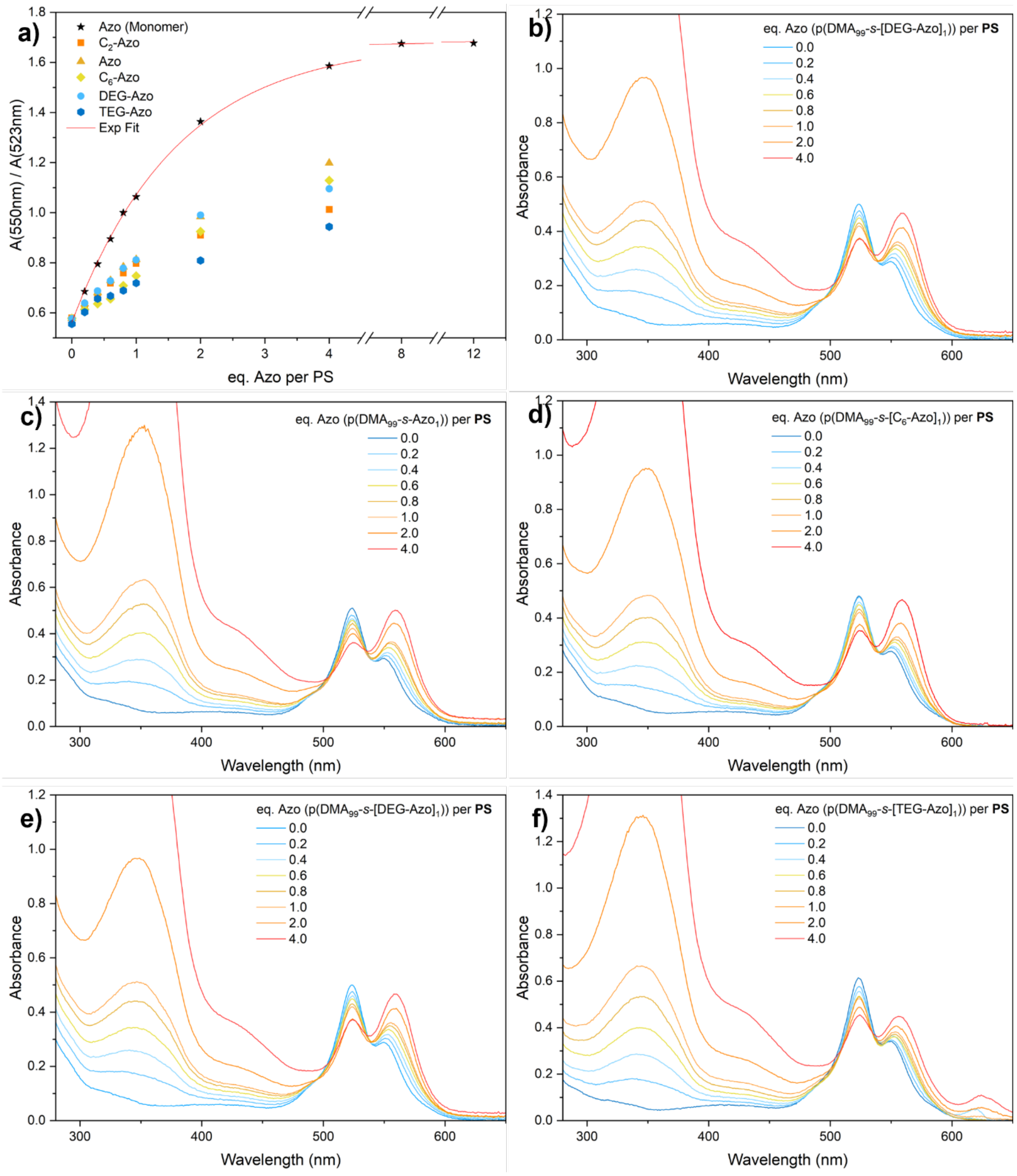


Figure S7: a) Ratios of absorbances of (**PS**·Azo) heterodimer (550 nm) and (**PS**)$_2$ homodimer (523 nm) formed inside **H** during titration of polymers with different spacers between azobenzene and polymer backbone, keeping functionalisation density (1%) and DP (100) constant. b–f) Titration of azopolymers with different spacers against (**PS**)$_2$⊂**H** *(*20 μM*)*. Fitting curves are shown in Figure S15.

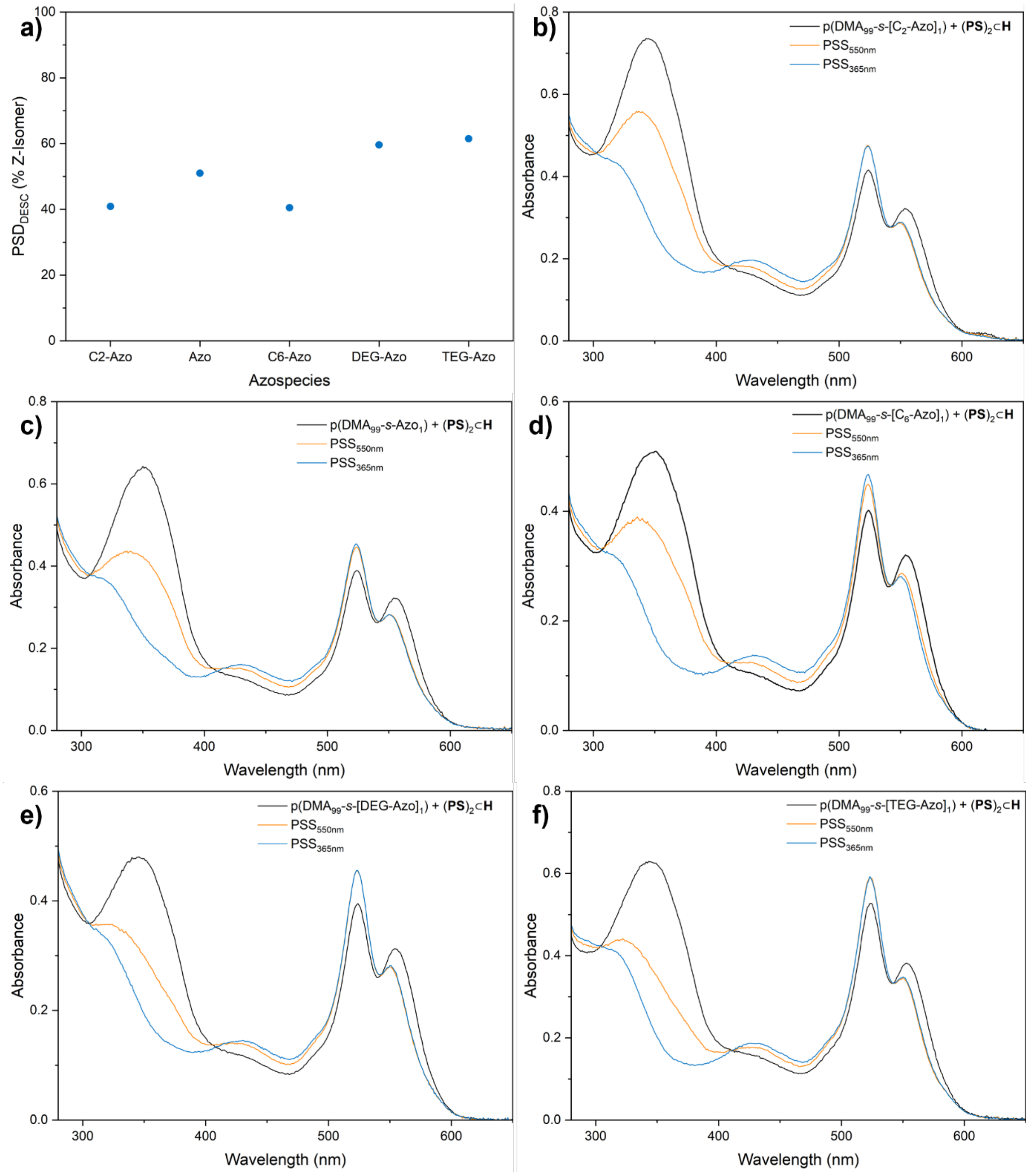


Figure S8: Dependence of DESC efficiency on the spacer between the polymer backbone and azobenzene, keeping DP at 100 and functionalisation density at 1%. b–f) Switching studies of azopolymers with varying spacers in a 20 µM (**PS**)$_2$⊂**H** solution with Azo/**PS** ratio = 1/1.

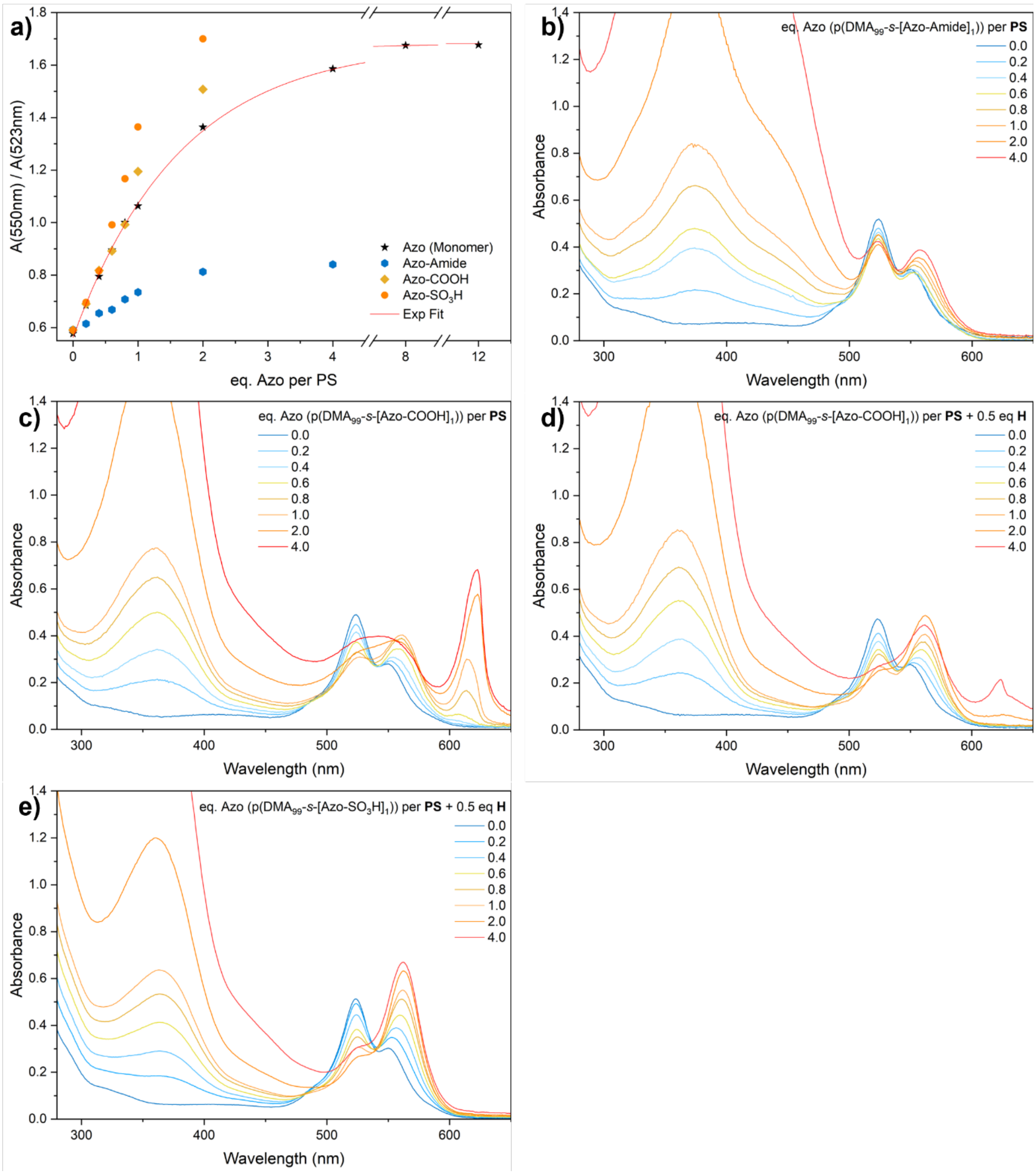


Figure S9: a) Ratios of absorbances of (**PS**·Azo) heterodimer (550 nm) and $(\mathbf{PS})_2$ homodimer (523 nm) formed inside **H** during titration of polymers with different hydrophilic substituents at the azobenzene, keeping functionalisation density (1%) and DP (100) constant. b–e) Titration of azopolymers with different hydrophilic functional groups against $(\mathbf{PS})_2{\subset}\mathbf{H}$ (20 µM). Fitting curves are shown in Figure S15.

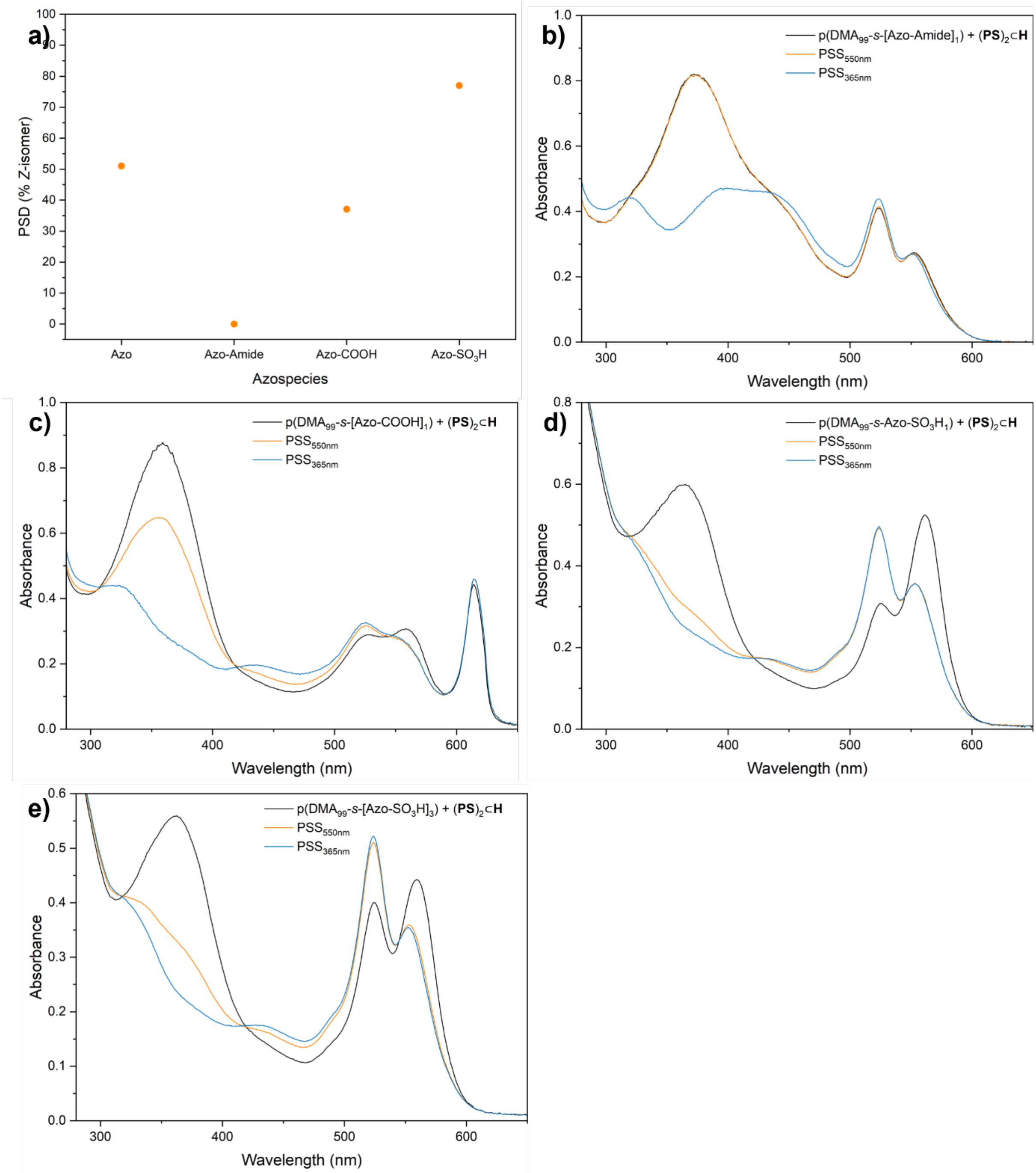


Figure S10: Dependence of DESC efficiency on the hydrophilic substituent at the azobenzene, keeping DP at 100 and functionalisation density at 1%. b–e) Switching studies of azopolymers with different hydrophilic substituents at the azobenzene in a 20 µM (**PS**)$_2$⊂**H** solution with Azo/**PS** ratio = 1/1.

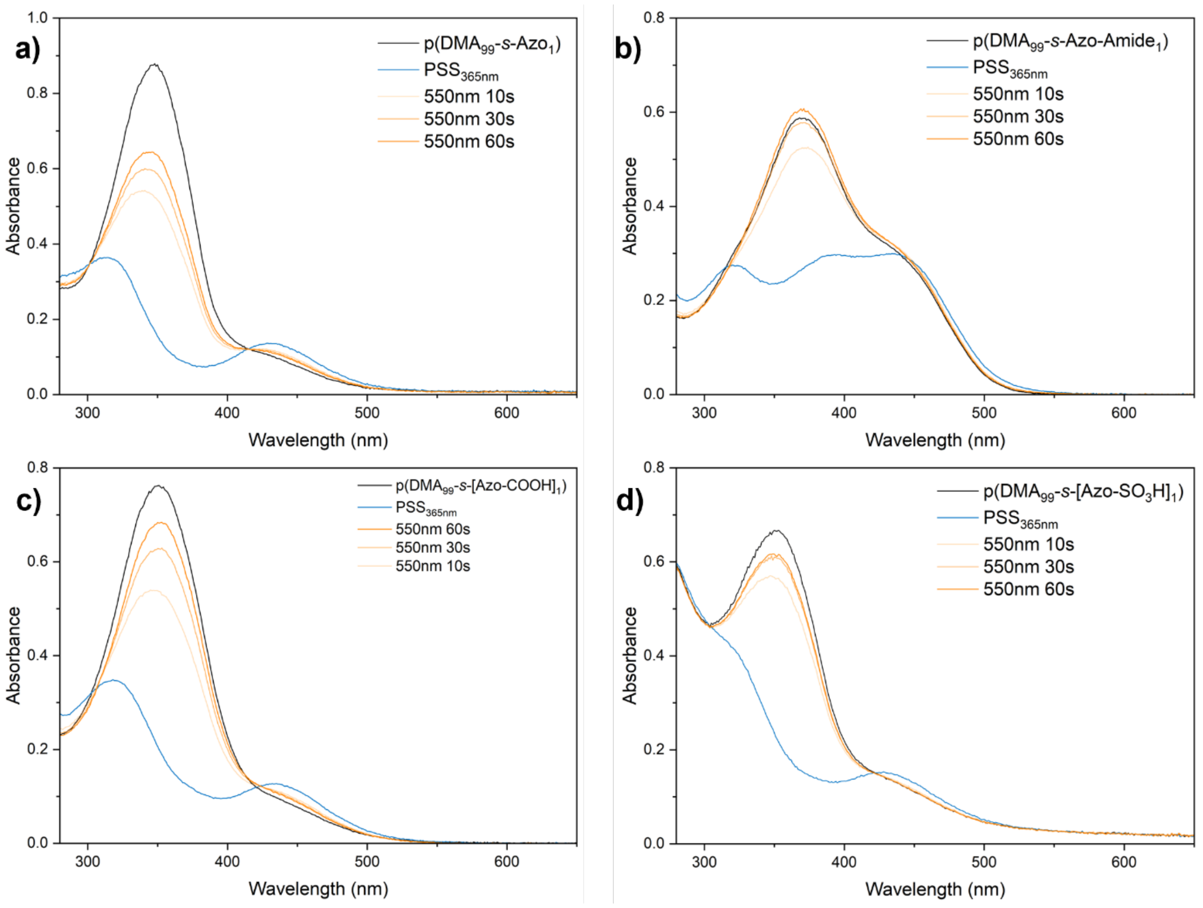


Figure S11: Switching studies of a) p(DMA$_{99}$-*s*-Azo$_1$), b) p(DMA$_{99}$-*s*-[Azo-Amide]$_1$), c) p(DMA$_{99}$-*s*-[Azo-COOH]$_1$) and d) p(DMA$_{99}$-*s*-[Azo-$SO_3H$]$_1$) in the absence of a photosensitiser.

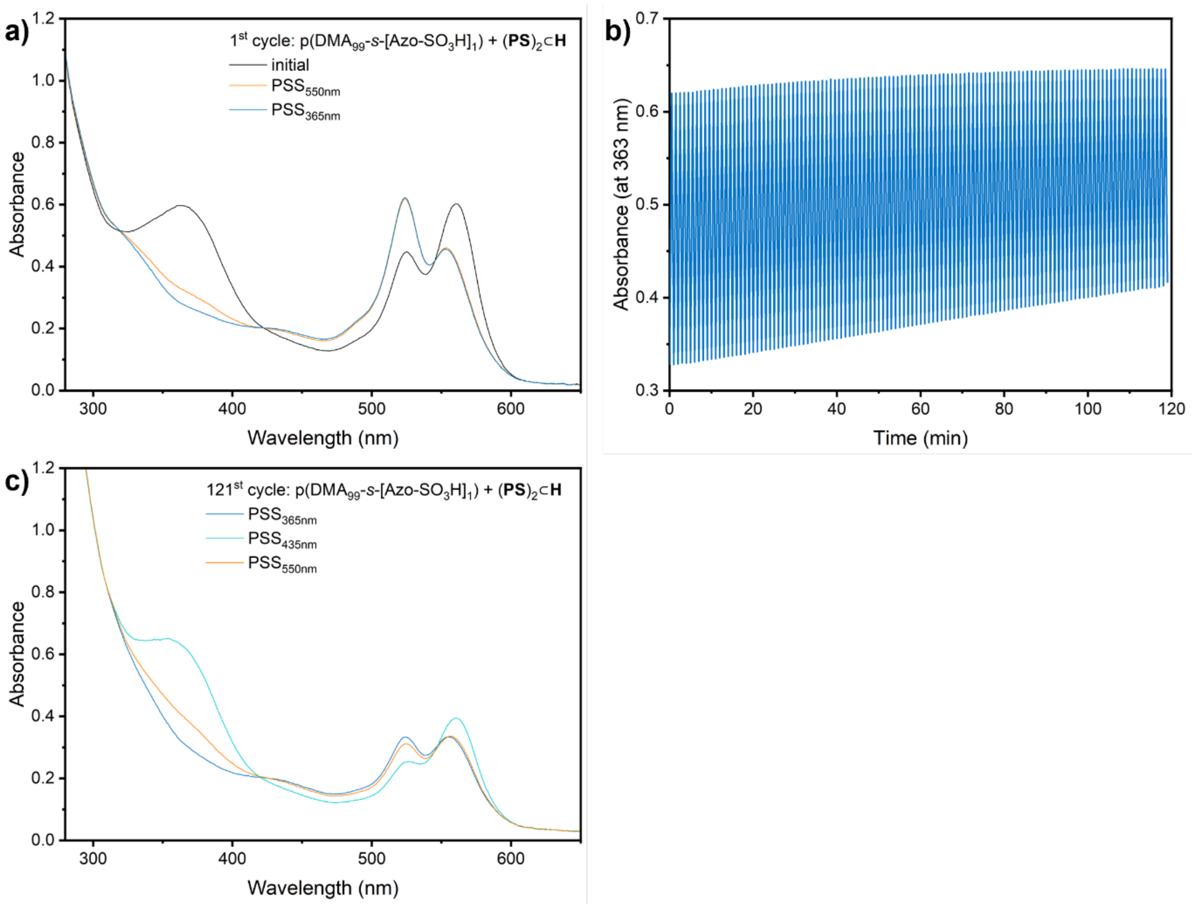


Figure S12: DESC-stability studies over prolonged periods of irradiation with p($DMA_{99}$-*s*-[Azo-$SO_3H$]$_1$) + (**PS**)$_2$⊂**H** (20µM) with an azobenzene:**PS** ratio of 1:1. a) Absorption spectra during the first cycle of irradiation. b) Trace of the absorbance at 363 nm while performing 120 switching cycles using DESC. c) Absorption spectra during the 121$^{st}$ photoswitching cycle.

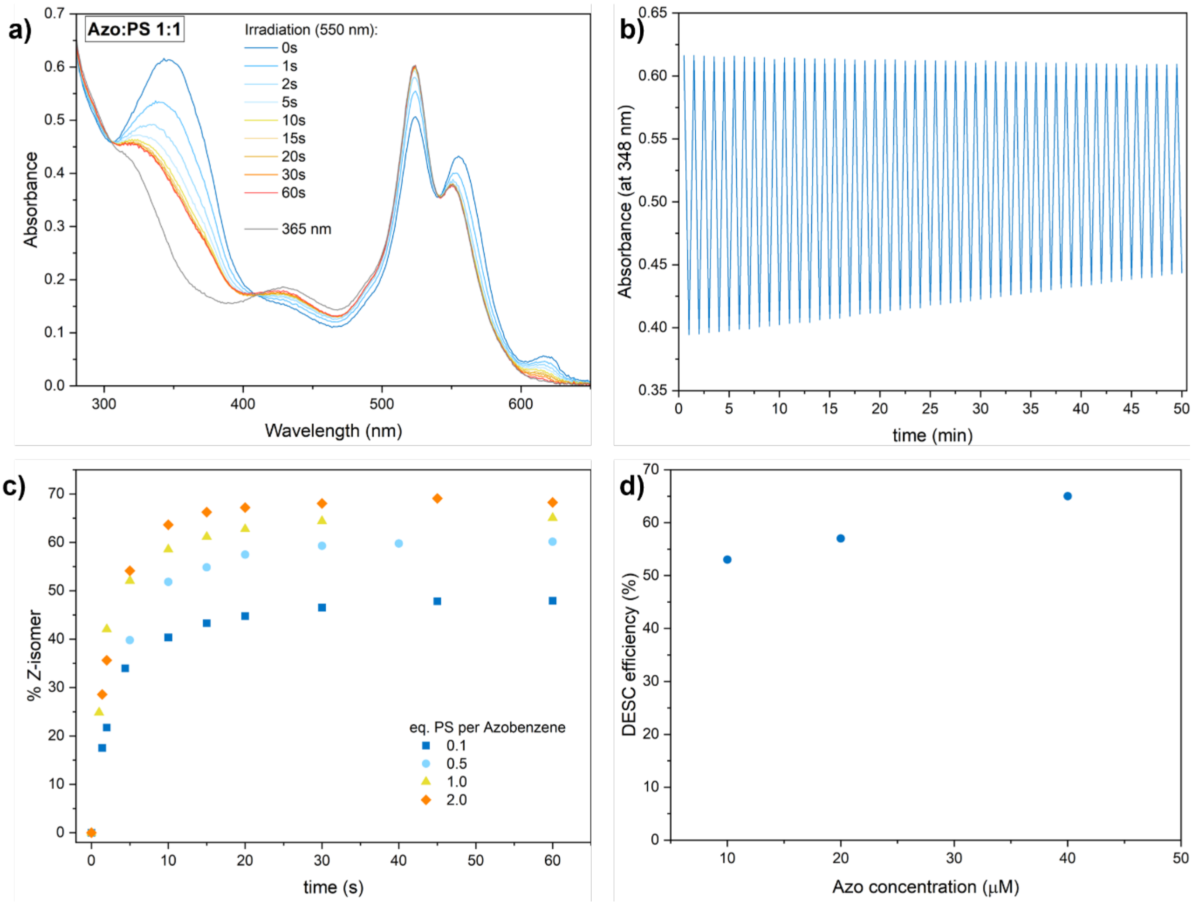


Figure S13: Photophysical analysis of p(DMA$_{99}$-*s*-[DEG-Azo]$_1$) using DESC. a) Absorption spectra of p(DMA$_{99}$-*s*-[DEG-Azo]$_1$) + (**PS**)$_2$⊂**H** (20 µM) solution with azobenzene/PS ratio = 1/1 and subsequent changes upon irradiation with 550 nm yellow light and 365 nm UV light, respectively. b) Trace of the absorbance at 363 nm while performing 100 switching cycles using DESC. c) Progression of *E*-*Z* isomerisation using DESC depending on irradiation time and PS amount. d) Concentration-dependent DESC efficiency.

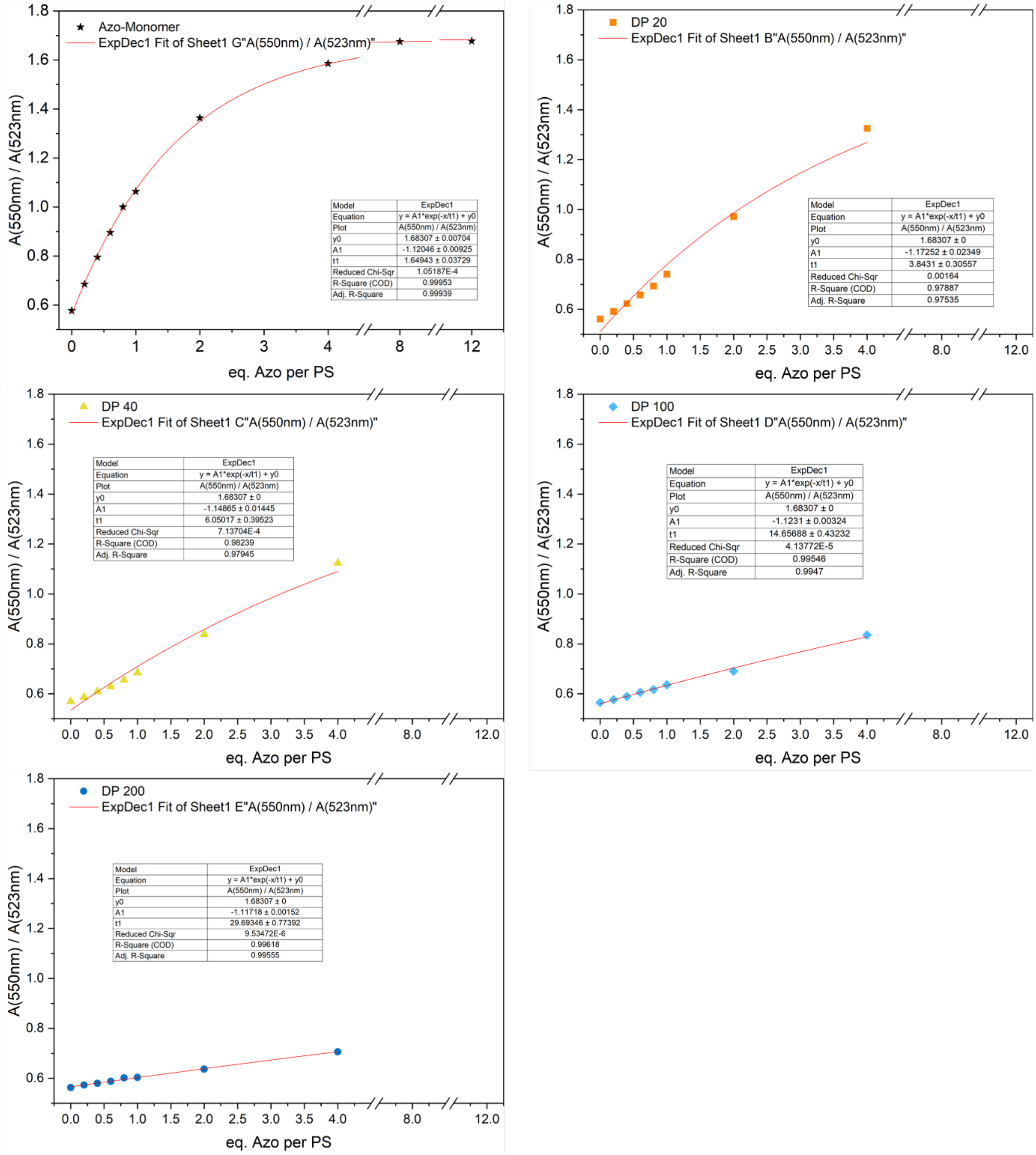


Figure S14: Fitting curves for the progression of A(550 nm) / A(523 nm) during titration of (**PS**)$_2$⊂**H** with different azopolymers of varying degree of polymerisation.

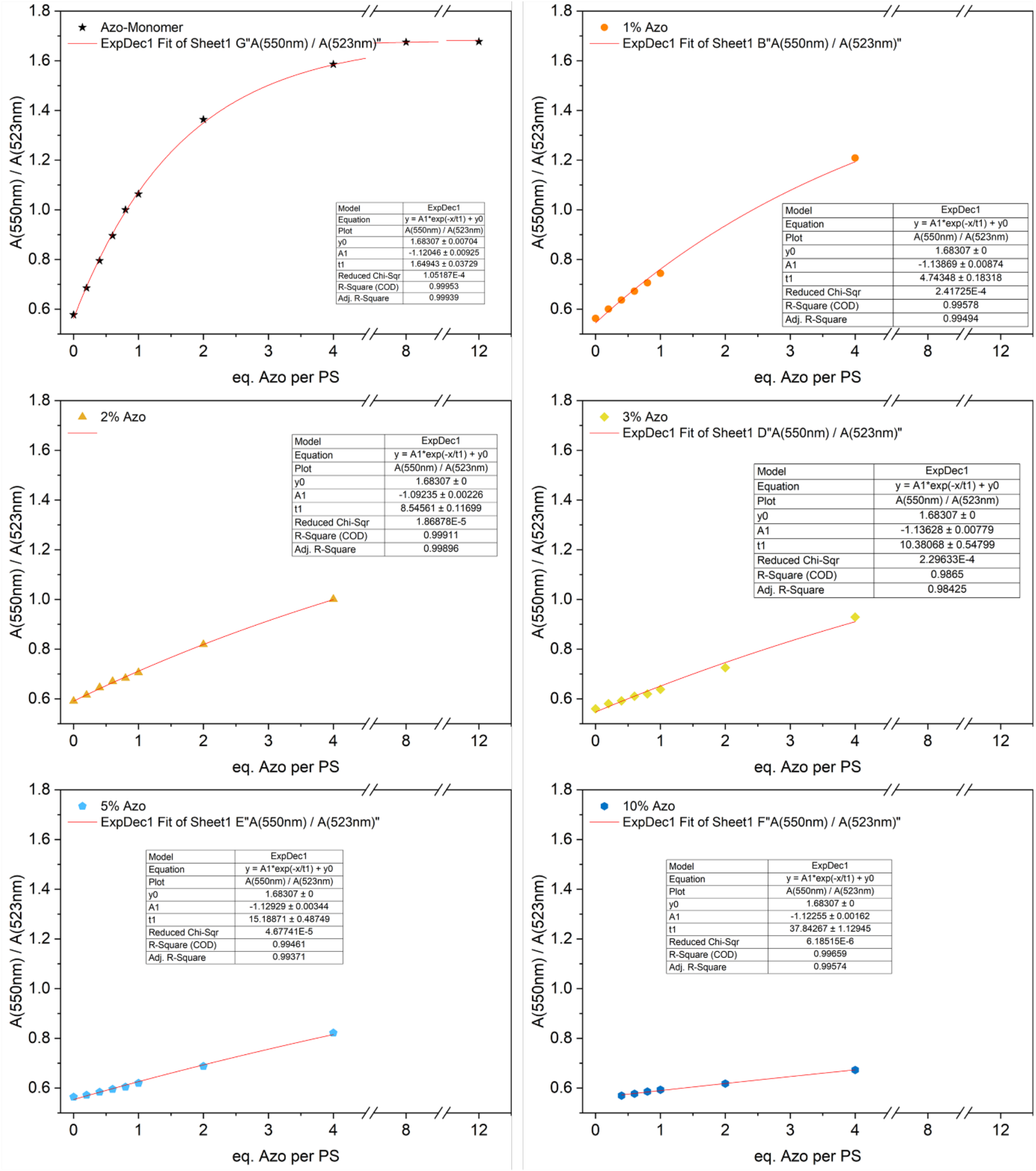


Figure S15: Fitting curves for the progression of A(550 nm) / A(523 nm) during titration of (**PS**)$_2$⊂**H** with different azopolymers with varying functionalisation density.

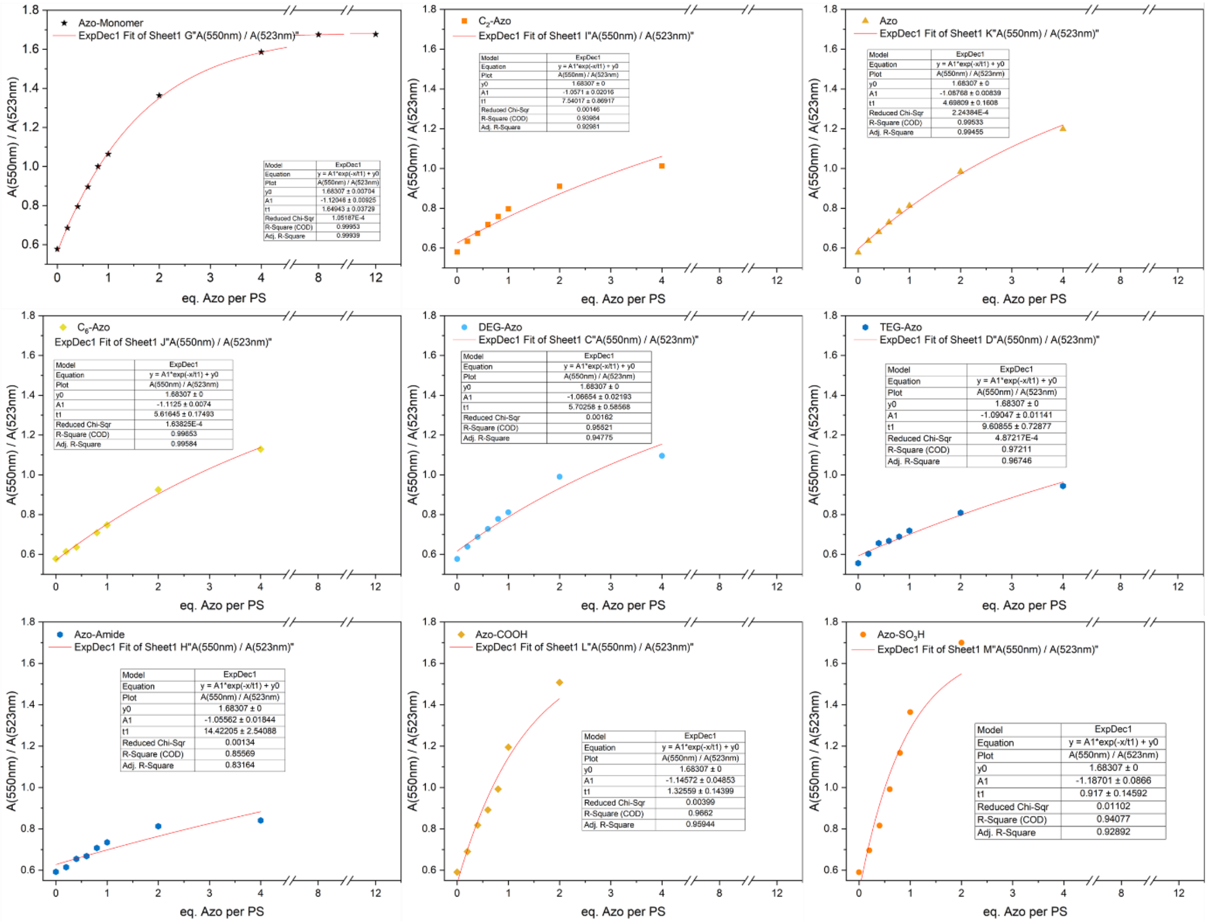

Figure S16: Fitting curves for the progression of A(550 nm) / A(523 nm) during titration of (**PS**)$_2$⊂**H** with azopolymers with varying azobenzene structures.

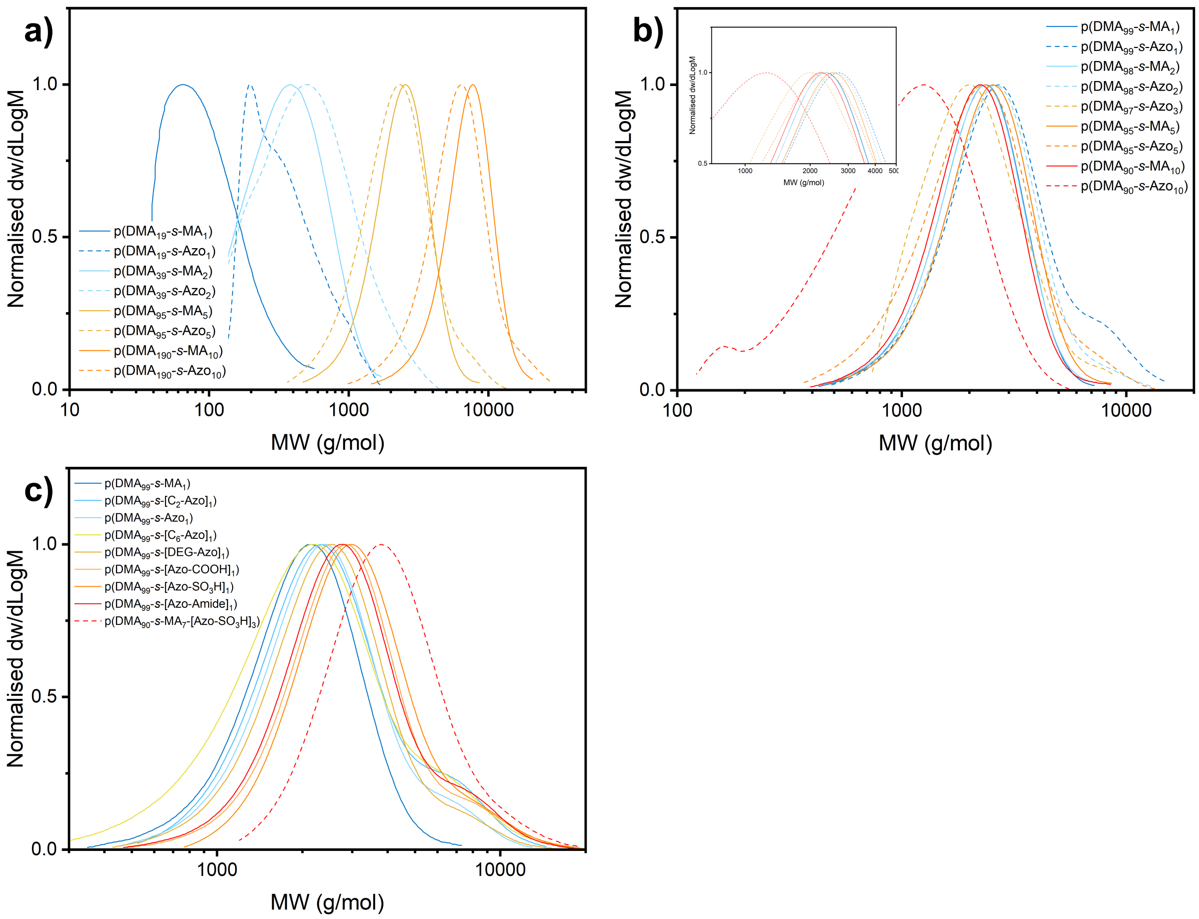


Figure S17: SEC profiles of the synthesised p(DMA-MA) and p(DMA-Azo) polymers with a) varying DP, maintaining functionalisation density at 5%; b) varying functionalisation density, maintaining DP = 100; c) varying azobenzene structure, maintaining DP = 100 and functionalisation density at 1%. Analysis in DMSO + 0.01 M LiBr with DRI detection and poly(sodium styrenesulfonate) calibration.

# 3. Materials and methods

All commercially available chemicals were used without further purification. Chemicals were purchased from Sigma-Aldrich/Merck, TCI Europe, VWR, Fluorochem, Boron Molecular, Acros Organics, Fisher, and BLD Pharma.

For synthesis, solvents of analytical grade were used without further purification, unless stated otherwise. Synthesis under inert conditions was carried out using standard Schlenk techniques with dried solvents and argon as an inert gas.

### Mass spectrometry

All measurements were performed with the JEOL JMS-T100LP AccuTOF LC-plus 4G time-of-flight mass spectrometer using electrospray ionisation. Methanol was used as a solvent for ESI.

### NMR spectroscopy

Nuclear magnetic resonance (NMR) spectra were measured with a 500 MHz JEOL ECZR 500 (126 MHz for $^{13}$C) at room temperature. Chemical shifts (δ) are reported in units of parts per million relative to tetramethylsilane. All $^{1}$H NMR spectra were referenced to proton signals of residual non-deuterated solvents and $^{13}$C spectra were referenced to carbon signals of the solvent. Coupling constants (J) are given in Hertz (Hz). MestReNova (version 15.0.1) was used for the evaluation of the NMR data. The following description was used for the multiplicity of the signal: s = singulet, d = doublet, t = triplet, m = multiplet, b = broad. Signals in the experimental part of this work are given as shown below:

Chemical shift δ [ppm] (multiplicity, coupling constant J [Hz], integral, assignment)

### Size-exclusion chromatography

The size-exclusion chromatography (SEC) experiments were carried out in DMSO with 0.01 M LiBr using Agilent 1260 Infinity II Multi-Detector GPC/SEC System including refractive index detector. Two Agilent 7.5 mm × 300 mm PLgel MIXED-B columns with a 7.5 mm × 50 mm a guard column were used for separation with a flow rate of 0.6 ml/min. The columns were thermoregulated to 60 °C. The injection volume was 100 µl. The columns were calibrated using narrow dispersity polystyrene sulfonate stardards. Agilent OpenLAB CDS ChemStation Edition software was used for instrument control and Agilent GPC/SEC software for data collection and handling.

### Determination of the photostationary distribution of polymeric azobenzenes

The $\mathbf{PSD}_{365nm}$ for p($DMA_{19}$-*s*-$Azo_1$) was determined by $^{1}$H-NMR spectroscopy (Figure S*51*), recording the non-irradiated sample and the same sample after irradiation with 365 nm for 15 minutes. The $\mathbf{PSD}_{550nm}$ of this azopolymer was quantified by comparing absorbance changes at 350 nm upon irradiation with yellow (550 nm) and UV (365 nm) light, referenced to $PSD_{365nm}$ (see equation below).

$$PSD_{550nm} = \frac{\Delta A_{350nm}(yellow\ light)}{\Delta A_{350nn}(UV\ light)} \cdot PSD_{365nm}$$

The other azopolymers were assumed to have a similar $\mathbf{PSD}_{365nm}$, leading to an approximation of the DESC efficiency. The $\mathbf{PSD}_{365nm}$ for p($DMA_{99}$-*s*-[Azo-$SO_3H$]$_1$) was also determined by $^{1}$H NMR spectroscopy.

### UV/vis spectroscopy & irradiation studies

UV/vis absorption spectra were recorded with an Agilent Cary 60 spectrophotometer and an Ocean Optics Qpod 2e Peltier-thermostated cell holder with a temperature accuracy of 0.1 °C. Measurements were conducted at 25 °C.

Photoexcitation was performed using a CoolLED pE-4000 16-LED light source, containing several narrow-band LEDs at different wavelengths. Illumination powers for each wavelength were set according to the following table:

Table S1:Optical power of the LEDs applied in this work

| | |
|---|---|
| 365 nm | 320 mW/cm$^2$ |
| 435 nm | 150 mW/cm$^2$ |
| 550 nm | 330 mW/cm$^2$ |

Quartz fluorescence cuvettes with an optical path of 1.0 cm were used for all measurements. The solutions were stirred with a small stirring bar to ensure mixing during irradiation.

Sample preparation:

*Preparation of inclusion complexes*: A 2 mM stock solution of the host **H** was prepared. The **PS** was encapsulated by adding 5 eq to the **H-**stock solution and stirring overnight. The resulting solution was filtered. Azopolymers were dissolved in water, stirred overnight, and filtered.
Each sample solution was prepared by first adding the (**PS**)$_2$⊂**H** into the cuvette, followed by the “empty” cage (if used) and azopolymer.

**Titrations & data fitting**

Titrations were conducted, maintaining the (**PS**)$_2$⊂**H** concentration at 20 mM and increasing the azopolymer amount. For each datapoint, a new solution was prepared to negate any dilution effects. Saturation curves were fitted in Origin 2023b using the ExpDec1 function. As for the Azo-monomer, nearly complete saturation was obtained; the $y_0$ value was taken and set as a constant for the fitting of the other species, assuming it as the maximum heterodimer population.

The fitting function ExpDec1 is given as follows:

$$y = y_0 + B \cdot exp\left(\frac{-x}{t}\right)$$

with $\alpha$ = 1/t. For normalisation, for each dataset $\beta$ was divided by 0.606, the $\alpha$ -value for the azo-monomer.

Table S2: CMC and mass concentrations of each azopolymer studied by titration at $c_{Azo}$ = 40 µM (“1 eq”).

| Polymer | CMC (mg/mL) | β (mg/mL) at $c_{Azo}$ = 40 µM |
|---|---|---|
| p($DMA_{19}$-*s*-$Azo_1$) | 0.003 | 0.10 |
| p($DMA_{38}$-*s*-$Azo_2$) | 0.008 | 0.10 |
| p($DMA_{95}$-*s*-$Azo_5$) | 0.03 | 0.08 |
| p($DMA_{190}$-*s*-$Azo_{10}$) | 0.08 | 0.08 |
| p($DMA_{99}$-*s*-$Azo_1$) | 0.05 | 0.40 |
| p($DMA_{98}$-*s*-$Azo_2$) | n.d. | 0.22 |
| p($DMA_{97}$-*s*-$Azo_3$) | n.d. | 0.14 |
| p($DMA_{95}$-*s*-$Azo_5$) | 0.03 | 0.08 |
| p($DMA_{90}$-*s*-$Azo_{10}$) | 0.006 | 0.05 |
| p($DMA_{99}$-*s*-$[C_2\text{-}Azo]_1$) | n.d. | 0.43 |
| p($DMA_{99}$-*s*-$Azo_1$) | n.d. | 0.73 |
| p($DMA_{99}$-*s*-$[C_6\text{-}Azo]_1$) | n.d. | 0.62 |
| p($DMA_{99}$-*s*-$[DEG\text{-}Azo]_1$) | n.d. | 0.65 |
| p($DMA_{99}$-*s*-$[TEG\text{-}Azo]_1$) | n.d. | 0.68 |
| p($DMA_{99}$-*s*-$[Azo\text{-}COOH]_1$) | n.d. | 0.44 |
| p($DMA_{99}$-*s*-$[Azo\text{-}SO_3H]_1$) | n.d. | 1.76 |
| p($DMA_{99}$-*s*-$[Azo\text{-}Amide]_1$) | n.d. | 0.48 |
| p($DMA_{90}$-*s*-MA-*s*-$[Azo\text{-}SO_3H]_3$) | n.d. | 0.15 |

**Lifetime studies**

The half-life times of azopolymers were determined by recording the recovery of the absorption at the wavelength of the π-π*-band maximum and performing either a monoexponential fit (Arrhenius)

$$A = A_1 \cdot exp\left(-\frac{x}{t_1}\right) + A_0$$

yielding a half-life time as

$$\tau_{1,2} = ln(2) \cdot t_1$$

or using a stretched-exponential function (Kohlrausch-Williams-Watts)

$$A = A_1 \cdot exp\left(\left(-\frac{x}{t}\right)^b\right) + A_0$$

accounting for the polymeric character of the species

$$\tau_{1/2} = t_1 \cdot (ln(2))^{1/b}$$

Table S 3: Half-life times of Z-azobenzene sidechains of selected azopolymers

| Polymer | $\tau_{1,2}$ (Arrhenius) /h | $\tau_{1,2}$ (stretched exponential) /h |
|---|---|---|
| p($DMA_{19}$-*s*-$Azo_1$) | 21.2 | 43.8 |
| p($DMA_{99}$-s-$[Azo\text{-}SO_3H]_1$) | 77.3 | 69.3 |

# 4. Synthesis of azobenzene derivatives and polymers

The synthesis of the supramolecular host **H** and the photosensitiser **PS** was adopted from the literature.[46,47]
Azobenzene derivatives were synthesised according to Scheme S*1*.

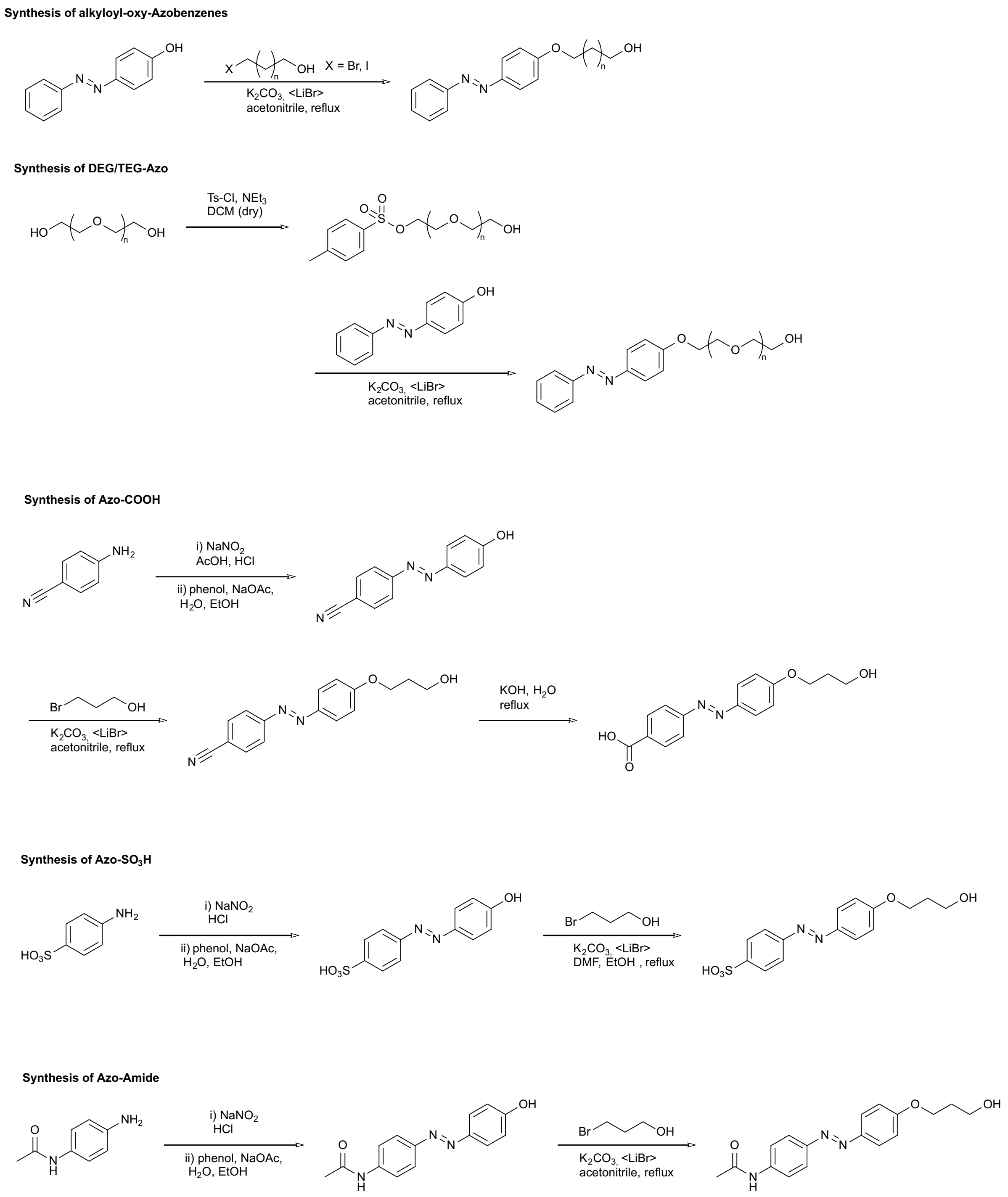


Scheme S1. Synthesis of the azobenzene derivatives used in this study.

**1,3,5-tris(1-imidazolyl) benzene**

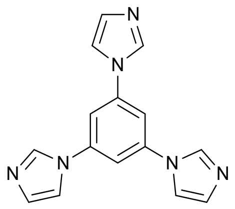


Imidazole (9.220 g, 135.4 mmol, 34 eq), 1,3,5-tribromobenzene (1.260 g, 4.0 mmol, 1.0 eq), $CuSO_4 \cdot 5H_2O$ (0.031 g, 1.24 mmol, 0.31 mmol), and $K_2CO_3$ (2.21 mmol, 16.0 mmol, 4.0 eq) were charged into a dried Schlenk flask under argon and heated at 180 °C overnight. After cooling to room temperature, the mixture was washed with water and filtered. The residue was dissolved in methanol (200 mL) and filtered to remove a brown residue. Upon addition of water, a white solid precipitated, was filtered off, and dried under vacuum to yield the desired compound.

**Molecular formula:** $C_{15}H_{12}N_6$
**Yield:** 83% (0.917 g, 3.32 mmol)
**$^1$H-NMR:** (500 MHz, $CDCl_3$) δ = 8.55 (s, 3H), 8.04 (s, 3H), 7.97 (s, 3H), 7.18 (s, 3H).
**$^{13}$C-NMR:** (126 MHz, DMSO-$d_6$) δ = 139.09, 136.09, 130.15, 118.30, 109.29.

**Supramolecular cage / Host H**

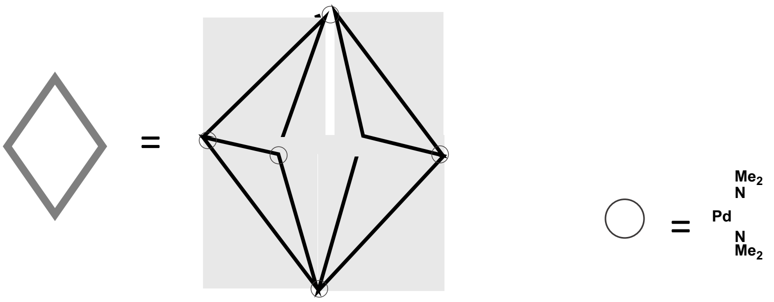


$Pd(NO_3)_2 \cdot 2H_2O$ (0.214 g, 0.804 mmol, 1.6 eq) was dissolved in DMSO (15 mL). *N*,*N*,*N'*,*N'*-tetramethylethylenediamine (TMEDA) (0.093 g, 0.800 mmol, 1.6 eq) was added, and the mixture was heated at 80 °C for 15 minutes. Then, 1,3,5-tris(1-imidazolyl) benzene (0.141 g, 0.508 mmol, 1.0 eq) was added, and the mixture was stirred overnight at 80 °C. Upon cooling to room temperature, the mixture was filtered through cotton, and EtOAc (35 mL) was added, leading to precipitation. The suspension was centrifuged (6500 rpm, 7 min), the yellow suspension was decanted, and the residue was washed with acetone (30 mL). The centrifugation/washing cycle was repeated 3 times. The residue was then dried under vacuum overnight.

**$^1$H NMR:** (500 MHz, $D_2O$) δ = 9.05 (s, 6H), 8.74 (s, 4H), 7.68 (s, 4H), 7.64 (s, 4H), 7.62 (s, 8H), 7.57 (s, 8H), 7.47 (s, 12H), 3.04 (s, 24H), 2.82–2.54 (m, 11H).

**3,5-dimethyl-1*H*-pyrrole-2-carbonyl fluoride**

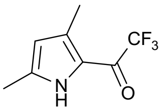


2,4-Dimethylpyrrole (0.924 g, 9.71 mmol, 1.0 eq) was dissolved in DCM (dry, 10 mL) under an argon atmosphere. Trifluoroacetic anhydride (2.243 g, 10.68 mmol, 1.5 eq) was added dropwise at 0 °C (the solution turned from colourless to a dark pink). After 2.5 h, the reaction was quenched with a saturated aqueous $NaHCO_3$ solution, extracted with DCM (3×), and dried over $MgSO_4$. After solvent evaporation, the crude was purified by column chromatography (silica, ethyl acetate / hexanes 4:1) to yield a brown solid.

**Molecular formula:** $C_8H_8F_3NO$
**Yield:** 83% (1.54 g, 8.06 mmol)
**$^1$H NMR:** (500 MHz, $CDCl_3$) δ = 9.26 (b, 1H), 5.97 (s, 1H), 2.36 (s, 3H), 2.32 (s, 3H).
**$^{13}$C NMR:** (126 MHz, $CDCl_3$) δ = 120.81, 118.52, 116.23, 115.07, 113.93, 13.73, 13.50.
**MS (*m/z*):** (ESI, MeOH) Calculated for $C_8H_8F_3NNaO^+$ $[M+Na]^+$: 214.0450; found: 214.0441

**5-trifluoromethyl-1,3,7,8-tetramethyl-4,4-difluoro-4-bora-3a,4a-diaza-s-indacene (BODIPY, PS)**

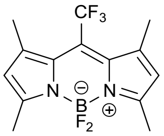


3,5-Dimethyl-1*H*-pyrrole-2-carbonyl fluoride (0.600 g, 3.24 mmol, 1.0 eq) and 2,4-dimethylpyrrole (0.463 g, 4.86 mmol, 1.5 eq) were dissolved in DCM (dry, 10 mL) under an argon atmosphere. $POCl_3$ (0.497 g, 3.24 mmol, 1.0 eq) was added slowly at 0 °C under stirring, and the mixture was allowed to slowly warm to room temperature within 4 h. Boron trifluoride etherate (2.240 g, 15.78 mmol, 4.9 eq) and $NEt_3$ (1.42 g, 14.34 mmol, 4.4 eq) were added and the solution was stirred for 30 min. The reaction was quenched by pouring the mixture into ice-cold water (50 mL), and a diluted HCl solution (10%) was added to adjust the pH to 7. The mixture was extracted with DCM (twice), and the organic layers were dried over $MgSO_4$. The crude product was purified via column chromatography (silica, 0.1→1% EtOAc in hexanes) to obtain **PS** as a golden solid.

**Molecular formula:** $C_8H_8F_3NO$
**Yield:** 15% (0.154 g, 0.486 mmol)
**$^1$H NMR:** (500 MHz, $CDCl_3$) δ = 6.15 (s, 2H), 2.54 (s, 6H), 2.30 (s, 6H).
**$^{13}$C NMR:** (126 MHz, $CDCl_3$) δ = 158.95, 143.26, 124.52, 16.07, 16.01, 15.17, 0.15.
**$^{19}$F NMR:** (471 MHz, $CDCl_3$) δ = -52.66 (t, 3F), -146.00 (q, 2F).

**4-Acrylamidoazobenzene**

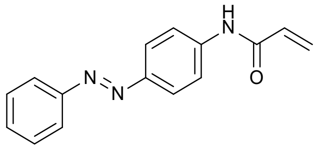

4-Aminoazobenzene (0.707 g, 3.58 mmol, 1.0 eq) was dissolved in DCM (15 mL, dry) under an Ar atmosphere and cooled to 0 °C. Triethylamine (0.472 g, 4.66 mmol, 1.3 eq) was slowly added. Acryloyl chloride (0.390 g, 4.3 mmol, 1.2 eq) was dissolved in dry DCM (0.7 mL) and added dropwise to the solution. The mixture was kept stirring and allowed to slowly warm to room temperature overnight. The reaction was quenched by adding $H_2O$ (10 mL), and the two phases were separated. The aqueous phase was acidified using 1 M HCl and extracted with DCM (2 × 20 mL). After solvent evaporation, the crude product was purified by column chromatography (silica, DCM→DCM/MeOH 97/3) to yield the desired compound.

**Molecular formula:** $C_{15}H_{13}N_3O$
**Yield:** 87% (0.779 g, 3.1 mmol)
**$^1$H-NMR**(500 MHz, $CDCl_3$) δ = 7.94 (d, *J*=8.8 Hz, 2H), 7.90 (d, *J*=7.4 Hz, 2H), 7.77 (d, *J*=8.4 Hz, 2H), 7.51 (t, *J*=7.4 Hz, 2H), 7.46 (t, *J*=7.2 Hz, 1H), 6.49 (dd, *J*=16.8, 1.1 Hz, 1H), 6.29 (dd, *J*=16.8, 10.2 Hz, 1H), 5.83 (dd, *J*=10.3, 1.1 Hz, 1H).
**$^{13}$C NMR** (126 MHz, $CDCl_3$) δ = 163.63, 152.77, 149.30, 140.36, 130.98, 129.23, 128.78, 124.19, 122.90, 120.07.
**MS (*m/z*):** (ESI, MeOH) Calculated for $C_{15}H_{14}N_3O^+$ $[M+H]^+$: 252.1131; found: 252.1139

**4-(Hexyl-1-oxy-6-ol)-azobenzene ($C_6$-Azo)**

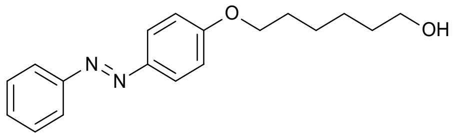

4-(Phenylazo)phenol (0.457 g, 2.31 mmol, 1.0 eq), 6-bromohexan-1-ol (0.554 mmol, 3.06 mmol, 1.3 eq), and $K_2CO_3$ (1.750 g, 12.7 mmol, 5.5 eq) were suspended in acetonitrile (27 mL) and refluxed overnight. The mixture was then cooled to room temperature. The solid was filtered off, and the solvent was evaporated under reduced pressure. The crude product was purified via column chromatography (silica, DCM→DCM/MeOH 95/5).

**Molecular formula:** $C_{18}H_{22}N_2O_2$
**Yield:** quant. (0.691 g, 2.31 mmol)
**$^1$H NMR** (500 MHz, $CDCl_3$) δ = 7.92 (d, *J*=9.0, 2H), 7.88 (d, *J*=7.1, 2H), 7.50 (t, *J*=7.5, 2H), 7.44 (t, *J*=7.3, 1H), 7.00 (d, *J*=9.0, 2H), 4.04 (t, *J*=6.5, 2H), 3.67 (t, *J*=6.6, 2H), 1.90 – 1.77 (m, 2H), 1.62 (dt, *J*=14.2, 6.8, 2H), 1.56 – 1.41 (m, 2H).
**$^{13}$C NMR** (126 MHz, $CDCl_3$) δ = 161.75, 152.86, 146.96, 130.45, 129.15, 124.87, 122.65, 114.79, 68.29, 62.98, 32.77, 29.28, 25.98, 25.66.
**MS (*m/z*):** (ESI, MeOH) Calculated for $C_{18}H_{23}N_2O_2^+$$[M+H]^+$: 299.1754; found: 299.1750

**4-(Propyl-1-oxy-3-ol)-azobenzene (Azo)**

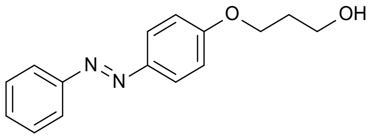


4-(Phenylazo)phenol (0.908 g, 4.58 mmol, 1.0 eq), 3-bromopropan-1-ol (0.966 g, 6.95 mmol, 1.5 eq), and $K_2CO_3$ (3.33 g, 24.1 mmol, 5.3 eq) were suspended in acetonitrile (30 mL) and refluxed overnight. The mixture was then cooled to room temperature. The solid was filtered off, and the solvent was evaporated under reduced pressure. The crude product was purified via column chromatography (silica, DCM→DCM/MeOH 95/5).

**Molecular formula:** $C_{15}H_{16}N_2O_2$
**Yield:** 99% (1.171 g, 4.56 mmol)
**$^1$H-NMR:**(500 MHz, $CDCl_3$) δ = 7.92 (d, *J*=9.0 Hz, 2H), 7.88 (d, *J*=7.2 Hz, 2H), 7.50 (t, *J*=7.5 Hz, 2H), 7.44 (t, *J*=7.4, 1H), 7.02 (d, *J*=9.0 Hz, 2H), 4.21 (t, *J*=6.0 Hz, 2H), 3.90 (t, *J*=5.9 Hz, 2H), 2.09 (p, *J*=6.0 Hz, 2H).
**$^{13}$C NMR:**(126 MHz, $CDCl_3$) δ = 161.40, 152.84, 147.16, 130.54, 129.17, 124.90, 122.69, 114.81, 65.92, 60.29, 32.05.
**MS (*m/z*):** (ESI, MeOH) Calculated for $C_{15}H_{17}N_2O_2^+$ $[M+H]^+$: 257.1285; found: 257.1301

**4-(Ethyl-1-oxy-2-ol)-azobenzene ($C_2$-Azo)**

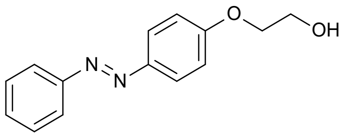


4-(Phenylazo)phenol (0.941 g, 4.65 mmol, 1.0 eq), 2-Iodoethan-1-ol (1.282 g, 7.31 mmol, 1.6 eq), and $K_2CO_3$ (3.271 g, 23.7 mmol, 5.1 eq) were suspended in acetonitrile (30 mL) and refluxed overnight. The mixture was then cooled to room temperature. The solid was filtered off, and the solvent was evaporated under reduced pressure. The crude product was purified via column chromatography (silica, Hex/EtOAc 8/2 → Hex/EtOAc 1/2).

**Molecular formula:** $C_{14}H_{14}N_2O_2$
**Yield:** 49% (0.549 g, 2.27 mmol)
**$^1$H NMR:**(500 MHz, $CDCl_3$) δ = 7.93 (d, *J*=9.0, 2H), 7.88 (d, *J*=7.1, 2H), 7.51 (t, *J*=7.4, 2H), 7.44 (t, *J*=7.3, 1H), 7.04 (d, *J*=9.0, 2H), 4.18 (t, *J*=4.6 Hz, 2H), 4.02 (t, *J*=4.6 Hz, 2H).
**$^{13}$C NMR:** (126 MHz, $CDCl_3$) δ = 161.17, 152.81, 147.35, 130.62, 129.19, 124.93, 122.72, 114.89, 69.59, 61.53.
**MS (*m/z*):** (ESI, MeOH) Calculated for $C_{14}H_{15}N_2O_2^+$ $[M+H]^+$: 243.1128; found: 243.1135

**Diethylene glycol mono(p-toluenesulfonate)**

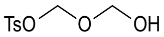


Di(ethylene glycol) (16,436 g, 155 mmol, 10 eq) was dissolved in DCM (30 mL, dry) under an Ar atmosphere and cooled to 0 °C. Triethylamine (2.555 g, 25.2 mmol, 1.6 eq) was added. *p*-Toluenesulfonyl chloride (2.976 g, 15.6 mmol, 1.0 eq) was dissolved in THF (18 mL, dry) and added dropwise to the mixture. The mixture was stirred overnight and allowed to slowly warm to room temperature. DCM (40 mL) was added, and the reaction mixture was washed with water (3 × 40 mL). The combined organic phases were washed with 5% citric acid (2 × 30 mL) and dried over $MgSO_4$. The solvent was evaporated under reduced pressure. The target compound was dried under vacuum and used without further purification ~90% purity).

**Molecular formula:** $C_{11}H_{16}O_6S$
**Yield:** 83% (3.421 g, 13 mmol)
**$^1$H-NMR:** (500 MHz, $CDCl_3$) δ = 7.77 (d, *J*=8.4, 2H), 7.33 (d, *J*=8.5, 2H), 4.18 – 4.15 (m, 2H), 3.68 – 3.62 (m, 4H), 3.50 (dd, *J*=5.1, 4.0, 2H), 2.42 (s, 3H).
**$^{13}$C NMR:** (126 MHz, $CDCl_3$) δ = 144.86, 129.74, 127.67, 77.42, 77.16, 76.90, 72.10, 68.96, 68.39, 61.17, 21.41.

**4-di(ethylene glycol) azobenzene (DEG-Azo)**

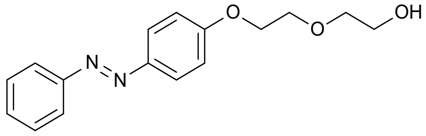


4-(Phenylazo)phenol (0.754 g, 3.80 mmol, 1.0 eq) was dissolved in acetonitrile (50 mL). $K_2CO_3$ (2.733 g, 19.8 mmol, 5.2 eq), di(ethylene glycol) mono(*p*-toluenesulfonate) (1.513 g, 5.23 mmol, 1.4 eq), and a catalytic amount of LiBr were added. The mixture was refluxed for 72 h. Upon cooling down, the solid was filtered off, the solvent was evaporated, and the crude product was purified via column chromatography (silica, Hex/EtOAc 2/1 → EtOAc) to yield the desired compound.

**Molecular formula:** $C_{16}H_{18}N_2O_3$
**Yield:** 81% (0.883 g, 3.08 mmol)
**$^1$H NMR:** (500 MHz, $CDCl_3$) δ = 7.92 (d, *J*=9.0, 2H), 7.88 (d, *J*=7.4, 2H), 7.50 (t, *J*=7.4, 2H), 7.44 (t, *J*=7.3, 1H), 7.03 (d, *J*=9.0, 2H), 4.21 (t, *J*=4.7 Hz, 2H), 3.90 (t, *J*=4.7 Hz, 2H), 3.78 (t, *J*=4.5 Hz, 2H), 3.69i (t, *J*=4.5 Hz, 2H).
**$^{13}$C NMR:** (126 MHz, $CDCl_3$) δ = 161.21, 152.80, 147.24, 130.55, 129.16, 124.86, 122.68, 114.90, 72.75, 69.63, 67.76, 61.87.
**MS (*m/z*):** (ESI, MeOH) Calculated for $C_{16}H_{19}N_2O_3^+$ $[M+H]^+$: 287.1390; found: 287.1464

**Tetraethylene glycol mono(p-toluenesulfonate)**

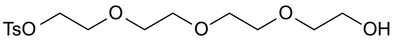


Tetra(ethylene glycol) (7.059 g, 34.5 mmol, 9.4 eq) was dissolved in DCM (11 mL, dry) under an Ar atmosphere and cooled to 0 °C. Triethylamine (0.548 g, 5.41 mmol, 1.5 eq) was slowly added. *p*-Toluenesulfonyl chloride (0.657 g, 3.66 mmol, 1.0 eq) was dissolved in dry THF (5 mL) and added dropwise. The mixture was kept stirring at 0 °C and allowed to slowly warm to room temperature overnight. Upon exposure to air, DCM (15 mL) was added, and the mixture was washed with water (3 × 20 mL). The organic phase was dried over $MgSO_4$, and the solvent was evaporated. The product was dried under vacuum.

**Molecular formula:** $C_{15}H_{24}O_7S$
**Yield:** 92% (1.177 g, 3.38 mmol)
**$^1$H NMR:** (500 MHz, $CDCl_3$) δ = 7.78 (d, *J* = 8.3, 2H), 7.33 (d, *J* = 7.9, 2H), 4.15 (t, *J* = 4.7 Hz, 2H), 3.71–3.57 (m, 14H), 2.43 (s, 3H).
**$^{13}$C NMR:** (126 MHz, $CDCl_3$) δ = 144.96, 132.96, 129.94, 128.08, 72.58, 70.81, 70.72, 70.52, 70.38, 69.36, 68.78, 61.80, 21.76.

**4-tetra(ethylene glycol) azobenzene (TEG-Azo)**

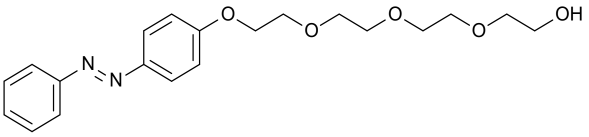


4-(Phenylazo)phenol (0.220 g, 1.10 mmol, 1.0 eq) was dissolved in acetonitrile (25 mL). $K_2CO_3$ (0.766 g, 5.5 mmol, 5.0 eq), tetra(ethylene glycol) mono-(*p*-toluenesulfonate) (0.441 g, 1.20 mmol, 1.1 eq), and a catalytic amount of LiBr were added. The mixture was refluxed for 72 h. Upon cooling down, the solid was filtered off, the solvent was evaporated, and the crude product purified via column chromatography (silica, $Et_2O$ → $Et_2O$/EtOAc 70/30) to yield the desired compound.

**Molecular formula:** $C_{20}H_{26}N_2O_5$
**Yield:** 73% (0.299 g, 0.8 mmol)
**$^1$H NMR:** (500 MHz, $CDCl_3$) δ = 7.91 (d, *J* = 9.0, 2H), 7.87 (d, *J* = 8.1, 2H), 7.49 (t, *J* = 7.7, 2H), 7.44 (d, *J* = 7.3, 1H), 7.03 (d, *J* = 9.0, 2H), 4.23–4.20 (m, 2H), 3.90–3.87 (m, 2H), 3.76–3.58 (m, 12H).
**$^{13}$C NMR:** (126 MHz, $CDCl_3$) δ = 161.25, 152.82, 147.20, 130.51, 129.15, 124.83, 122.67, 114.92, 72.75, 70.91, 70.72, 70.62, 70.32, 69.69, 67.76, 61.81.
**MS (*m/z*):** (ESI, MeOH) Calculated for $C_{20}H_{27}N_2O_5$ $[M+H]^+$: 375.1914; found: 375.1916.

**4-Cyano-4’-hydroxyazobenzene**

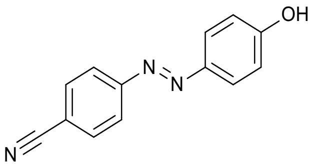

4-Aminobenzonitril (1.212 g, 10.05 mmol, 1.0 eq) was dissolved in conc. acetic acid (16 mL) and conc. HCl (2 mL) was added, leading to the formation of a colourless precipitate. The mixture was cooled to 0 °C. $NaNO_2$ (0.845 g, 11.9 mmol, 1.2 eq) was dissolved in a minimum amount of $H_2O$ and added dropwise to the aminobenzonitril, yielding a yellow solution. The mixture was kept stirring at 0 °C for 30 minutes. Phenol (1.068 g, 11.35 mmol, 1.13 eq) and sodium acetate (3.143 g, 38.4 mmol, 3.8 eq) were dissolved in $H_2O$ (6 mL) and EtOH (10 mL). The diazonium salt was added to the phenolic solution and kept stirring at 0 °C for 3h, then allowed to warm to room temperature. After the addition of $H_2O$ (50 mL), the solution was extracted with DCM (3 x 50 mL). The combined org. phases were dried over $MgSO_4$ and the solvent evaporated under reduced pressure. The crude product was purified by column chromatography (silica, DCM → DCM/ MeOH 96/4) to yield the target compound.

**Molecular formula:** $C_{13}H_9N_3O$
**Yield:** 97% (2.168 g, 9.71 mmol)
**$^1$H NMR:** (500 MHz, $CDCl_3$) δ = 7.94 (d, *J*=8.8, 2H), 7.92 (d, *J*=8.9, 2H), 7.80 (d, *J*=8.8, 2H), 6.97 (d, *J*=8.9, 2H), 5.48 (s, 1H).
**$^{13}$C NMR:** (126 MHz, $CDCl_3$) δ = 133.33, 125.83, 123.25, 116.15.

**4-Cyano-4’-(propyl-1-oxy-3-ol) azobenzene**

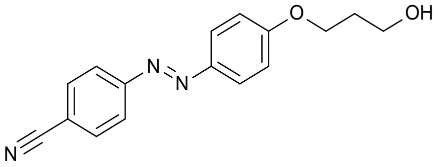

4-Cyano-4’-hydroxyazobenzene (2.16 g, 9.68 mmol, 1.0 eq) and 3-bromopropan-1-ol (1.881 g, 12.45 mmol, 1.3 eq) were dissolved in acetonitrile (50 mL). $K_2CO_3$ (6.824 g, 49.4 mmol, 5.1 eq) and a catalytic amount of LiBr were added, and the mixture was refluxed overnight. After cooling to room temperature, the solid was filtered off, and the filter cake was washed with acetone (200 mL). The organic phases were combined, and the solvent was evaporated under reduced pressure. The crude product was purified via column chromatography (silica, DCM → DCM/MeOH 95/5).

**Molecular formula:** $C_{16}H_{15}N_3O_2$
**Yield:** 97% (2.642 g, 9.4 mmol)
**$^1$H NMR** (500 MHz, DMSO-$d_6$) δ = 8.05 (d, *J*=8.7, 2H), 7.96 (d, *J*=8.8, 2H), 7.94 (d, *J*=9.0, 2H), 7.16 (d, *J*=9.0, 2H), 4.17 (t, *J*=6.3, 2H), 3.57 (t, *J*=6.3, 2H), 1.90 (p, *J*=6.2, 2H).
**$^{13}$C NMR:** (126 MHz, DMSO-$d_6$) δ = 162.51, 154.20, 146.06, 133.83, 125.37, 122.94, 118.58, 115.26, 112.55, 65.29, 57.13, 31.97.

**4-Carboxy-4'-(propyl-1-oxy-3-ol) azobenzene (Azo-COOH)**

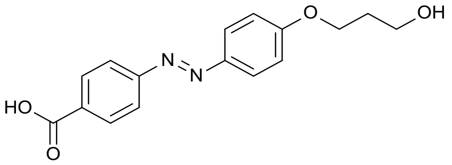


4-Cyano-4'-(propyl-1-oxy-3-ol) azobenzene (2.640 g, 9.40 mmol, 1.0 eq) and KOH (2.483 g, 44.3 mmol, 4.7 eq) were added to $H_2O$ (30 mL), and the mixture was refluxed for 72 h. After cooling to room temperature, the mixture was neutralised with HCl (1 M) to precipitate the product, which was filtered off and dried under vacuum.

**Molecular formula:** $C_{16}H_{16}N_2O_4$
**Yield:** 92% (2.596 g, 8.64 mmol)
**$^1$H NMR:** (500 MHz, DMSO-$d_6$) δ = 8.07 (d, *J*=8.4, 2H), 7.91 (d, *J*=8.9, 2H), 7.85 (d, *J*=8.5, 2H), 7.14 (d, *J*=9.0, 2H), 4.16 (t, *J*=6.4, 2H), 3.57 (t, *J*=6.2, 2H), 1.90 (p, *J*=6.3, 2H).
**$^{13}$C NMR:** (126 MHz, DMSO-$d_6$) δ = 146.15, 130.35, 124.88, 121.89, 115.13, 65.19, 57.16, 32.01.
**MS (*m/z*):** (ESI, MeOH) Calculated for $C_{16}H_{15}N_2O_4^-$ $[M-H]^-$: 299.1037; found: 299.1132

**4-(4'-Hydroxy) azobenzene sulfonic acid**

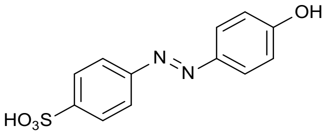


Sulfanilic acid (1.770 g, 10.12 mmol, 1.0 eq) was dispersed in AcOH (16 mL), HCl (2 mL) was added, and the mixture was cooled to 0 °C. $NaNO_2$ (0.874 g, 12.29 mmol, 1.2 eq) was dissolved in a minimum amount of $H_2O$ and added dropwise to the sulfanilic acid solution, and the mixture was stirred for 40 minutes.
Phenol (1.100 g, 11.7 mmol, 1.2 eq) and sodium acetate (3.255 g, 39.7 mmol, 3.9 eq) were dissolved in $H_2O$ (7 mL) and EtOH (10 mL), and the solution was slowly added to the diazonium salt. The mixture was stirred at 0 °C for 1 h, then allowed to warm to room temperature, and stirred overnight.

**Molecular formula:** $C_{12}H_{10}N_2O_4$
**Yield:** 86% (2.422 g, 8.70 mmol)
**$^1$H NMR:** (500 MHz, DMSO-$d_6$) δ = 10.35 (s, 1H), 7.82 (d, *J* = 8.8, 2H), 7.78–7.73 (m, 4H), 6.95 (d, *J* = 8.9, 2H).
**$^{13}$C NMR:** (126 MHz, DMSO-$d_6$) δ = 161.08, 151.76, 149.98, 145.26, 126.66, 124.98, 121.63, 115.99.

**4-(4'- propyl-1-oxy-3-ol) azobenzene sulfonic acid (Azo-$SO_3$H)**

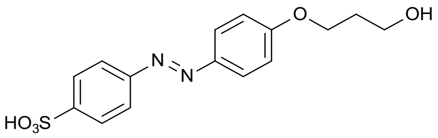


4-(4'-Hydroxy) azobenzene sulfonic acid (1.020 g, 3.67 mmol, 1.0 eq), 3-bromopropan-1-ol (4.45 g, 32.0 mmol, 8.7 eq) and $K_2CO_3$ (2.746 g, 19.9 mmol, 5.4 eq) were dissolved in DMF (50 mL) and EtOH (10 mL) and a catalytic amount of LiBr was added. The mixture was refluxed at 100 °C for 72h. The mixture was then cooled to room temperature and the solvent evaporated. The obtained solid was recrystallised from EtOH/$H_2O$. The crystals were filtered off, washed with icecold water and EtOH and dried under vacuum.

**Molecular formula:** $C_{15}H_{16}N_2O_5S$
**Yield:** 41% (0.503 g, 1.50 mmol)
**$^1$H NMR:** (500 MHz, DMSO-*d*6) δ = 7.90 (d, *J*=9.1, 2H), 7.78 (q, *J*=8.4, 4H), 7.13 (d, *J*=9.1, 2H), 4.15 (t, *J*=6.3, 1H), 3.57 (t, *J*=6.3, 2H), 1.90 (p, *J*=6.3, 2H).
**$^{13}$C NMR:** (126 MHz, DMSO-*d*6) δ = 161.64, 151.68, 150.26, 146.08, 126.70, 124.73, 121.80, 115.08, 65.13, 57.18, 32.03.
**MS (*m/z*):** (ESI, MeOH) Calculated $C_{15}H_{15}N_2O_5S^-$ [M-H]$^-$: 335.0707; found: 335.0821

**4-acetylamido-4'-hydroxyazobenzene**

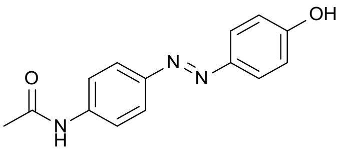


4-Aminoacetoanilide (2.081 g, 13.86 mmol, 1.0 eq) was dissolved in $H_2O$ (8.3 mL) and HCl (12 M, 2.3 mL) and cooled to 0 °C. $NaNO_2$ (1.267 g, 18.36 mmol, 1.3 eq) was dissolved in water (7 mL) and added dropwise. The mixture was kept stirring for 90 minutes at 0 °C. Phenol (1.691 g, 17.97 mmol, 1.3 eq) was dissolved in an aqueous NaOH solution (1.625 g, 40.6 mmol, in 10 mL) and cooled to 0 °C. The diazonium salt was added dropwise, causing a colour change from yellow to brown and the formation of a precipitate. The mixture was stirred overnight and then allowed to slowly warm to room temperature.
The solution was neutralised and extracted with ethyl acetate (3 × 80 mL). After washing with water, the solvent was evaporated.

**Molecular formula:** $C_{14}H_{13}N_3O_2$
**Yield:** 64% (3.01 g, 11.75 mmol)
**$^1$H NMR:** (500 MHz, DMSO-*d*6) δ = 10.25 (s, 1H), 10.24 (s, 1H), 7.83–7.74 (m, 6H), 6.93 (d, *J* = 8.8, 2H), 2.09 (s, 3H).
**$^{13}$C NMR:** (126 MHz, DMSO-*d*6) δ = 168.72, 160.54, 147.58, 145.31, 141.54, 124.55, 123.10, 119.15, 115.92, 24.20.
**MS (*m/z*):** (ESI, MeOH) Calculated for $C_{14}H_{13}N_3NaO_2^+$ [M+Na]$^+$: 278.0894; found: 278.0917.

**4-(4’- propyl-1-oxy-3-ol) azobenzene acetylamide (Azo-Amide)**

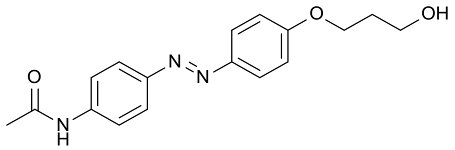

4-acetylamido-4'-hydroxyazobenzene (1.067 g, 4.18 mmol, 1.0 eq), 3-bromopropan-1-ol (0.965 g, 6.45 mmol, 1.5 eq), and $K_2CO_3$ (2.910 g, 21 mmol, 5 eq) were dissolved in acetonitrile, and a catalytic amount of LiBr was added. The mixture was refluxed for 72 h. The mixture was then cooled to room temperature and the solvent was evaporated. The crude was purified by column chromatography (silica, EtOAc/Hex: 10/90→40/60) to yield the target compound with a small amount of unreacted 3-bromopropan-1-ol.

**Molecular formula:** $C_{17}H_{19}N_3O_3$
**Yield:** 83% (1.092 g, 3.48 mmol)
**$^1$H NMR:** (500 MHz, $CDCl_3$) δ = 10.26 (s, 1H), 7.92–7.71 (m, 6H), 7.11 (d, *J* = 9.1, 2H), 4.56 (t, *J* = 5.2, 2H), 3.58 (td, *J* = 6.2, 5.1, 2H), 2.09 (s, 3H), 1.89 (p, *J* = 6.3, 2H).
**$^{13}$C NMR:** (126 MHz, $CDCl_3$) δ = 168.75, 161.13, 154.72, 147.48, 146.13, 141.80, 124.28, 123.27, 119.11, 114.99, 65.07, 57.18, 32.03.
**MS (*m/z*):** (ESI, MeOH) Calculated for $C_{17}H_{19}N_3NaO_3$ $[M+Na]^+$: 336.1319; found: 336.1326.

**Exemplary procedure for the synthesis of linear polymers made by RAFT polymerisation (here: p($DMA_{95}$-*s*-$MA_5$))**

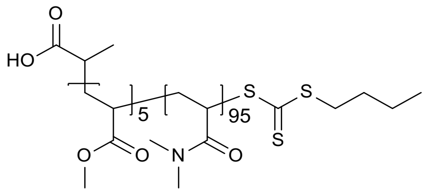

The chain transfer agent (2-(butylthiocarbonothioylthio)propanoic acid) (26.4 mg, 0.105 mmol, 1.0 eq) was placed in a 7 ml vial, and *N*,*N*-dimethylacrylamide (0.998 g, 9.97 mmol, 95 eq) and methyl acrylate (45.7 mg, 0.526 mmol, 5.0 eq) were added. $H_2O$ (1.691 mL) and dioxane (0.728 mL) were added to afford a reaction mixture with the total monomer concentration of ~3 mol/L. The radical initiator 2,2'-azobis[2-(2-imidazolin-2-yl)propane]dihydrochloride (0.68 mg, 0.0021 mmol, 0.02 eq; 100 mg/mL solution in $H_2O$, 6.8 µL taken) was added to the mixture, and the solution was deoxygenated by gently bubbling argon for 10 minutes. Then, the mixture was immersed in a preheated oil bath (70 °C) to start the polymerisation. After 2.5 h, the mixture was exposed to air and cooled in an ice bath to stop the reaction. Upon the addition of acetone (10 mL) to facilitate evaporation, the solvent was removed under reduced pressure, and the polymer was redissolved in acetone (5 mL). The polymer was then precipitated into diethyl ether (45 mL), centrifuged at 6500 rpm for 7 minutes, and the supernatant was decanted. The polymer was redissolved in acetone, precipitated, and centrifuged twice more. Finally, the polymer was dried under vacuum overnight.

Given below are the peaks in focus for the characterization of the polymer:

**$^1$H NMR:** (500 MHz, $CDCl_3$) δ = 3.70–3.51 (m, O-C$H_3$, acrylate), 3.13–2.73 (m, 6H, N-C$H_3$, acrylamide), 2.68–2.28 (m, 2H), 1.92–1.06 (m, 11H), 0.88 (m, 3H, CTA-C$H_3$).

**Exemplary procedure for the post-modification of linear polymers (here: p($DMA_{95}$-*s*-$Azo_5$) with $C_3$Azo)**

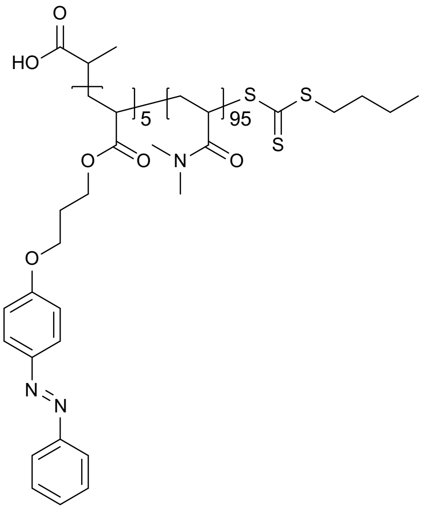

p($DMA_{95}$-*s*-$MA_5$) (163 mg, 0.0776 mmol [Acrylate moieties], 1.0 eq), 4-(propyl-1-oxy-3-ol)-azobenzene (198 mg, 0.776 mmol, 10 eq), and 1,5,7-triazabicyclo[4.4.0]dec-5-ene (7 mg, 0.0503 mmol, 65 mol%) were charged into a two-neck round-bottom flask equipped with a reflux condenser and a gas bubbler under argon atmosphere. Dichlorobenzene (5 mL, dry) was added, and the mixture was refluxed at 140 °C for 3 days. Upon cooling down and exposure to air, the polymer was precipitated into diethyl ether (45 mL) and centrifuged at 6500 rpm for 7 minutes. The supernatant was decanted, and the

polymer redissolved in acetone (5 mL) and precipitated and centrifuged two more times. Finally, the polymer was dried under vacuum overnight.

*For the modification using azobenzenes with hydrophilic substituents, the purification was adapted:*

**Azo-COOH**: Upon cooling down, the mixture was precipitated into diethyl ether (45 mL) and centrifuged at 6500 rpm for 7 minutes. The supernatant was decanted, and acetone (40 mL) was added to the residue (dispersed by vortexing). The suspension was centrifuged (8000 rpm, 7 min) and the supernatant decanted and evaporated to reclaim the polymer (and some remaining Azo-COOH nucleophile). The polymer dissolution/dispersion in acetone / centrifugation / supernatant decantation / solvent evaporation cycle was repeated 3 more times to yield the purified polymer, which was dried under vacuum overnight.

**Azo-Amide**: In general, the purification procedure was kept the same as in the exemplary procedure; however, the precipitation–centrifugation cycle was conducted 10 times to remove any excess Azo-Amide nucleophile.

**Azo-$SO_3H$**: Upon cooling down, the mixture was poured into acetone (30 mL) and centrifuged at 6500 rpm for 7 minutes. The supernatant (containing the polymer) was decanted, and the solvent evaporated under reduced pressure. This procedure was repeated two more times. Then, the polymer solution was precipitated into diethyl ether (45 mL) and centrifuged at 6500 rpm for 7 min. The supernatant was decanted, the residue redissolved in acetone, and again precipitated into diethyl ether, centrifuged, and the supernatant decanted. The polymer was then dialysed against ethanol for 2 days (tube pore size: 3.5 kDa, solvent exchanged twice daily).

Given below are the peaks in focus for the characterisation of the polymer:

**$^1$H NMR:** (500 MHz, $CDCl_3$) δ = 7.91–7.82 (m, 4H, Azo), 7.51–7.39 (m, 3H, Azo), 7.05–6.96 (m, 2H, Azo), 3.17–2.73 (m, 6H, N-C$H_3$, acrylamide).

## 5. Supplementary references

---

[46] M. Canton, A. B. Grommet, L. Pesce, J. Gemen, S. Li, Y. Diskin-Posner, A. Credi, G. M. Pavan, J. Andréasson, R. Klajn, J Am Chem Soc 2020, 142, 14557–14565.

[47] S. Choi, J. Bouffard, Y. Kim, Chem. Sci. 2014, 5, 751–755.

# 6. NMR spectra and SEC traces

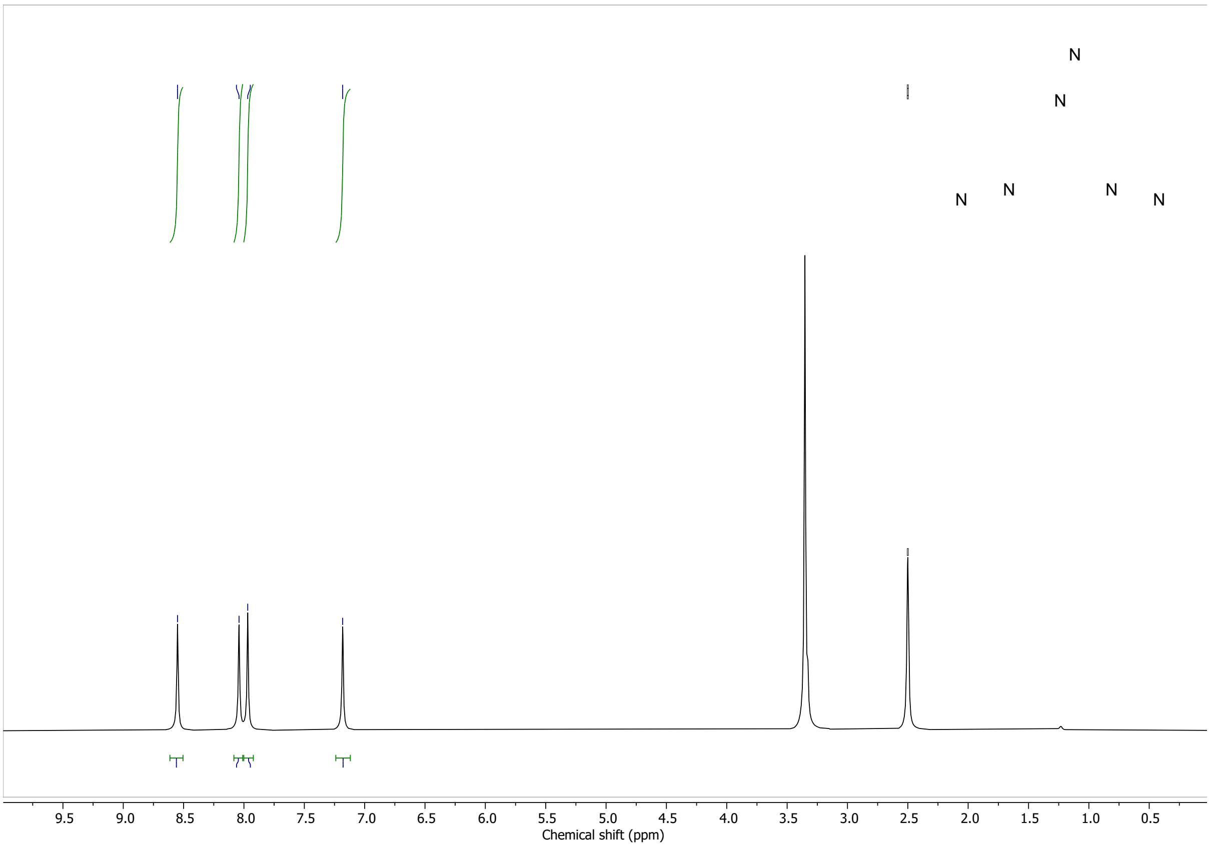


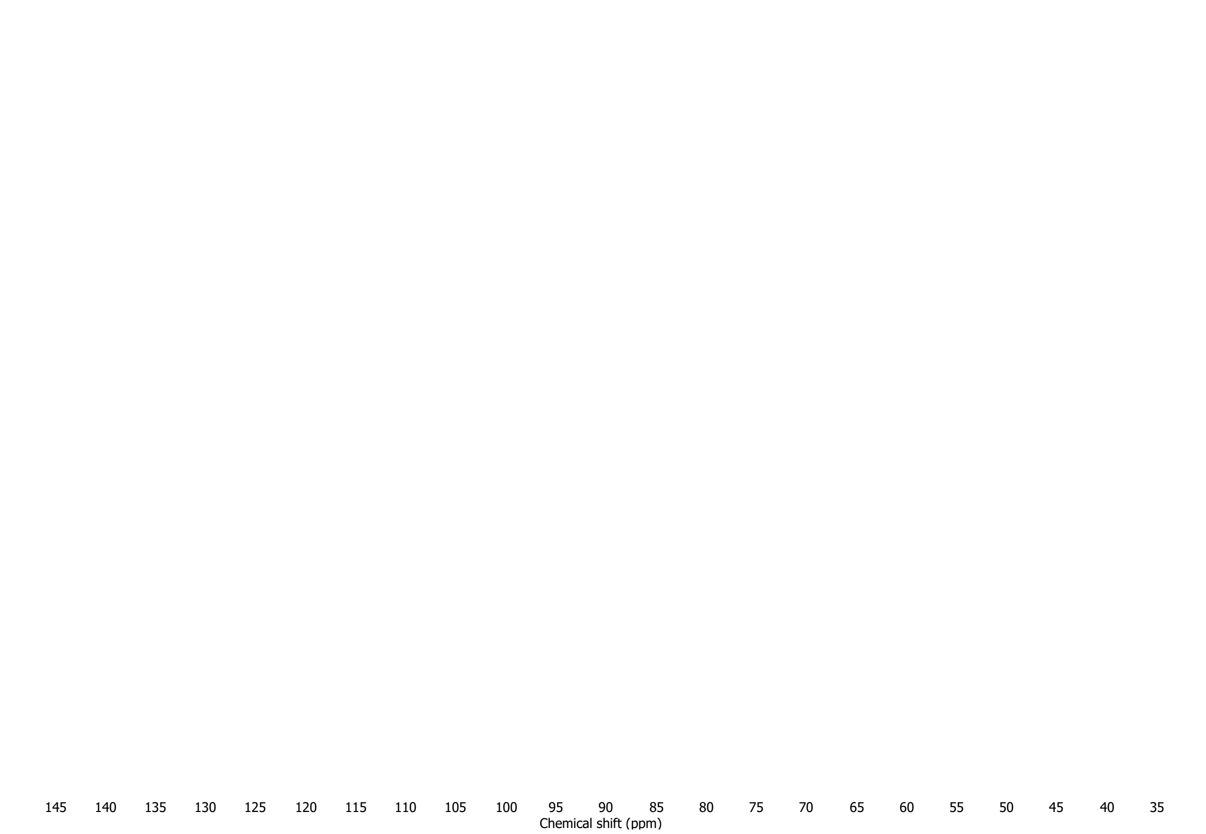


Figure S18: $^{1}H$ and $^{13}C$ NMR spectra of 1,3,5-tris(1-imidazolyl)benzene in DMSO-$d_6$.

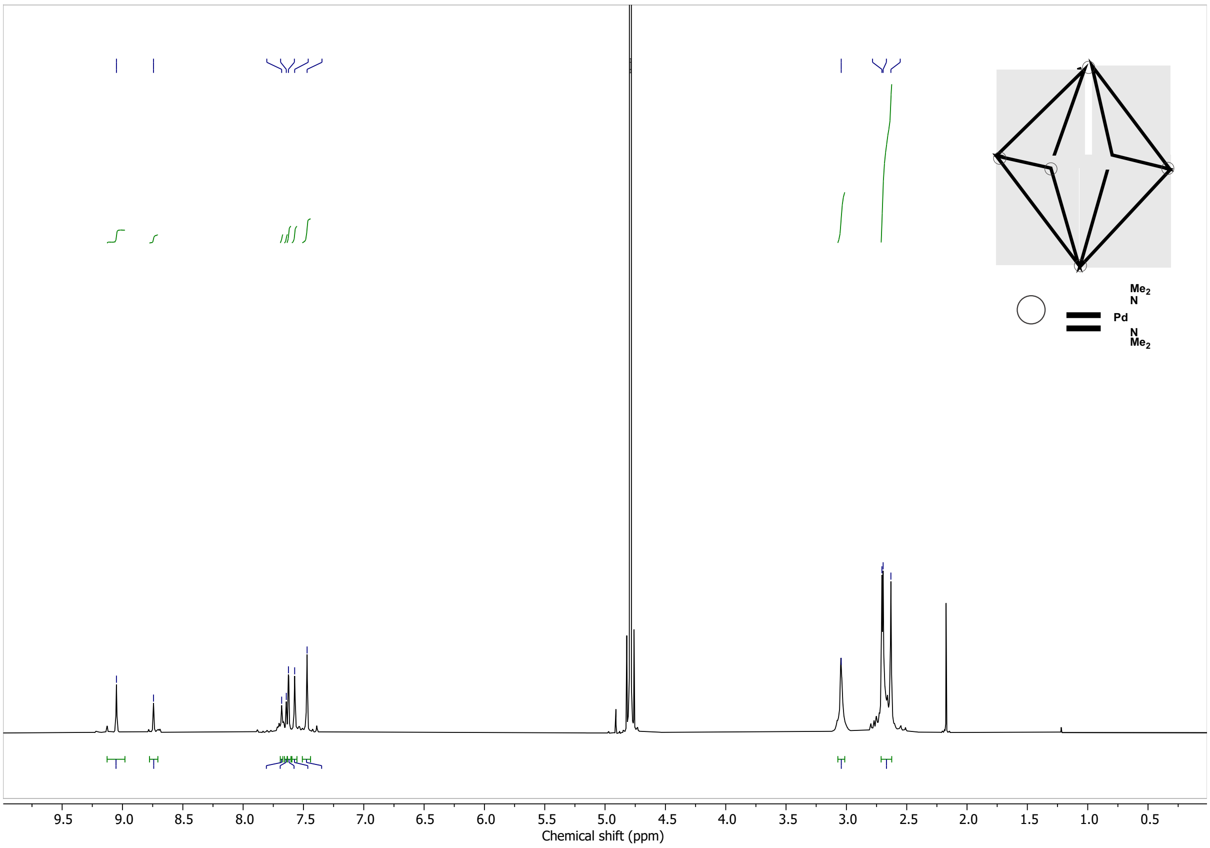


Figure S19: $^{1}H$ NMR spectrum of the supramolecular host **H** in $D_2O$.

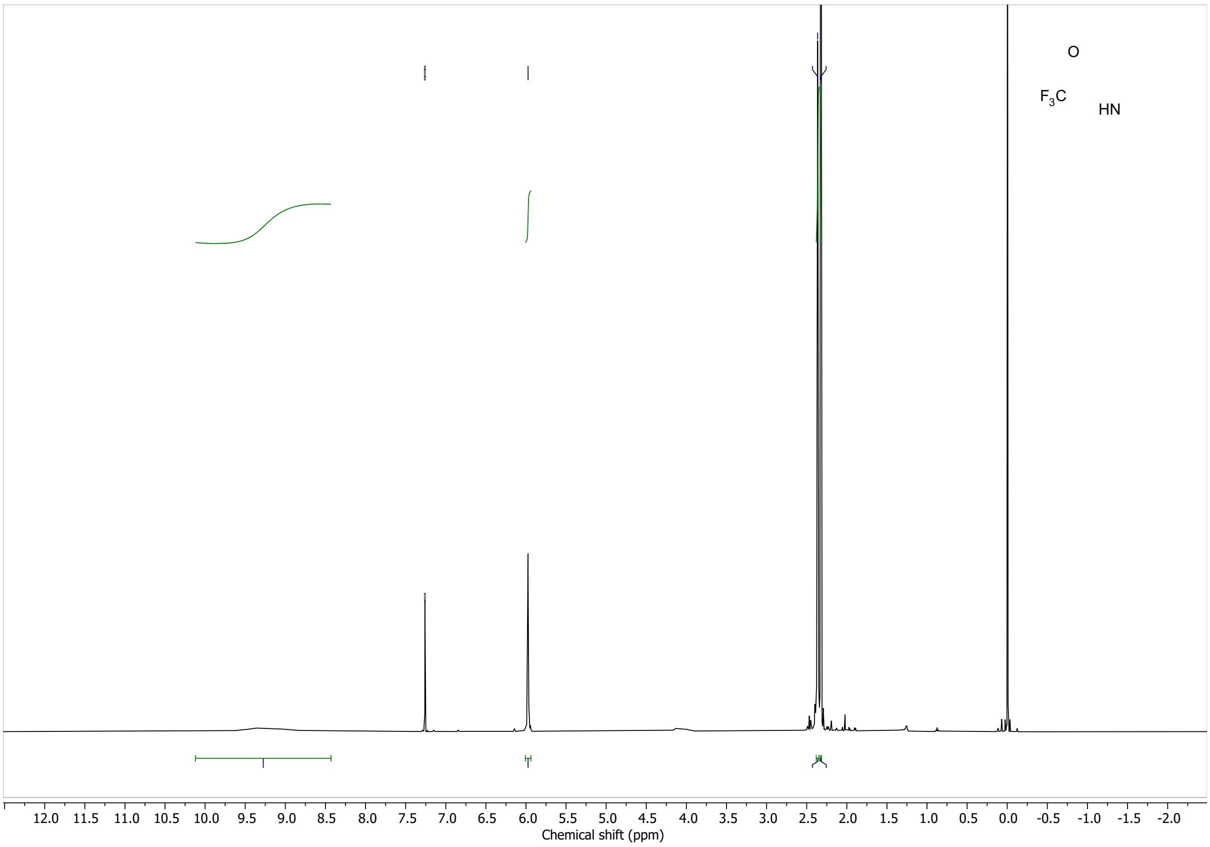


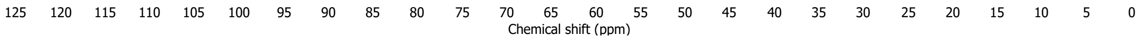


Figure S 20: $^{1}H$ and $^{13}C$ NMR spectra of 3,5-dimethyl-1*H*-pyrrole-2-carbonyl fluoride in $CDCl_3$.

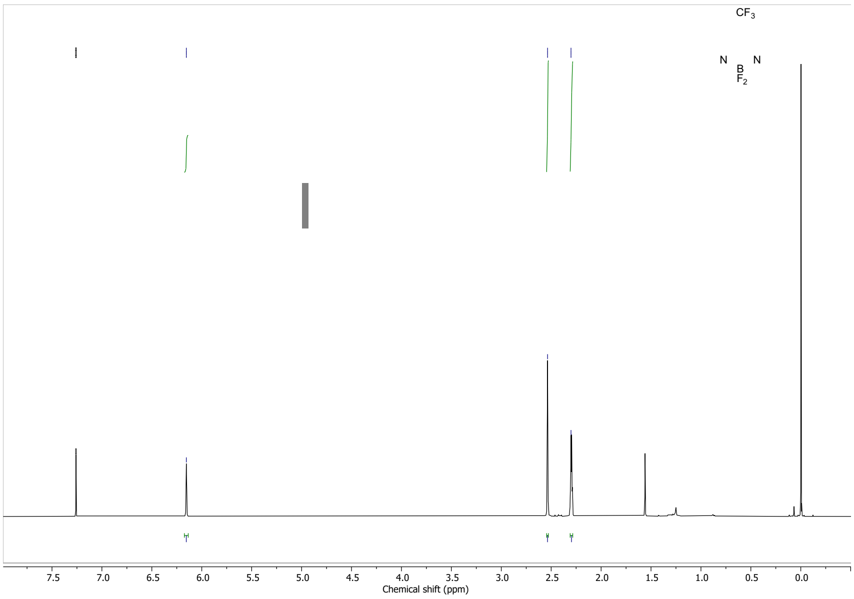


13C NMR (126 MHz, CHLOROFORM-D) δ = 158.95, 143.26, 124.52, 16.07, 16.01, 15.17, 0.15.

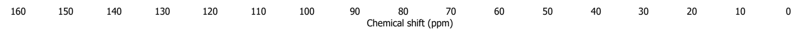


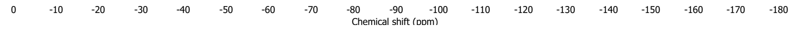


Figure S 21: $^{1}H$, $^{13}C$, and $^{19}F$ NMR spectra of the BODIPY photosensitiser (**PS**) in $CDCl_3$.

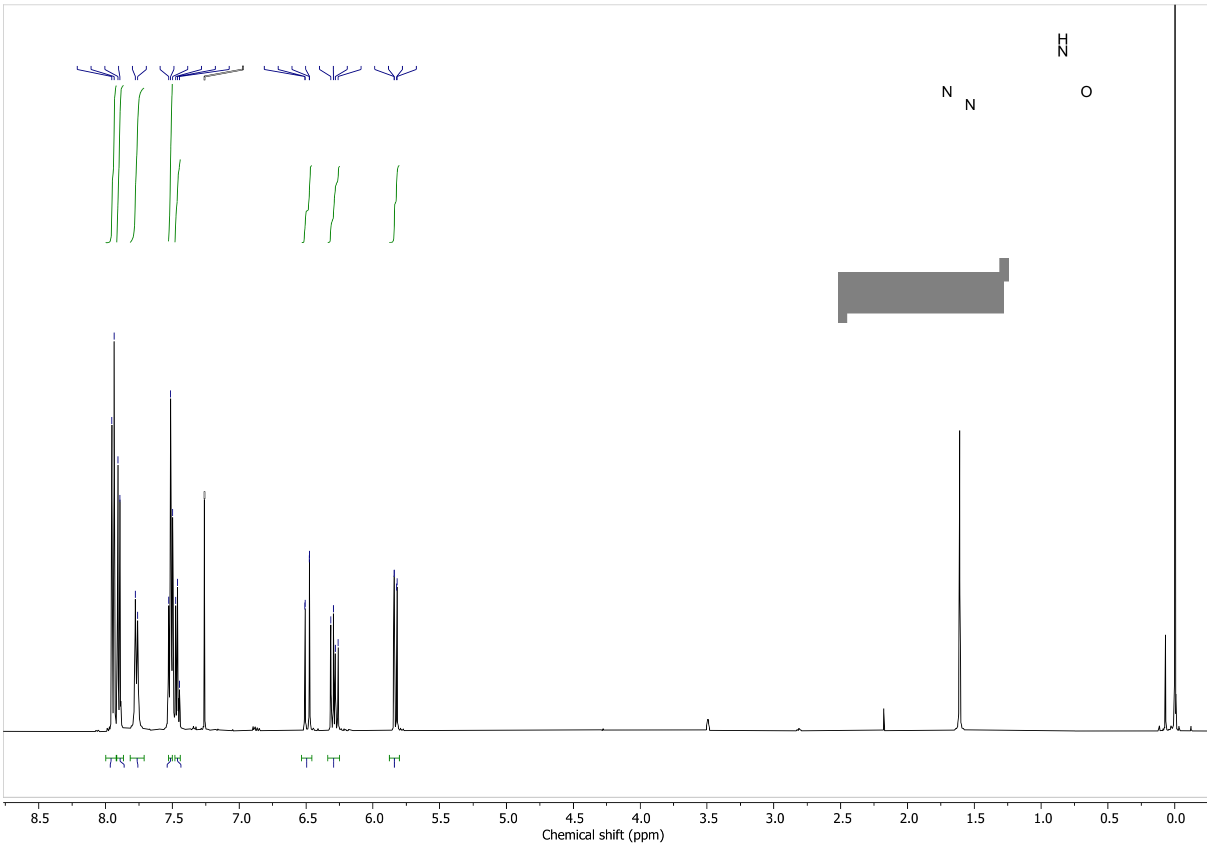


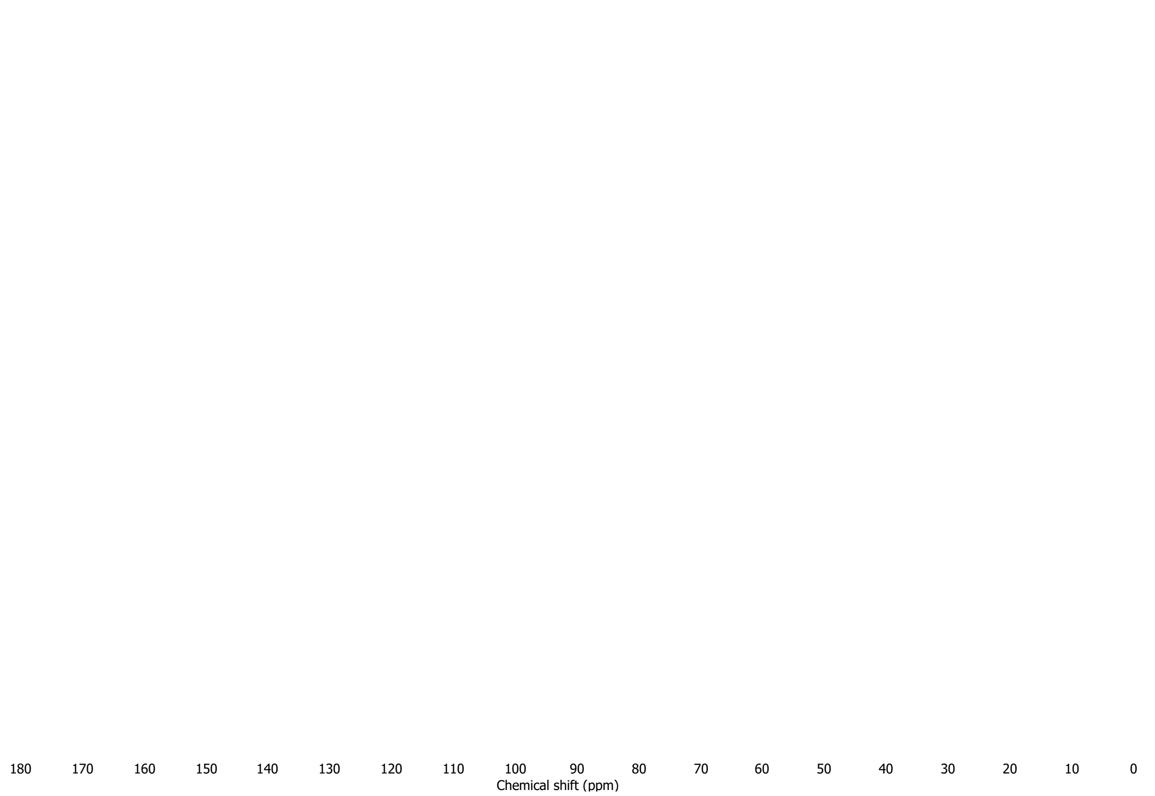


Figure S22: $^{1}H$ and $^{13}C$ NMR spectra of 4-acrylamidoazobenzene in $CDCl_3$.

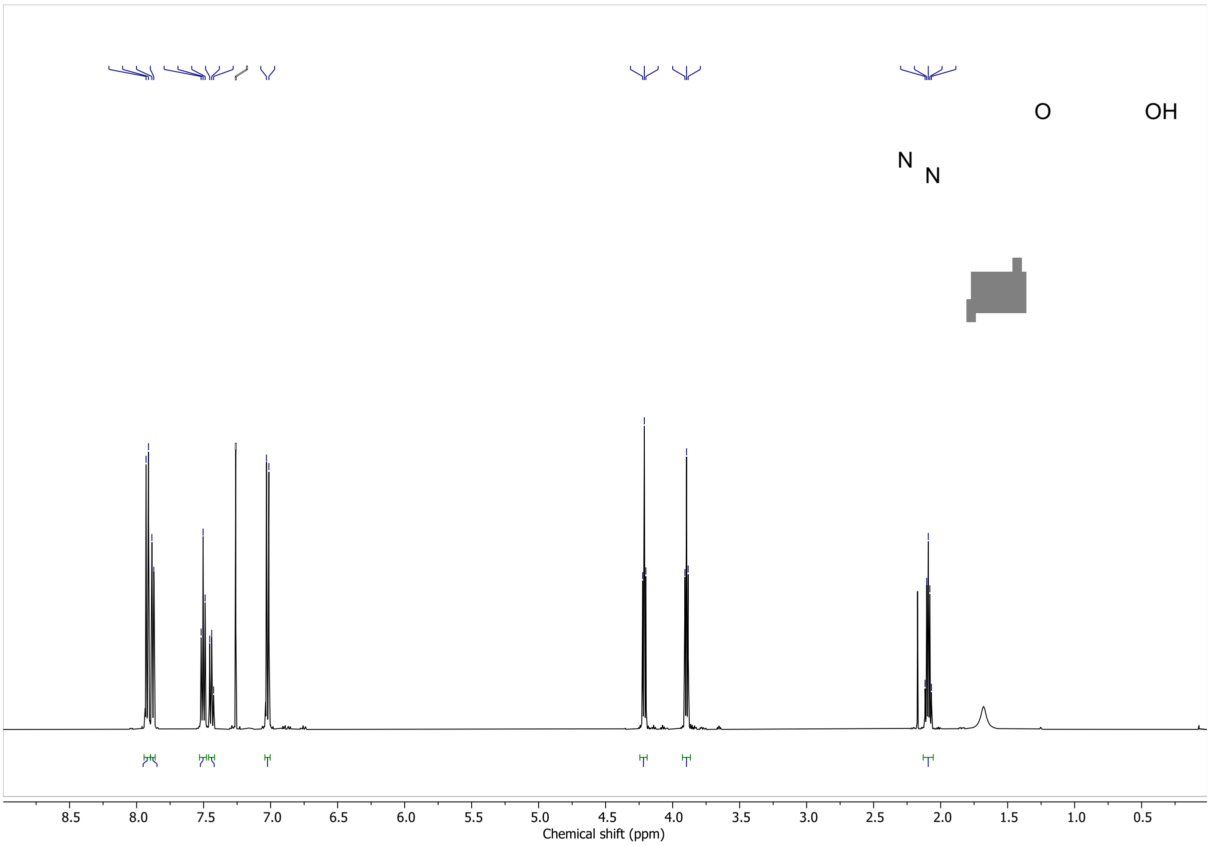


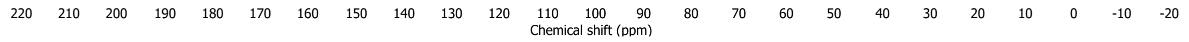


Figure S23: $^1$H NMR and $^{13}$C NMR spectra of 4-(propyl-1-oxy-3-ol)-azobenzene (**Azo**) in $CDCl_3$.

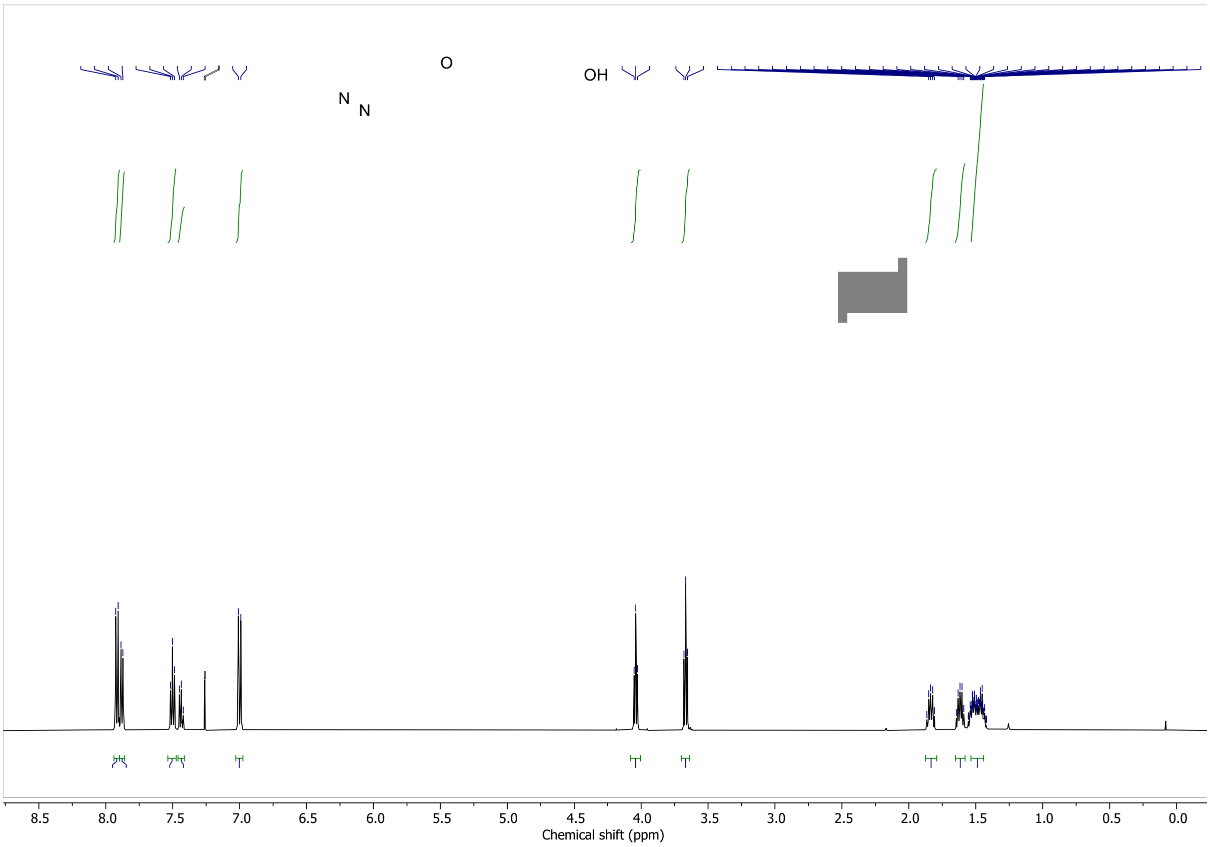


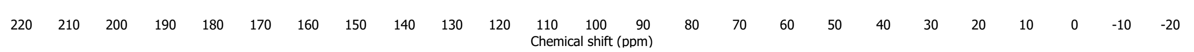


Figure S24: $^{1}H$ and $^{13}C$ NMR spectra of 4-(hexyl-1-oxy-3-ol)-azobenzene (**C₆-Azo**) in $CDCl_3$.

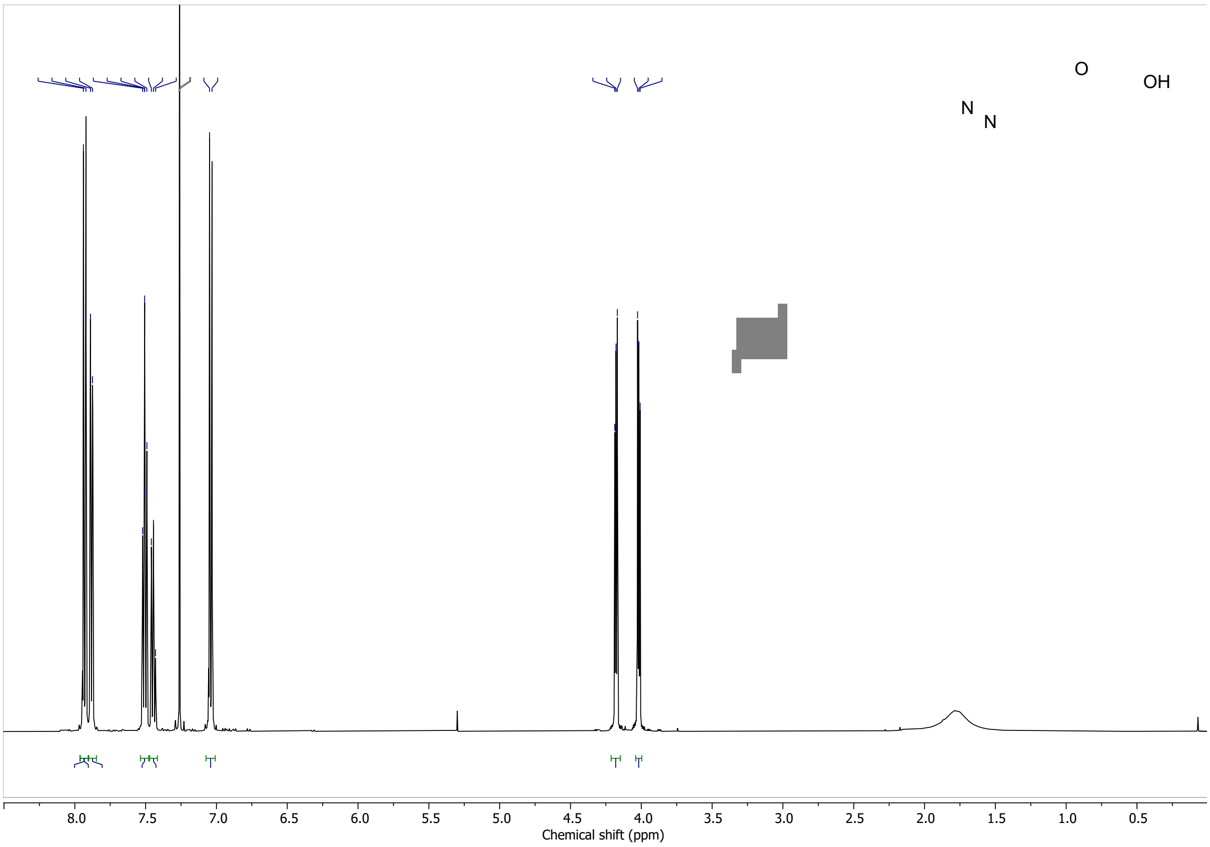


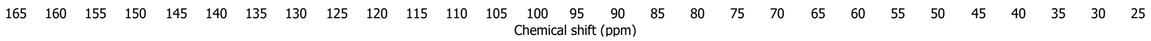


Figure S25: $^{1}H$ and $^{13}C$ NMR spectra of 4-(ethyl-1-oxy-3-ol)-azobenzene (**C₂-Azo**) in $CDCl_3$.

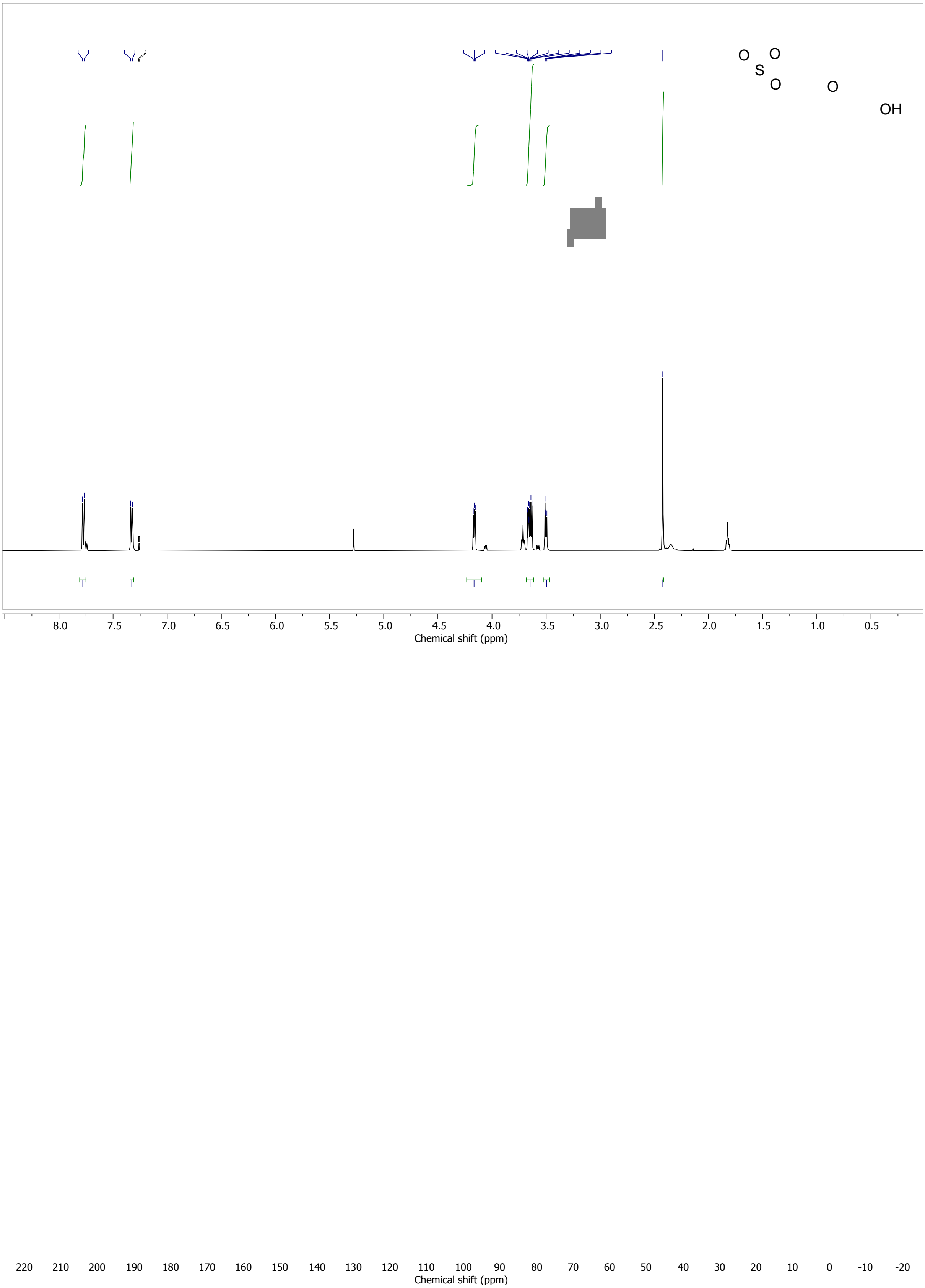


Figure S26: $^{1}H$ and $^{13}C$ NMR spectra of di(ethylene glycol) mono(*p*-toluenesulfonate) in $CDCl_3$.

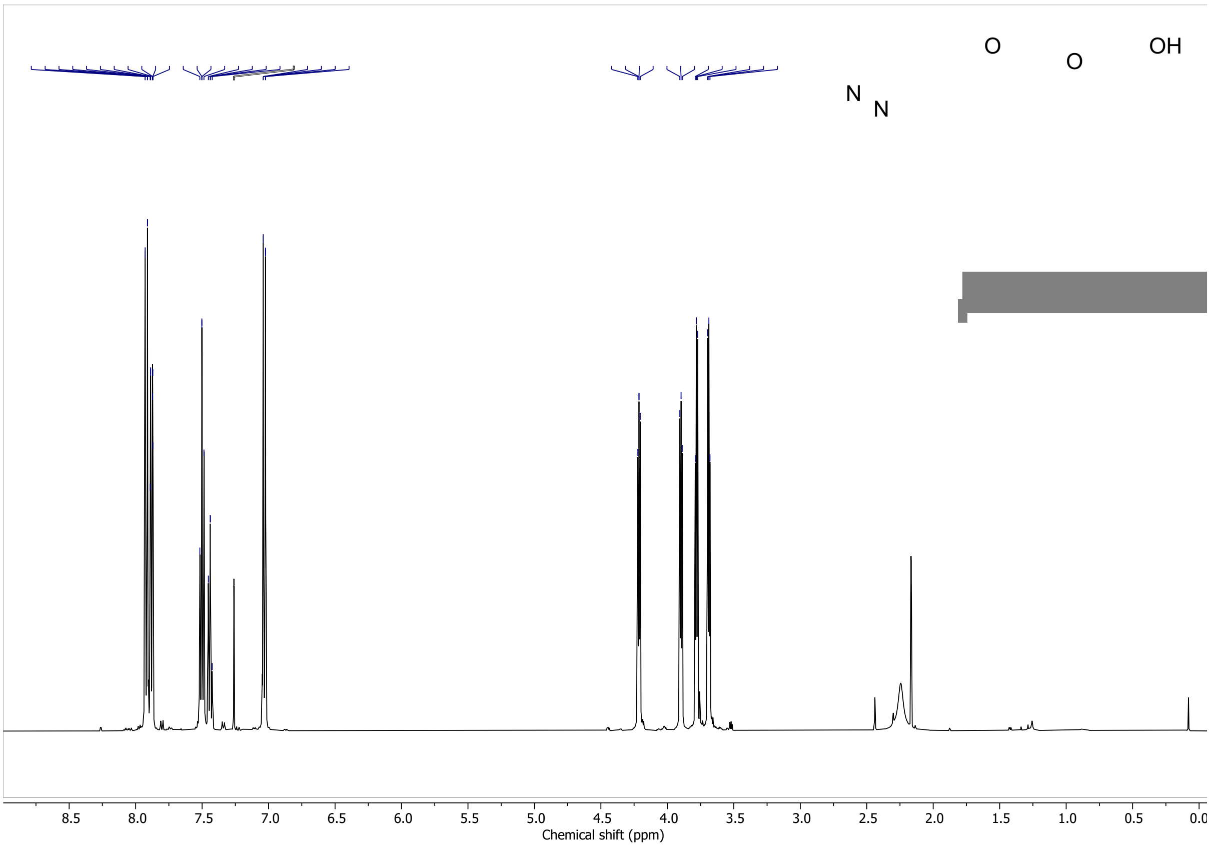


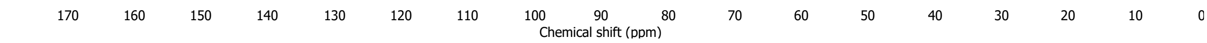


Figure S27: $^1H$ and $^{13}C$ NMR spectra of 4-di(ethylene glycol) azobenzene (**DEG-Azo**) in $CDCl_3$.

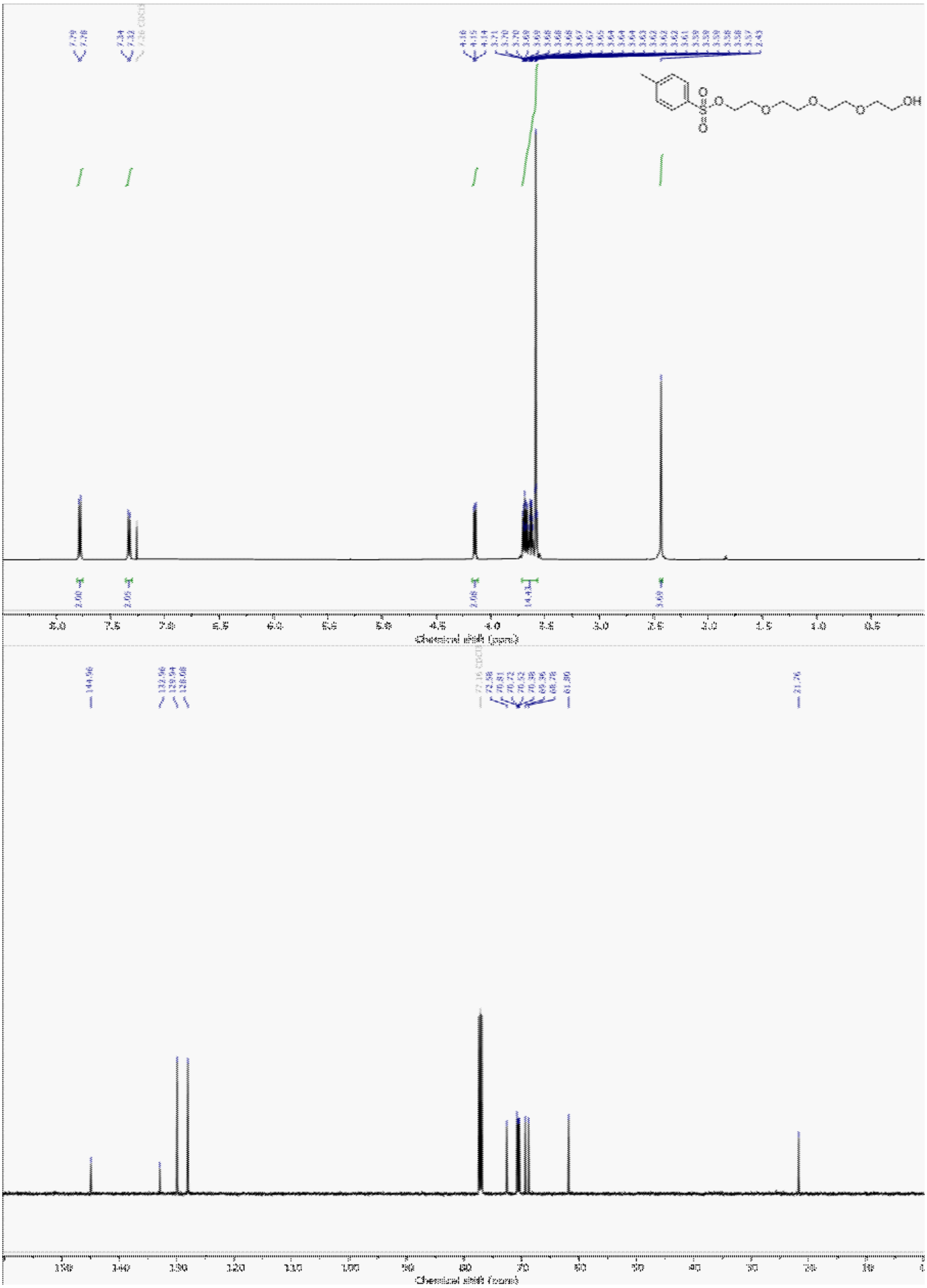

Figure S28: $^{1}H$ and $^{13}C$ NMR spectra of tetra(ethylene glycol) mono(*p*-toluenesulfonate) in $CDCl_3$.

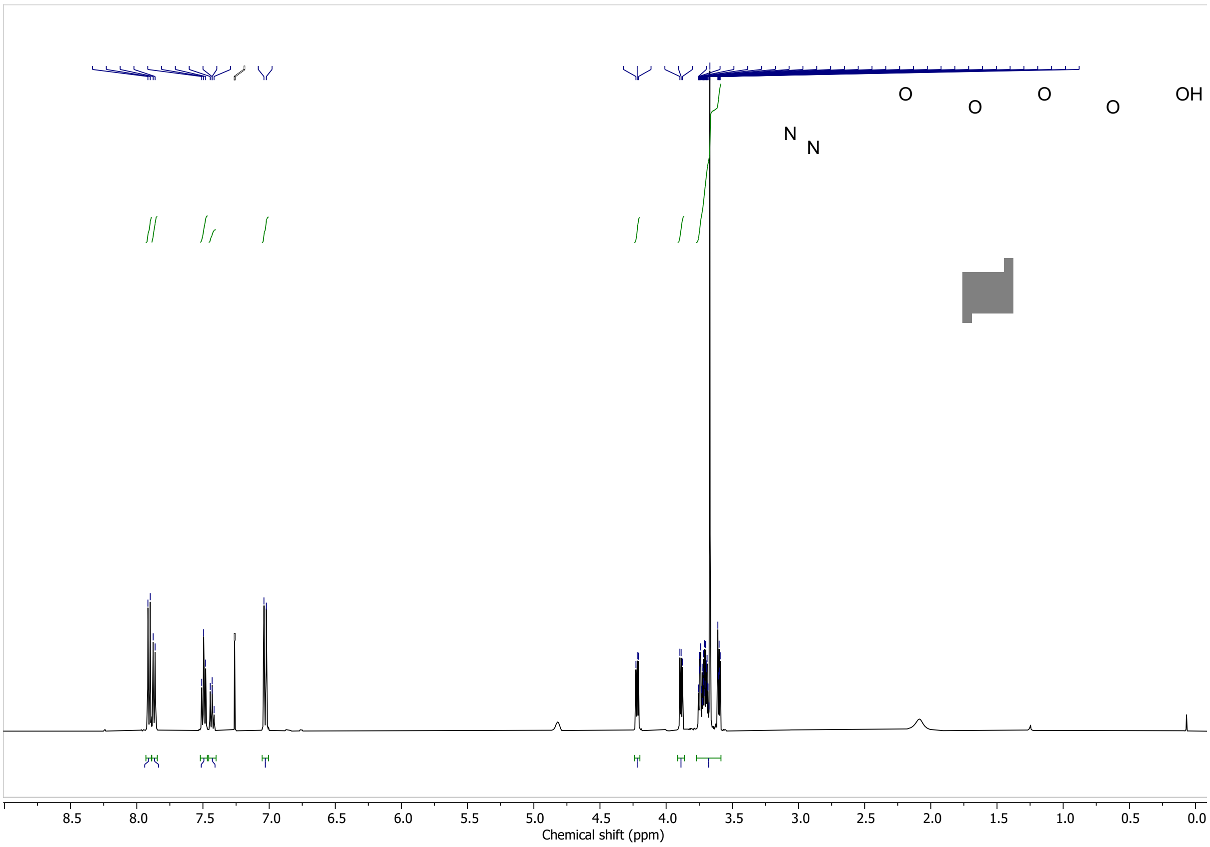


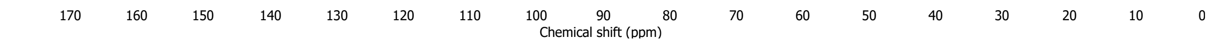


Figure S29: $^{1}H$ and $^{13}C$ NMR spectra of 4-tetra(ethylene glycol) azobenzene (**TEG-Azo**) in $CDCl_3$.

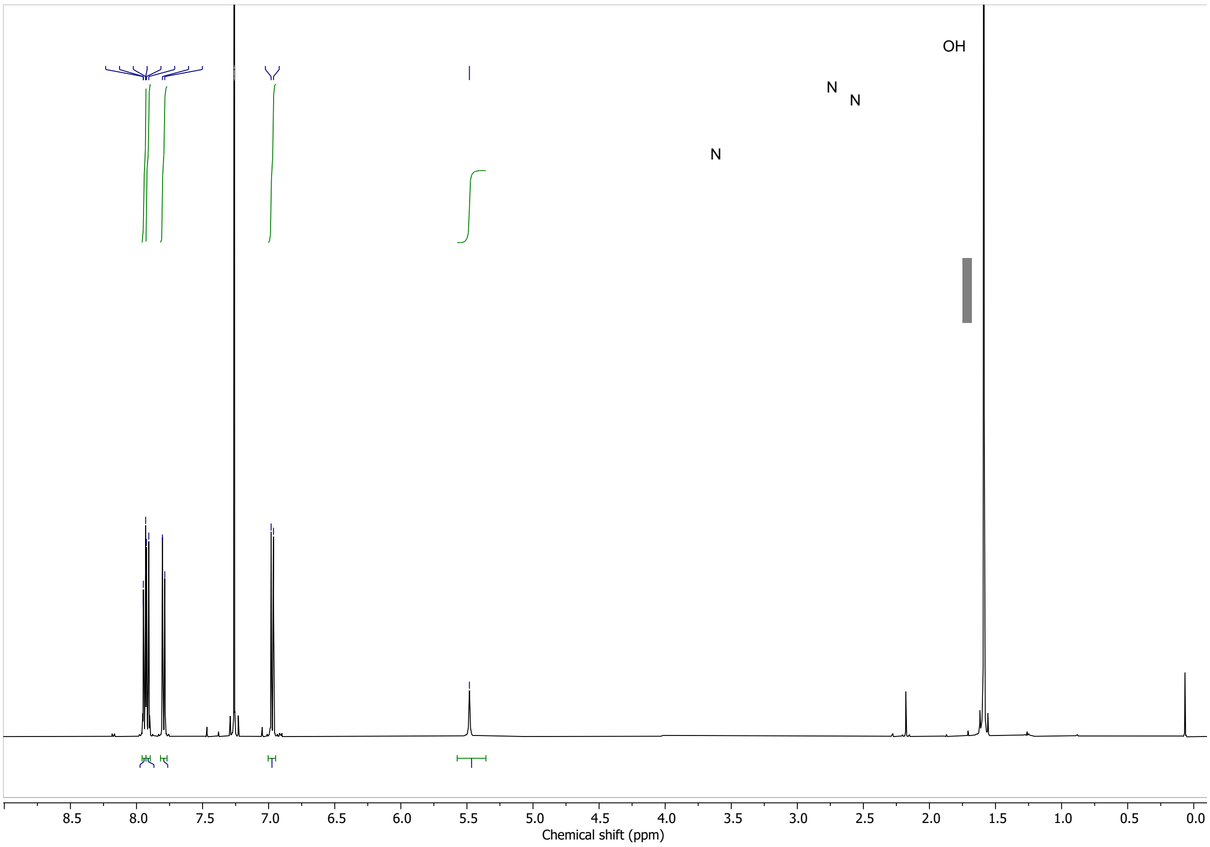


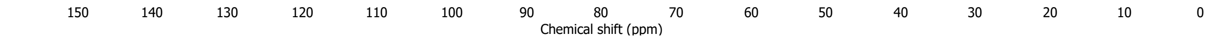


Figure S30: $^1$H and $^{13}$C NMR spectra of 4-cyano-4'-hydroxyazobenzene in $CDCl_3$.

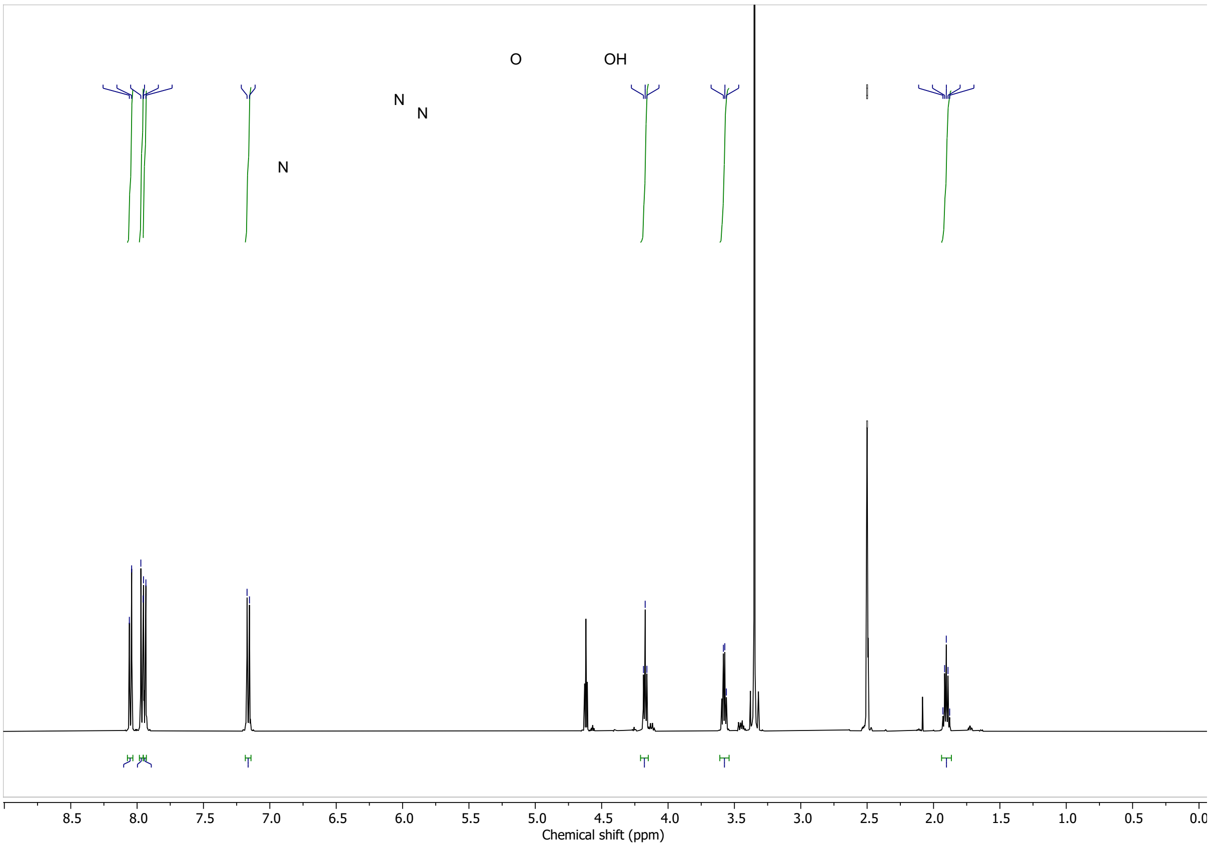


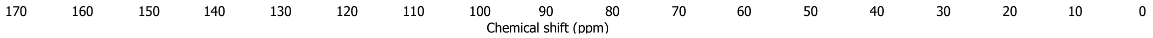


Figure S31: $^{1}H$ and $^{13}C$ NMR spectra of 4-cyano-4'-(propyl-1-oxy-3-ol) azobenzene in DMSO-$d_6$.

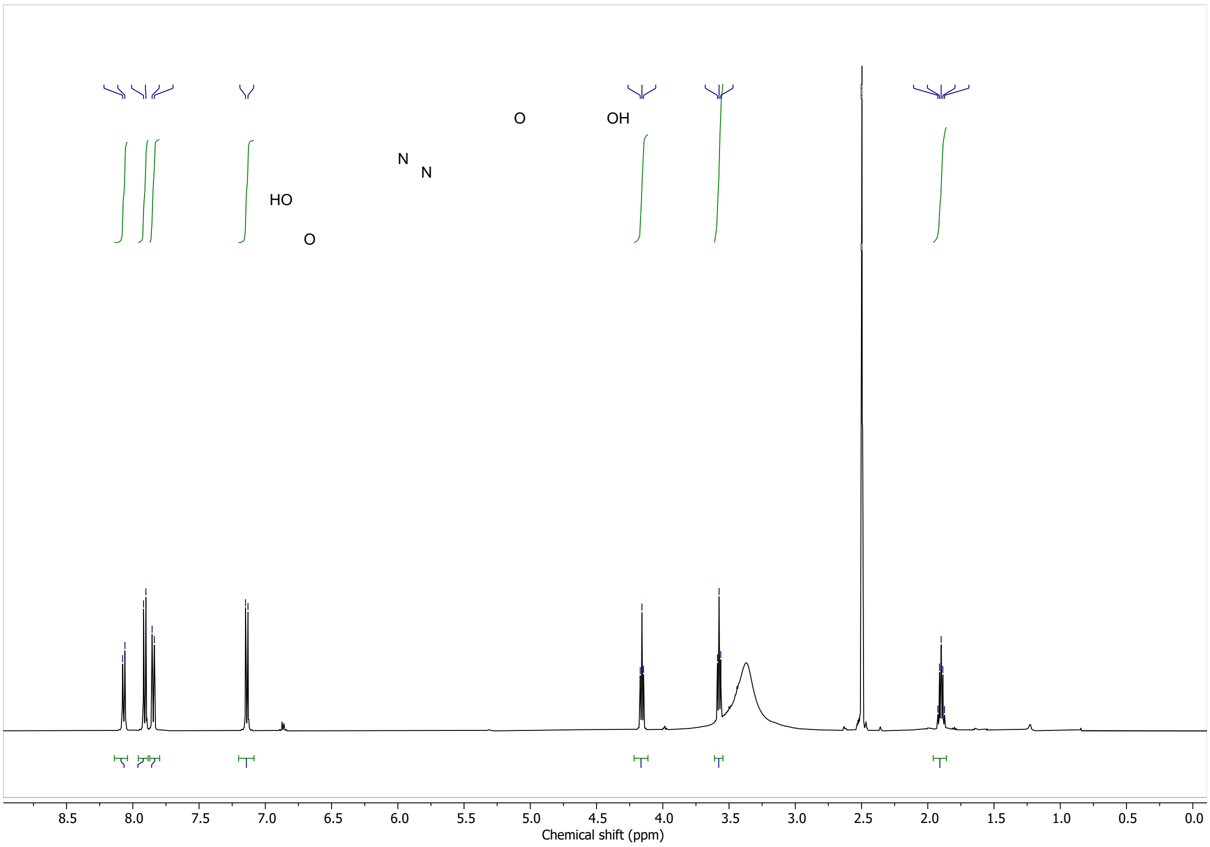


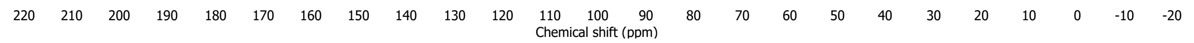


Figure S32: $^{1}$H and $^{13}$C NMR spectra of 4-carboxy-4'-(propyl-1-oxy-3-ol) azobenzene (**Azo-COOH**) in DMSO-$d_6$.

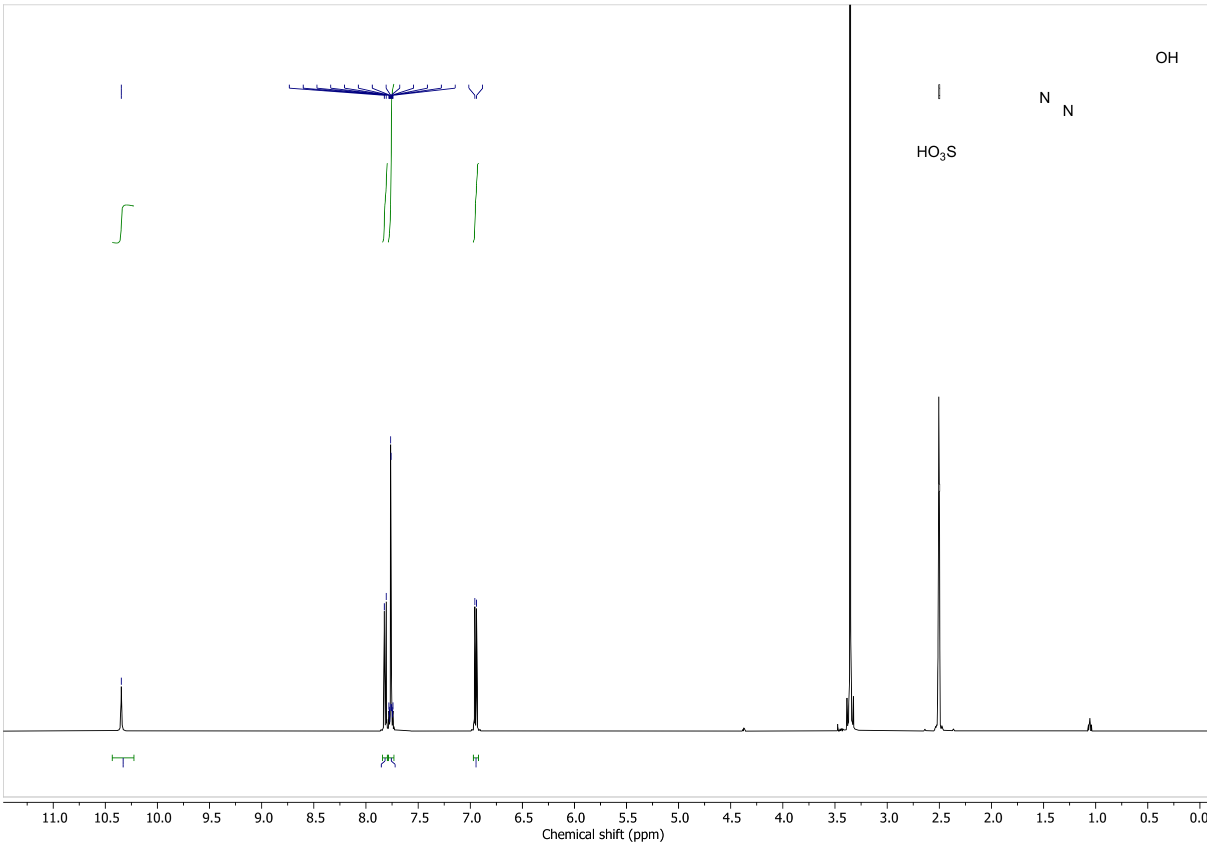


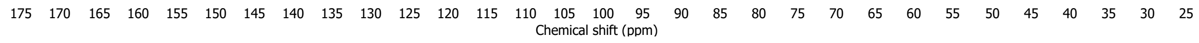


Figure S33: $^{1}H$ and $^{13}C$ NMR spectra of 4-(4'-hydroxy)azobenzene sulfonic acid in DMSO-$d_6$.

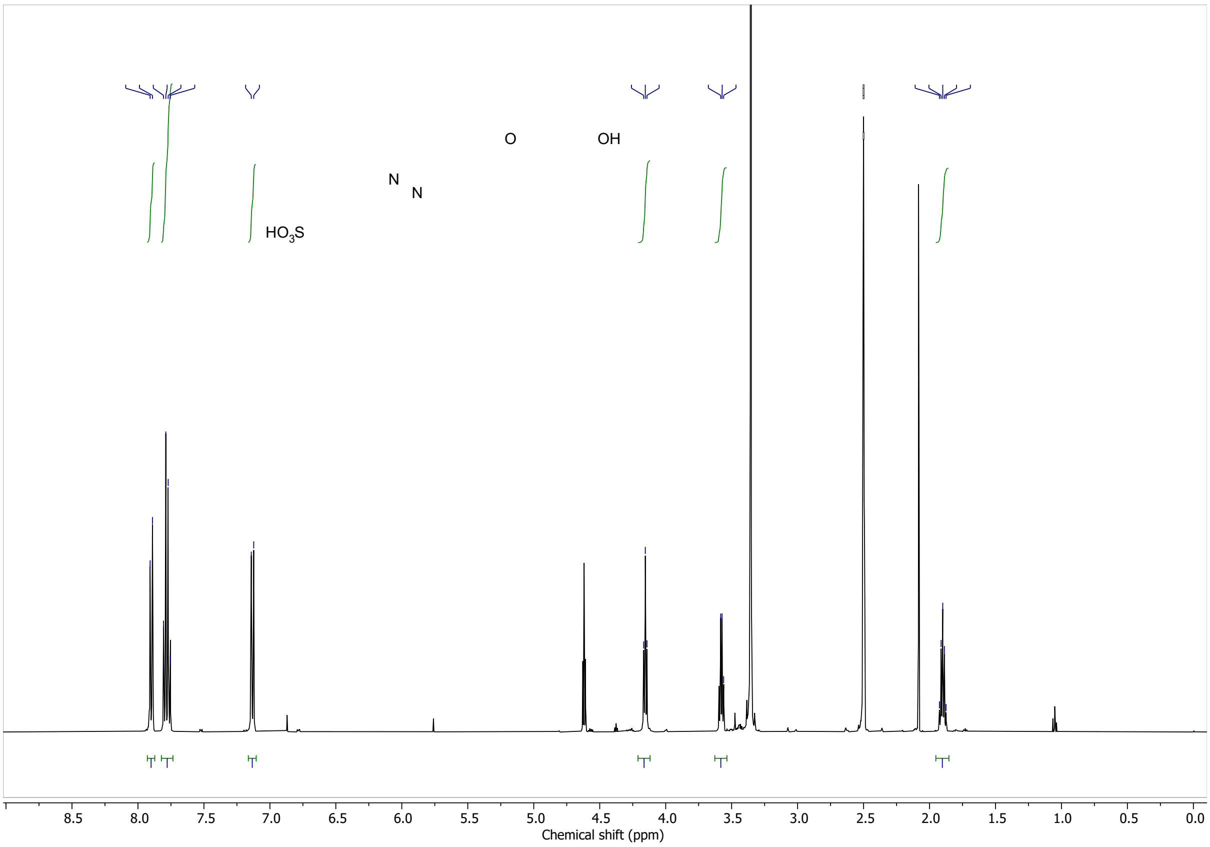


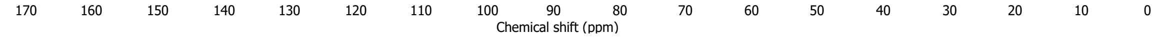


Figure S34: $^{1}H$ and $^{13}C$ NMR spectra of 4-(4'-propyl-1-oxy-3-ol) azobenzene sulfonic acid (**Azo-SO₃H**) in DMSO-$d_6$.

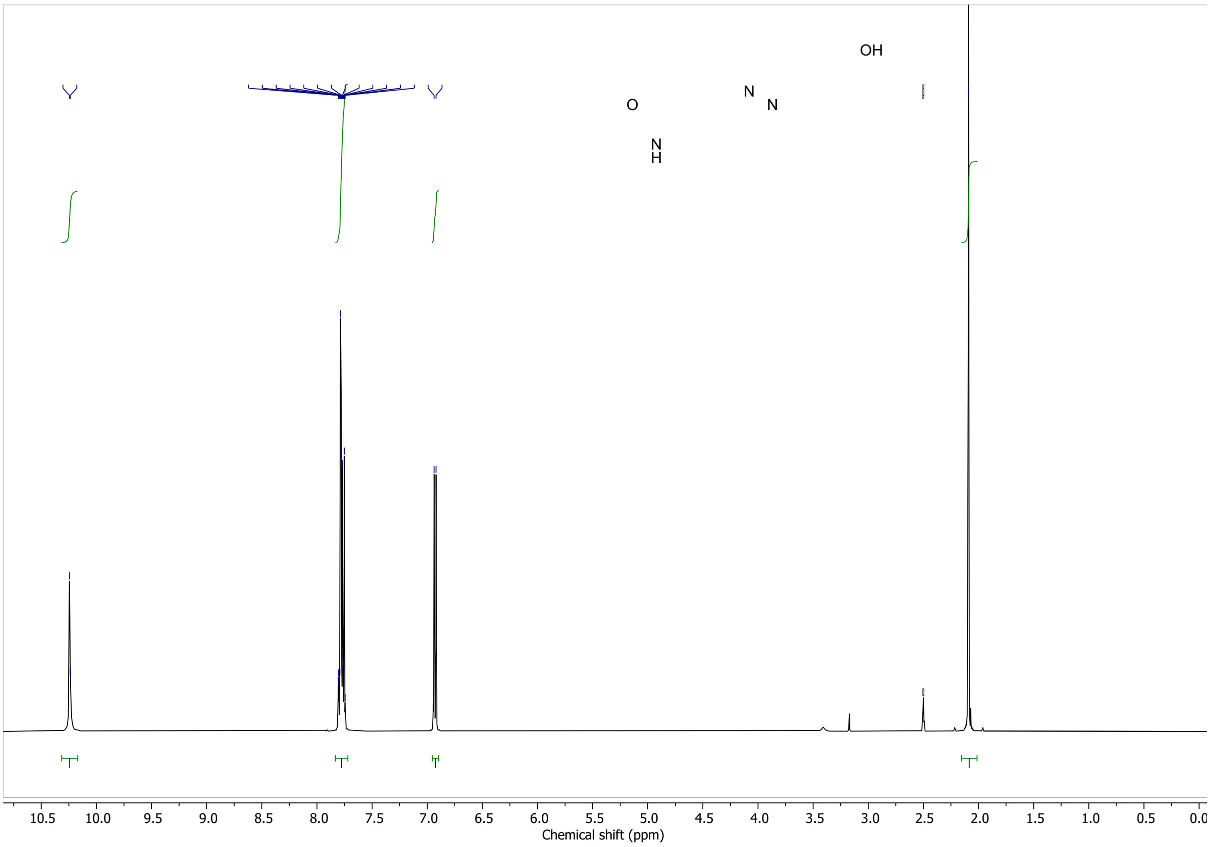


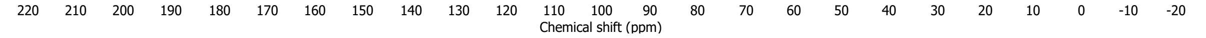


FigureS 35: $^{1}H$ and $^{13}C$ NMR spectra of 4-acetylamido-4'-hydroxyazobenzene in DMSO-$d_6$.

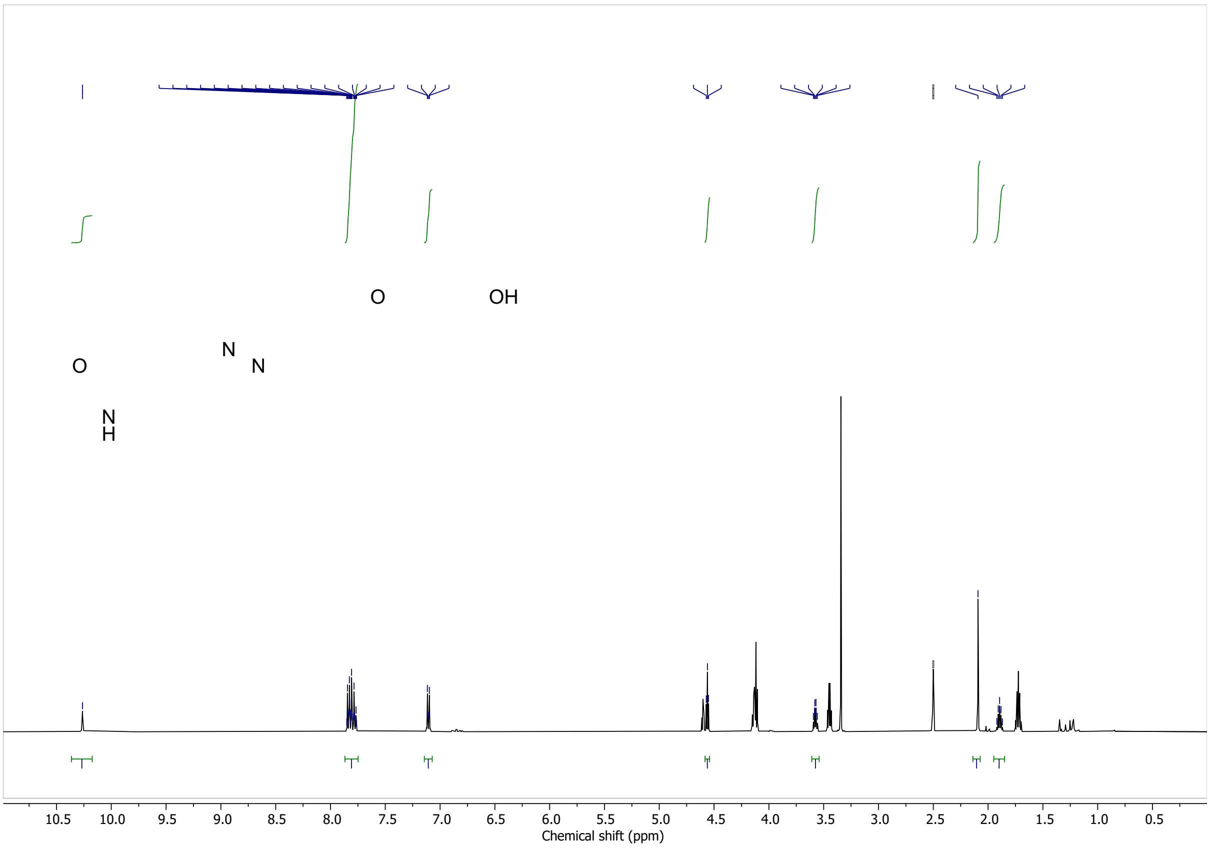


170 160 150 140 130 120 110 100 90 80 70 60 50 40 30 20 10
Chemical shift (ppm)

Figure S36: $^{1}H$ and $^{13}C$ NMR spectra of 4-(4'- propyl-1-oxy-3-ol) azobenzene acetylamide in DMSO-$d_6$.

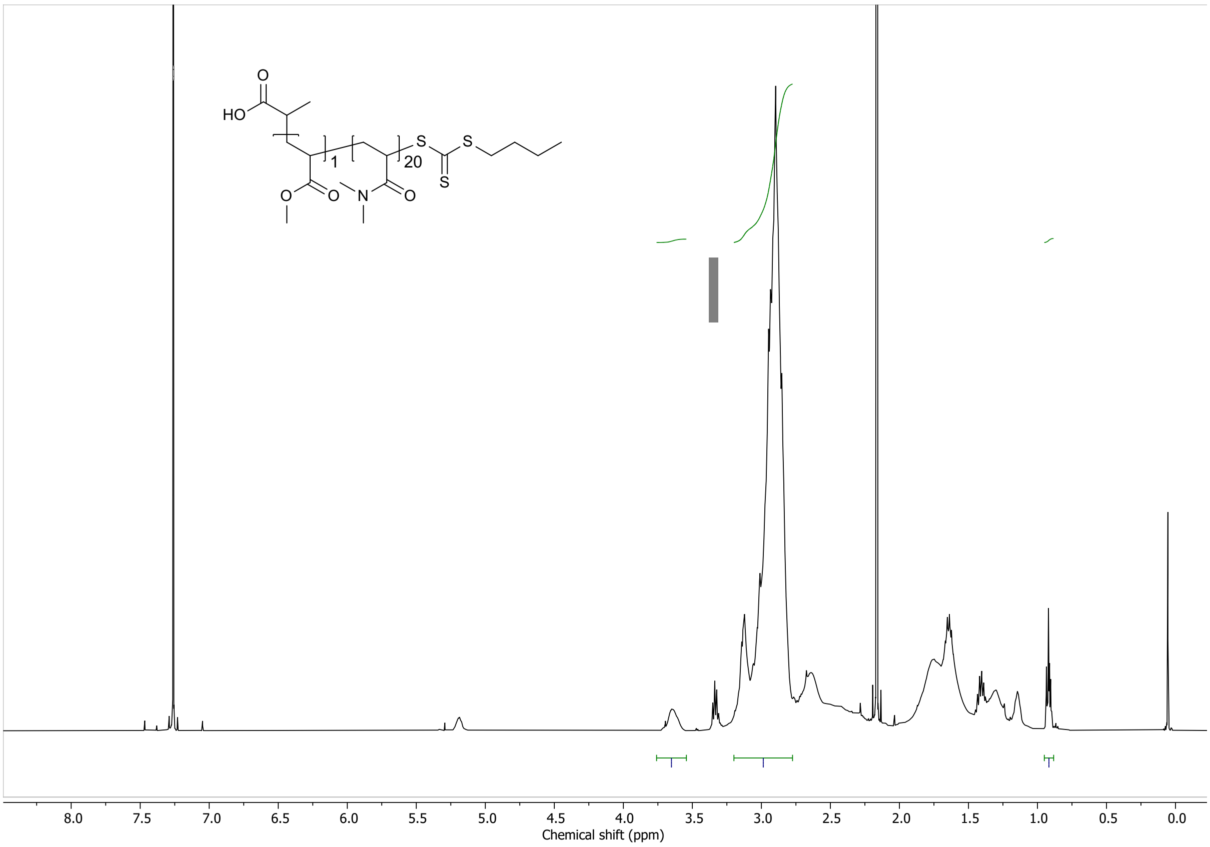


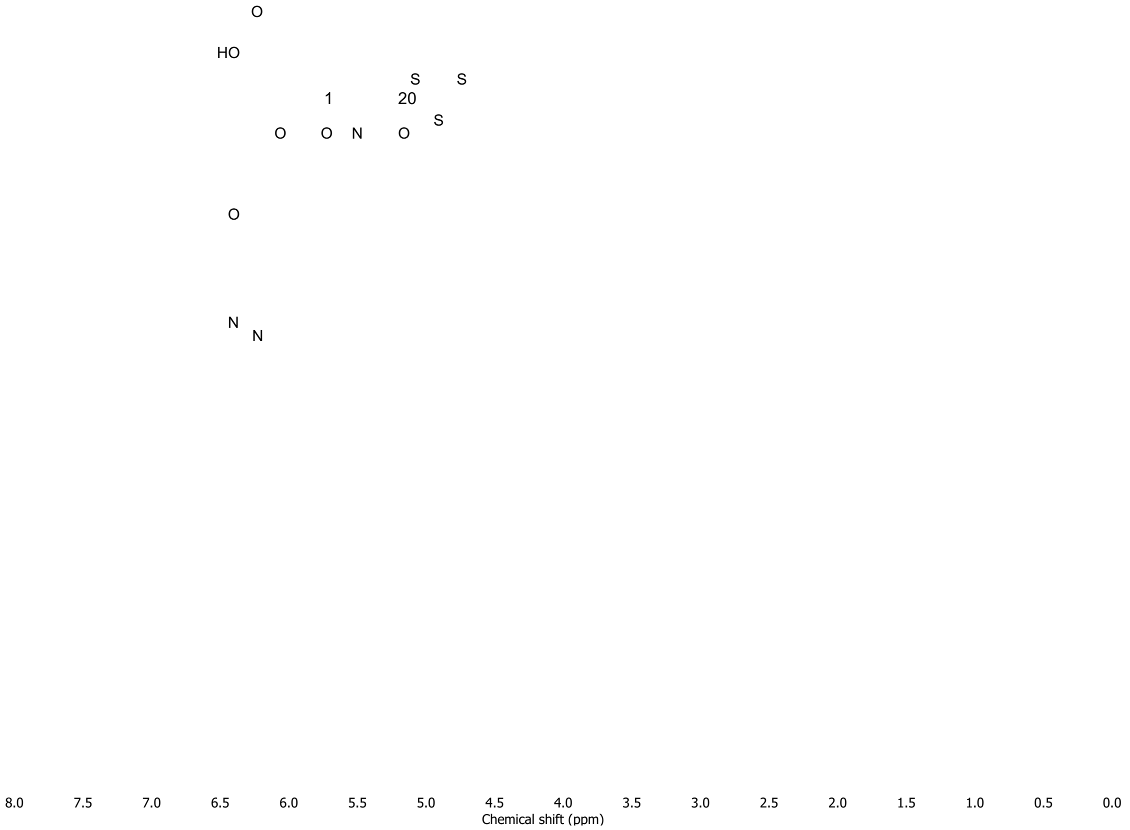


Figure S37: [1]H NMR spectra of p($DMA_{19}$-s-$MA_1$) and p($DMA_{19}$-s-$Azo_1$) in $CDCl_3$.

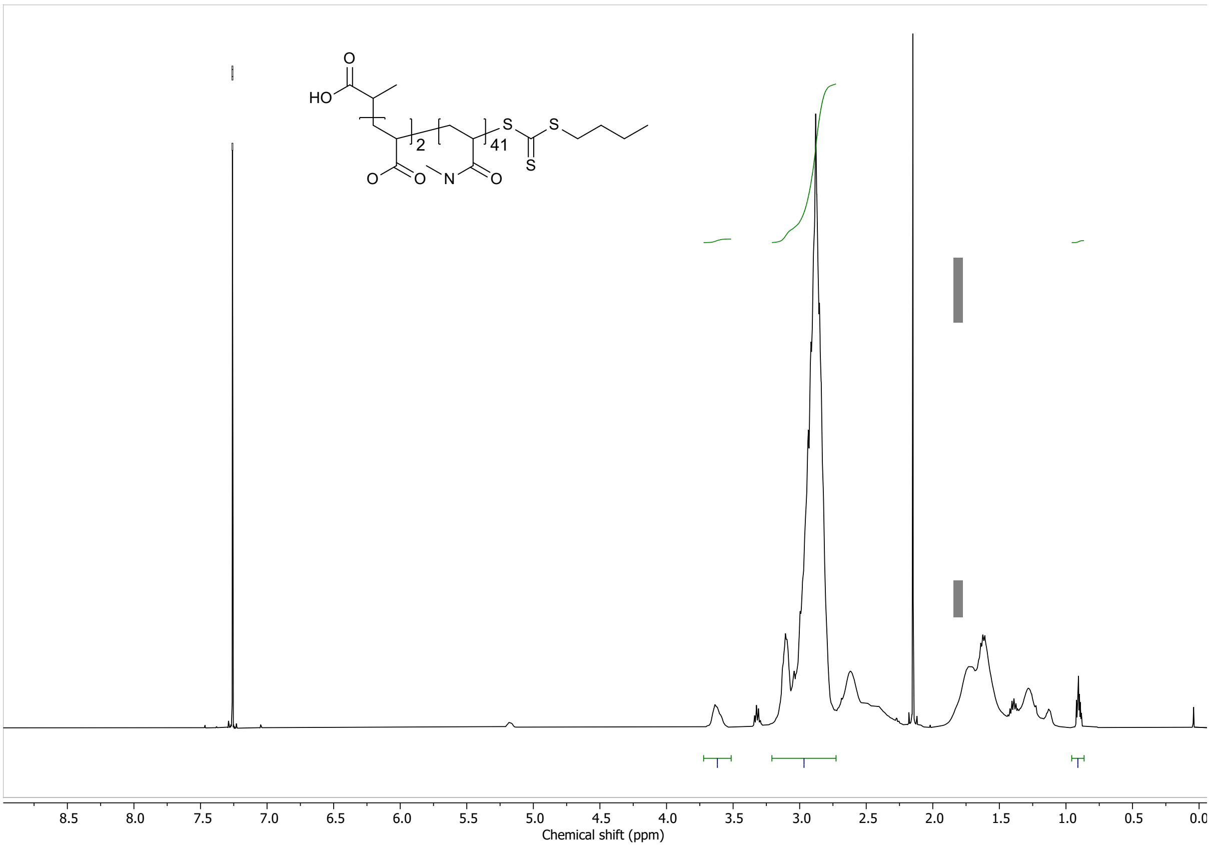


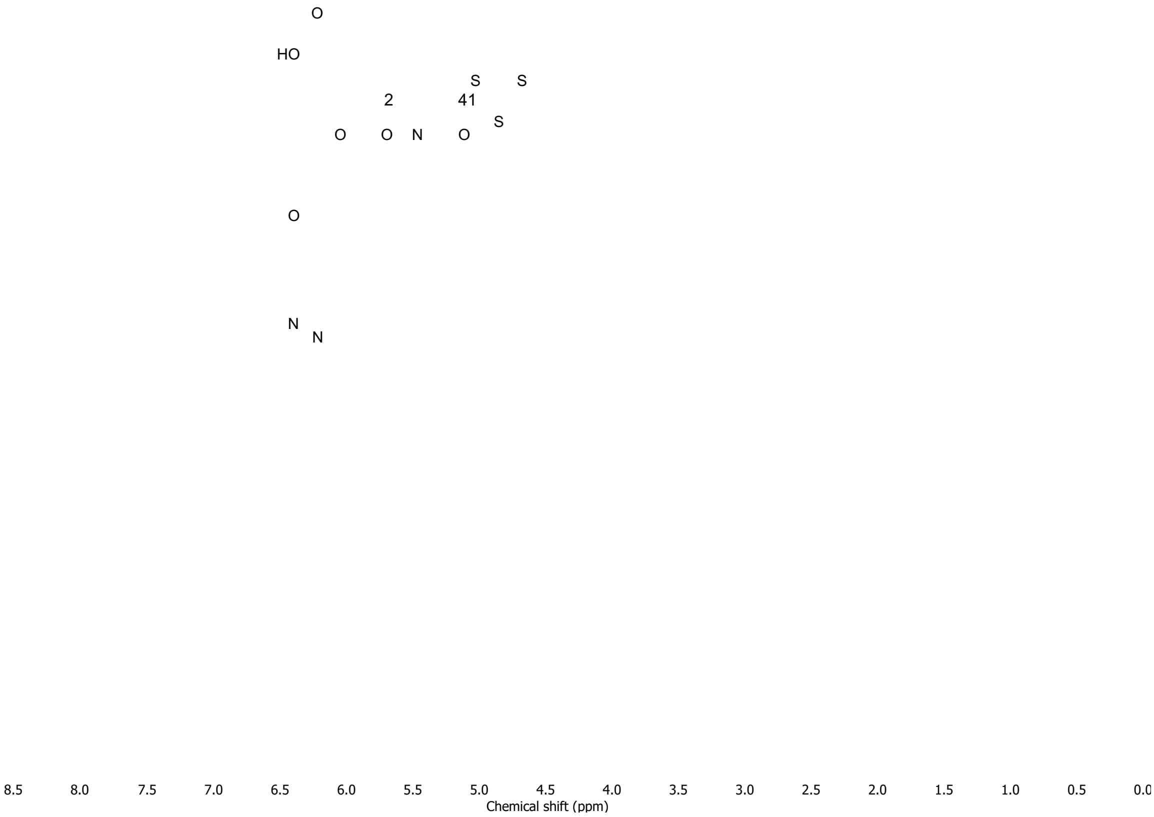


Figure S38: $^{1}$H NMR spectra of p(DMA$_{28}$-s-MA$_{2}$) and p(DMA$_{38}$-s-Azo$_{2}$) in CDCl$_{3}$.

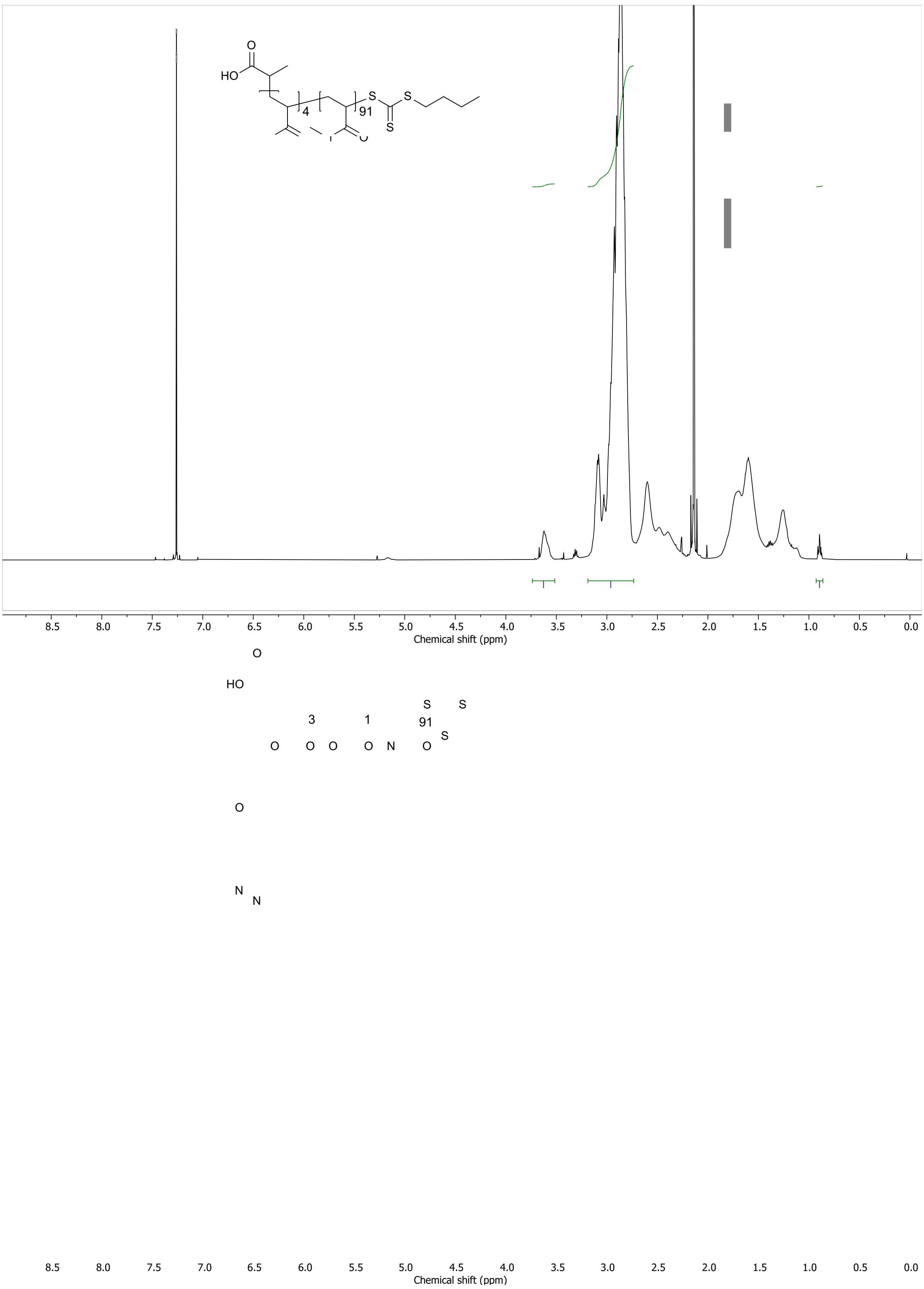


Figure S39: $^{1}$H NMR spectra of p($DMA_{95}$-s-$MA_5$) and p($DMA_{95}$-s-$Azo_5$) in $CDCl_3$.

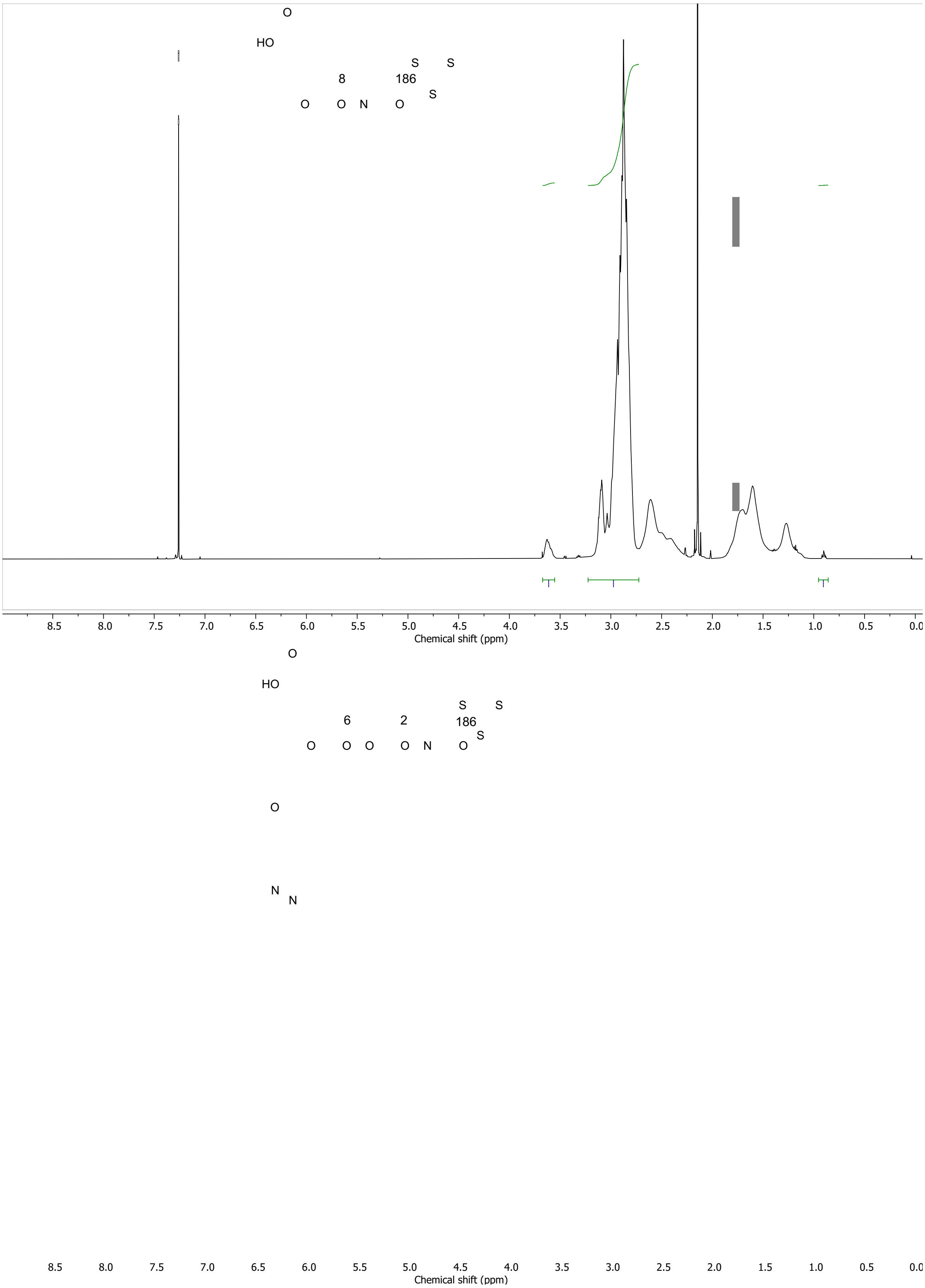


Figure S40: $^1H$ NMR spectra of p($DMA_{190}$-s-$MA_{10}$) and p($DMA_{190}$-s-$Azo_{10}$) in $CDCl_3$.

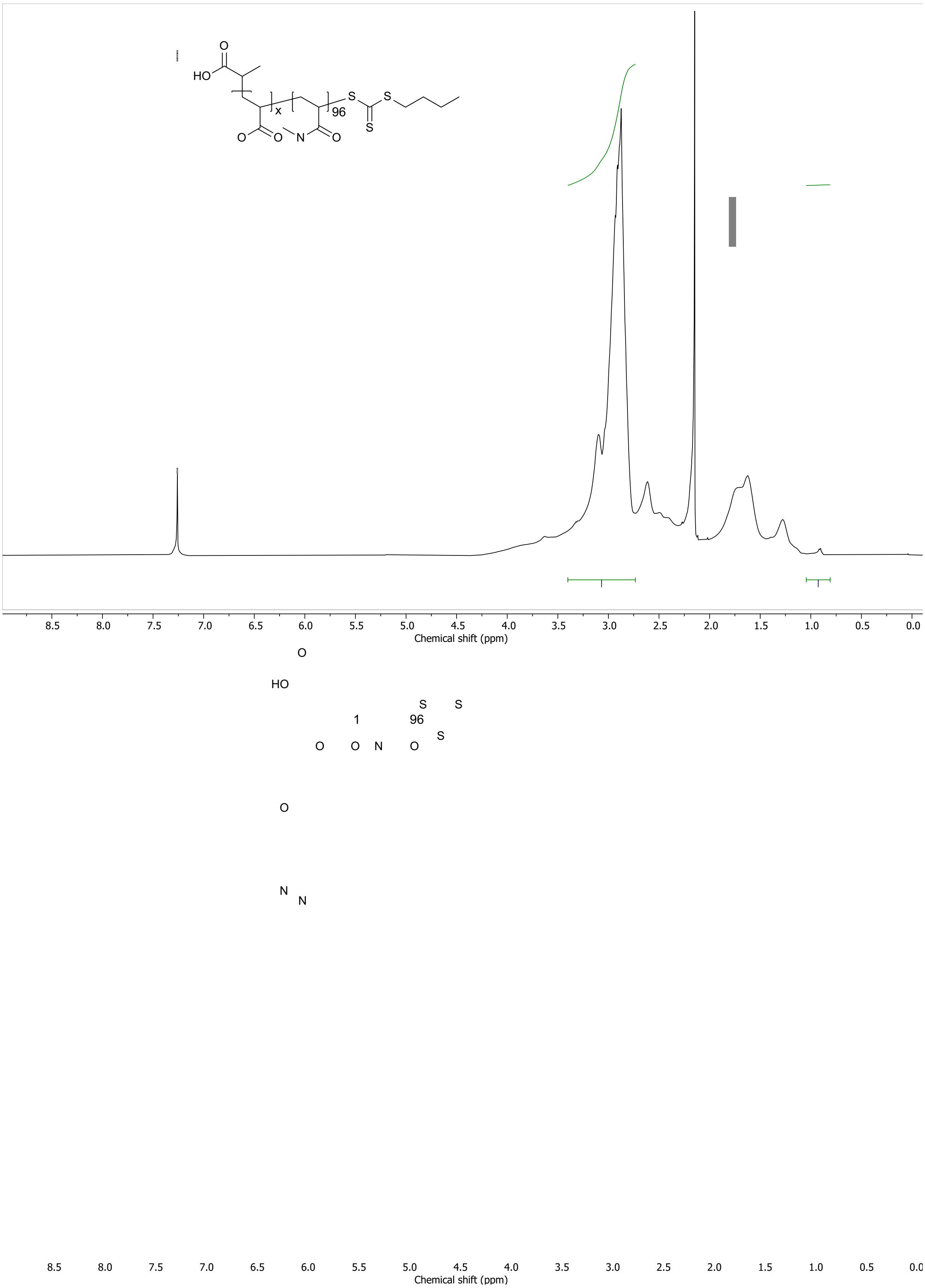


Figure S41: $^{1}$H NMR spectra of p(DMA$_{99}$-s-MA$_{1}$) and p(DMA$_{99}$-s-Azo$_{1}$) in $CDCl_3$.

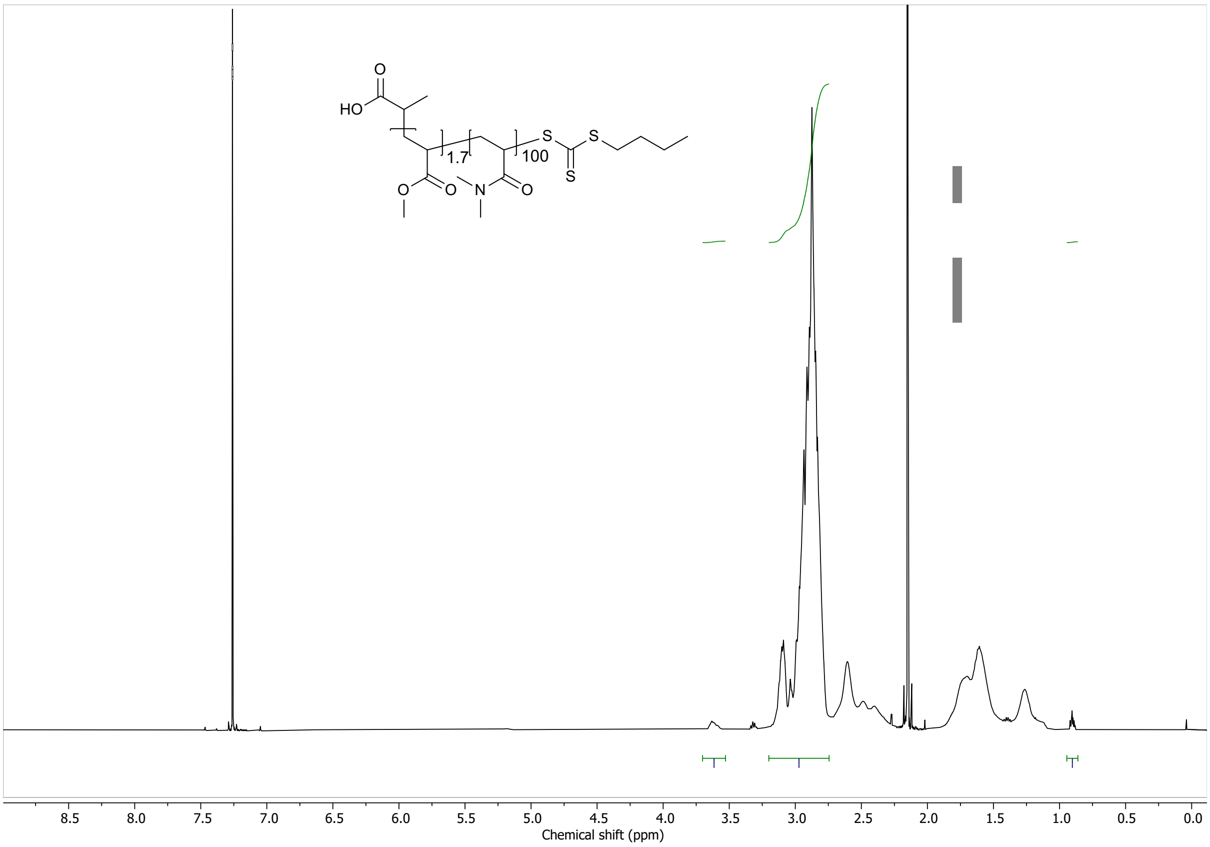


O

HO

S S

1.5 100

S

O O N O

O

N N

8.5 8.0 7.5 7.0 6.5 6.0 5.5 5.0 4.5 4.0 3.5 3.0 2.5 2.0 1.5 1.0 0.5 0.0

Chemical shift (ppm)

Figure S42: $^{1}H$ NMR spectra of p($DMA_{98}$-s-$MA_2$) and p($DMA_{98}$-s-$Azo_2$) in $CDCl_3$.

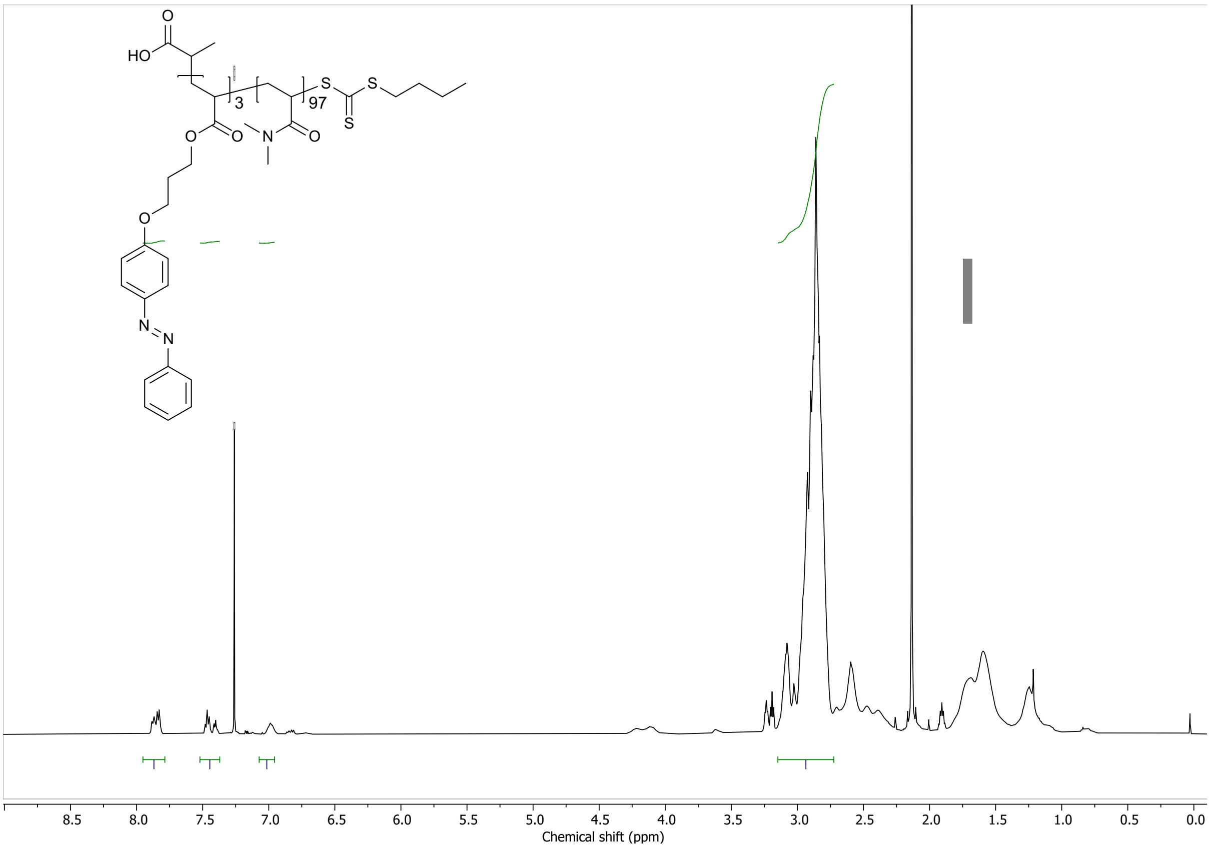


Figure S43: $^1$H NMR spectum of p($DMA_{97}$-s-$Azo_3$) in $CDCl_3$.

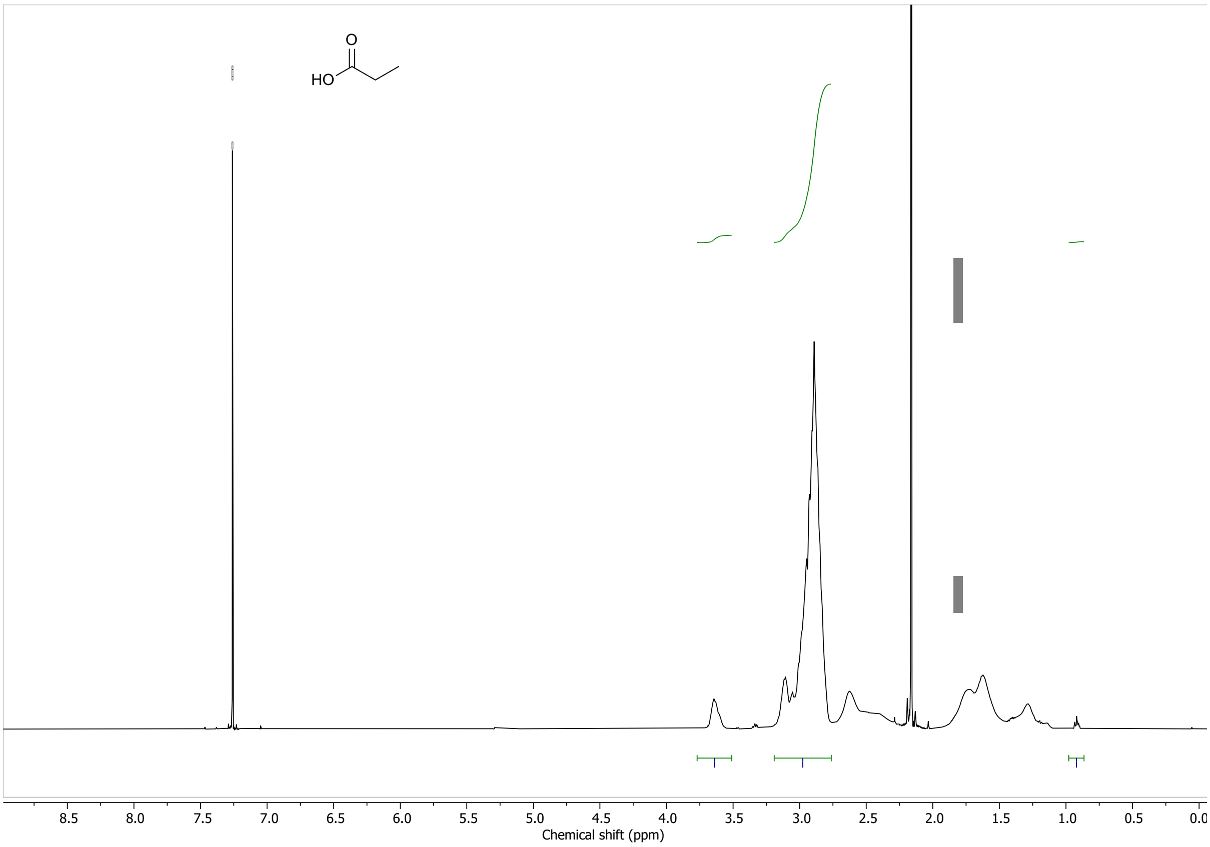


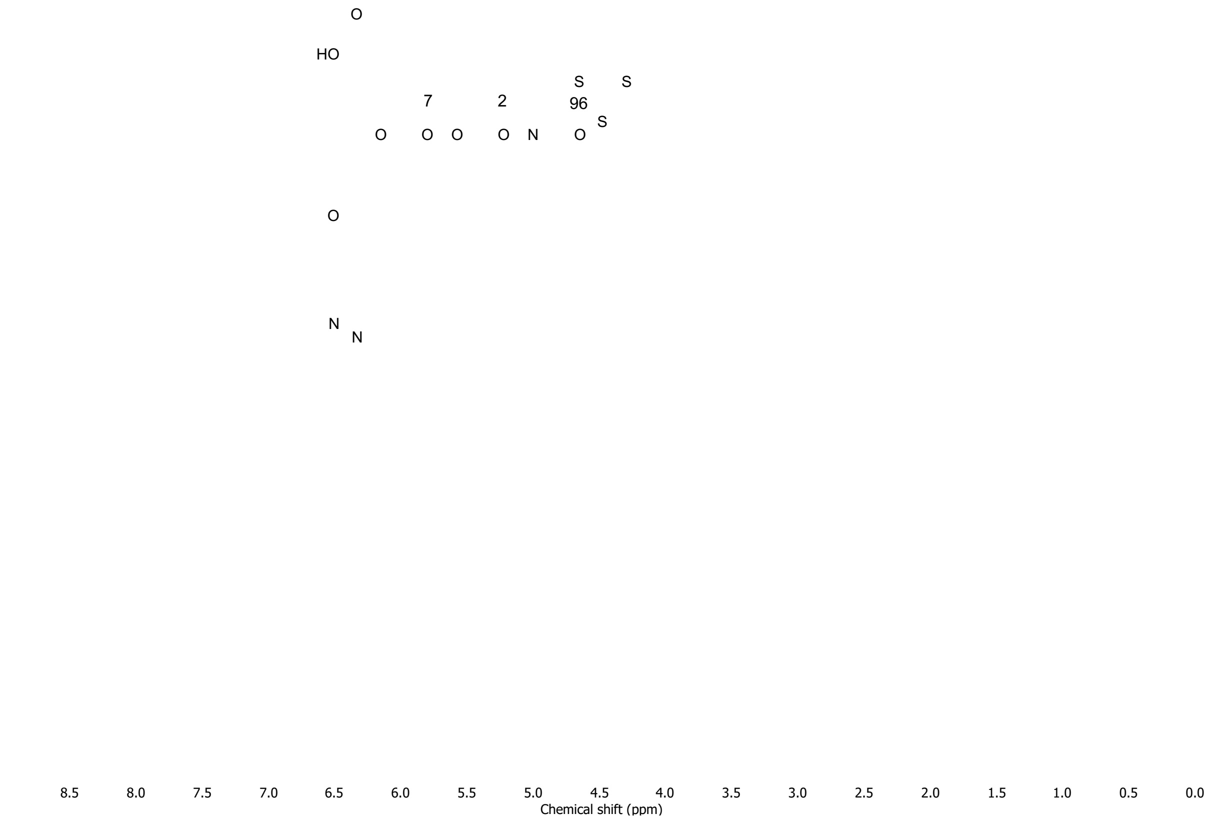


Figure S44: $^1$H NMR spectra of p(DMA$_{90}$-s-MA$_{10}$) and p(DMA$_{90}$-s-Azo$_{10}$) in $CDCl_3$.

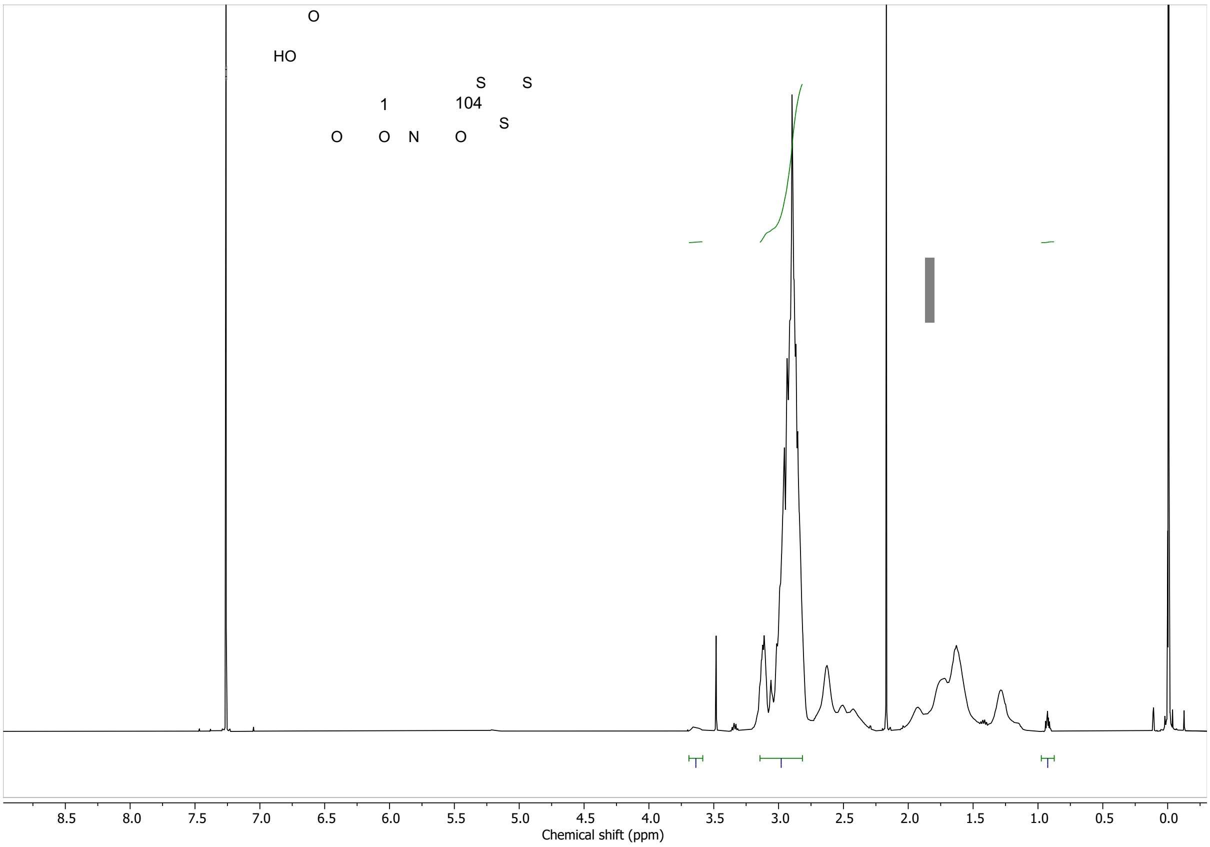


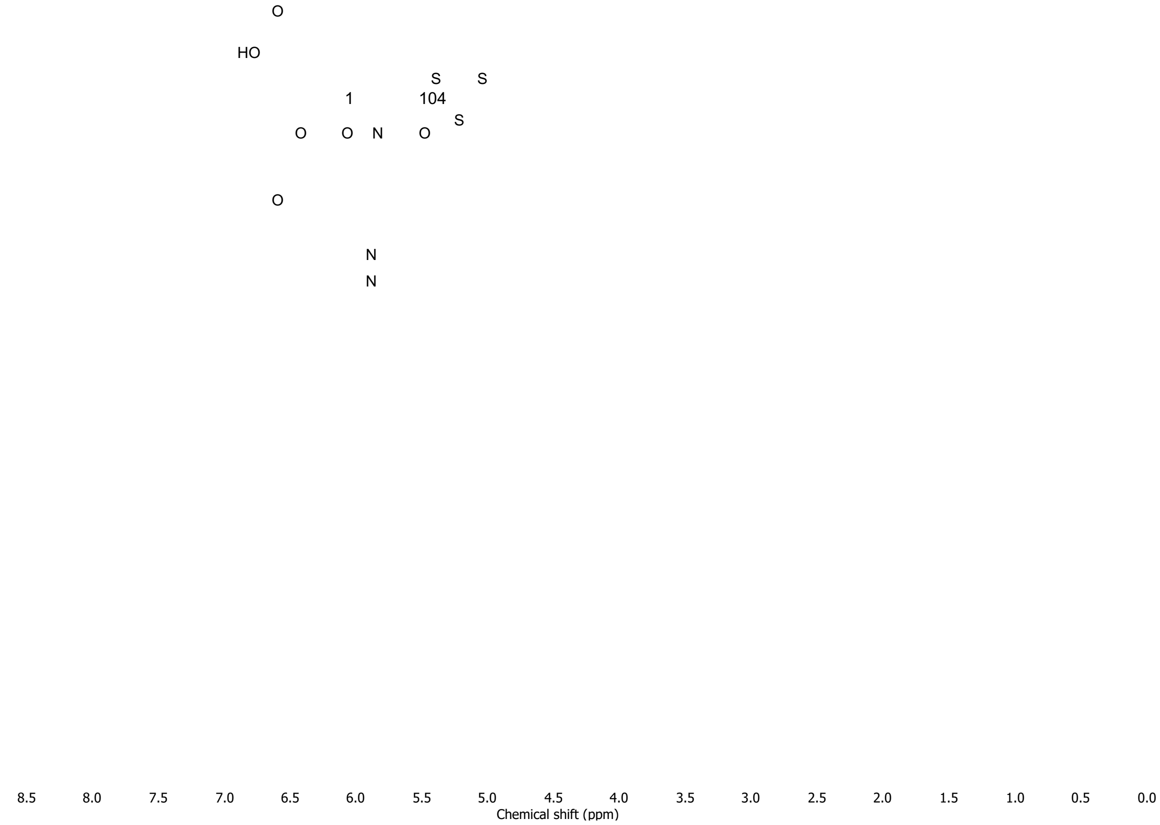


Figure S45: $^1$H NMR spectra of p(DMA$_{99}$-s-MA$_1$) and p(DMA$_{99}$-s-[C$_2$-Azo]$_1$) in CDCl$_3$.

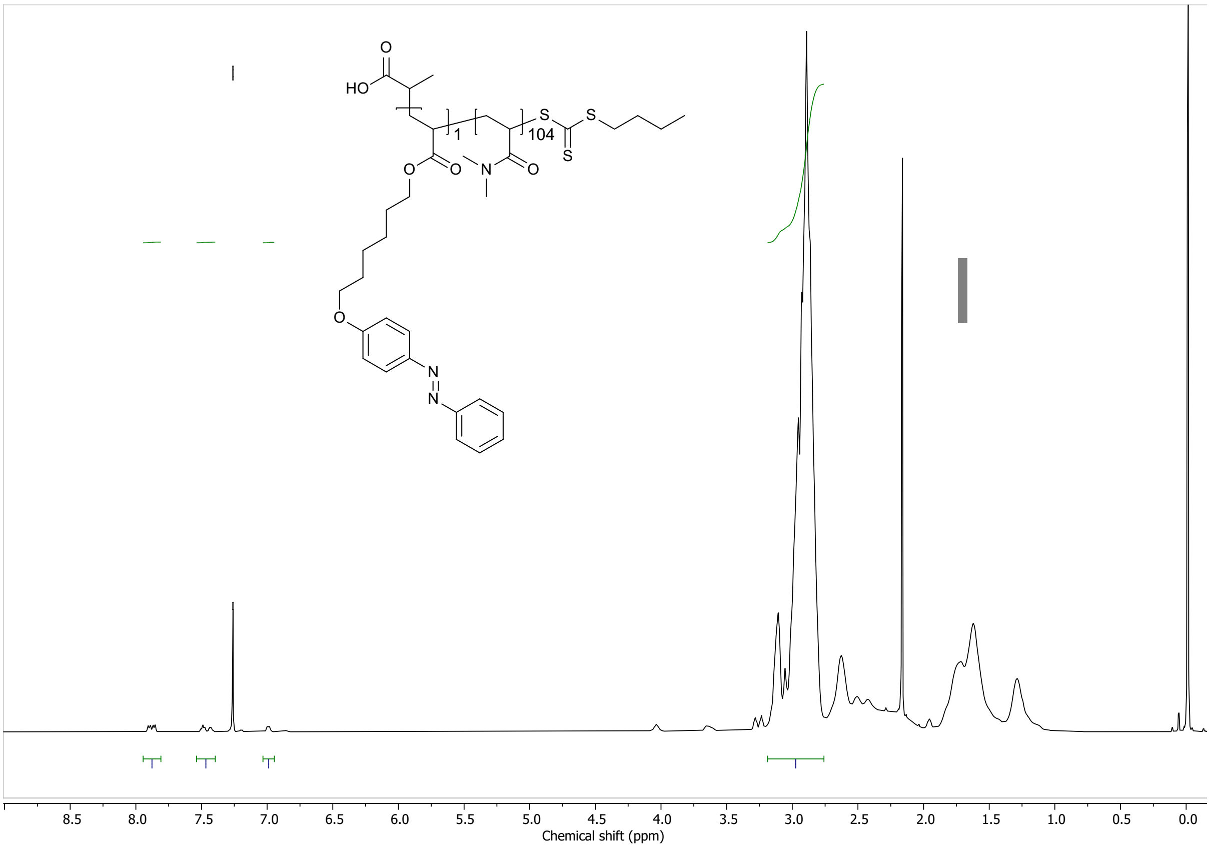


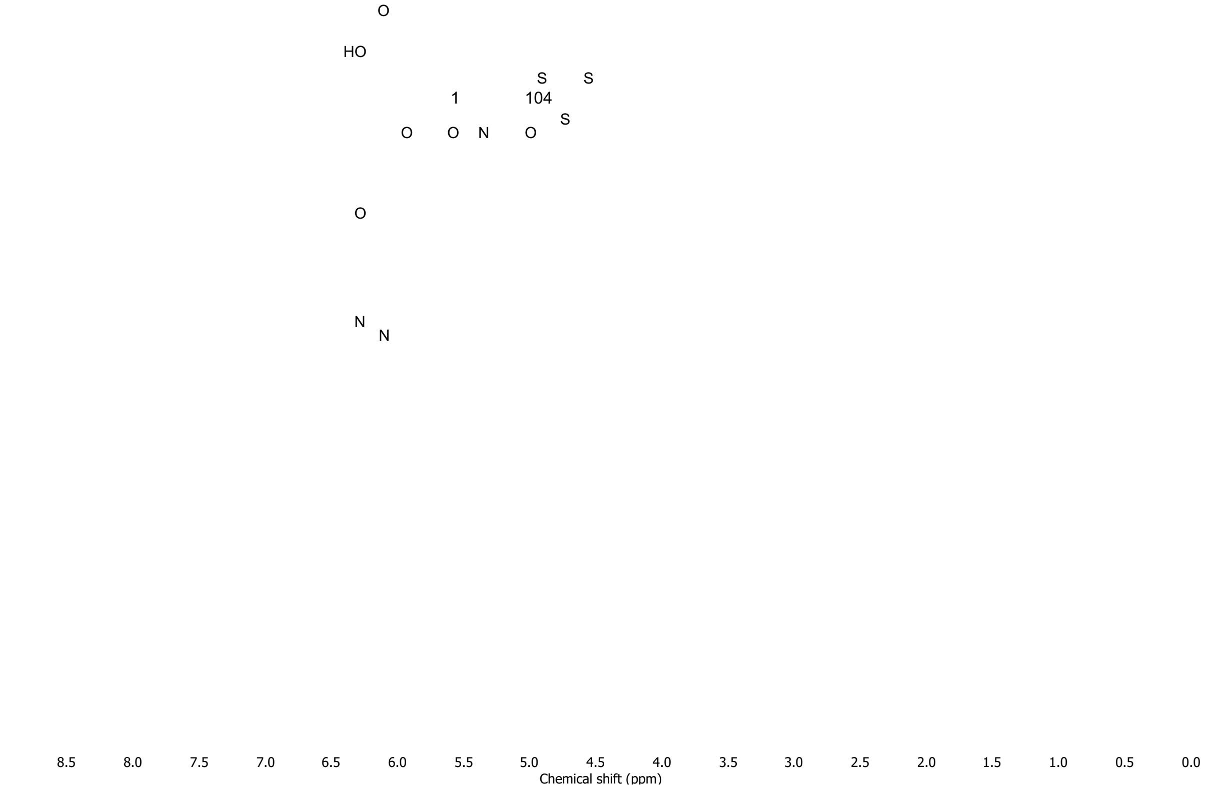


Figure S46: $^{1}$H NMR spectra of p($DMA_{99}$-s-[$C_6$-Azo]$_1$) and p($DMA_{99}$-s-$Azo_1$) in $CDCl_3$.

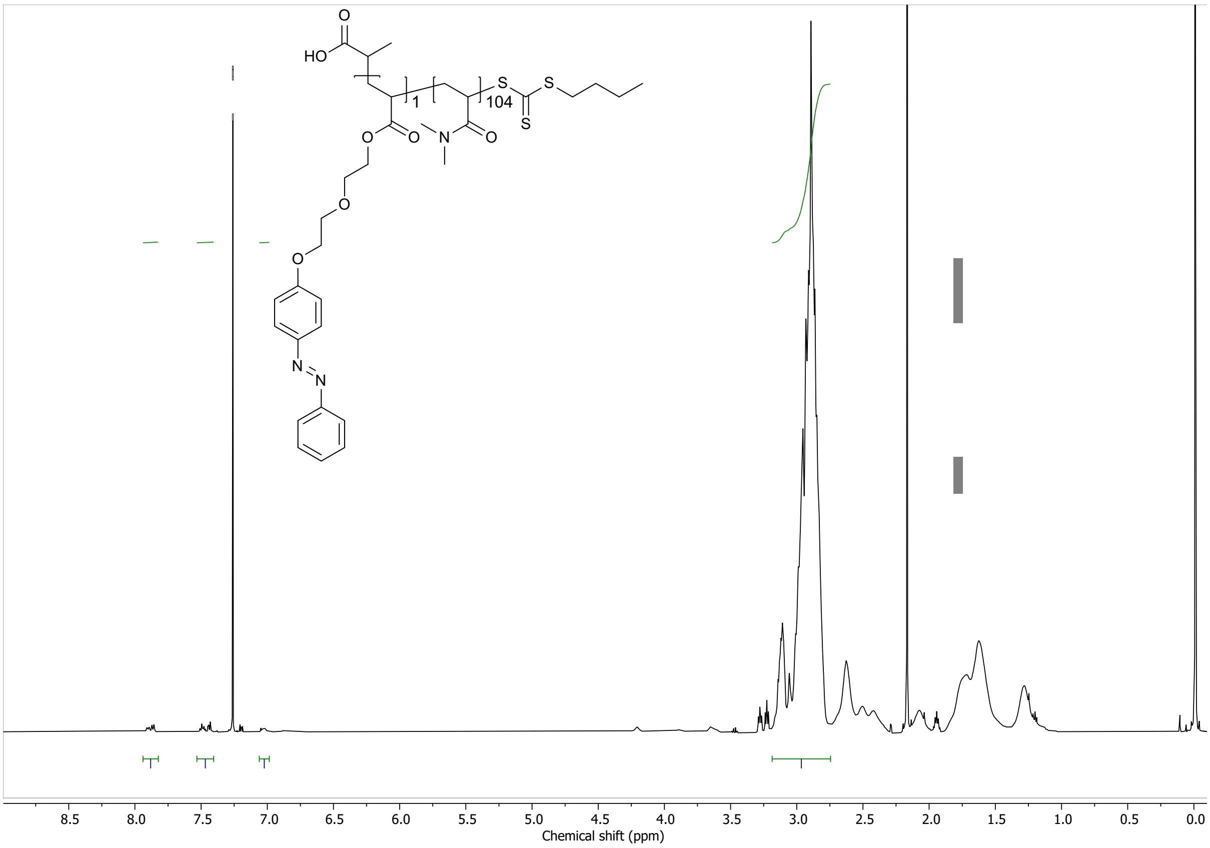


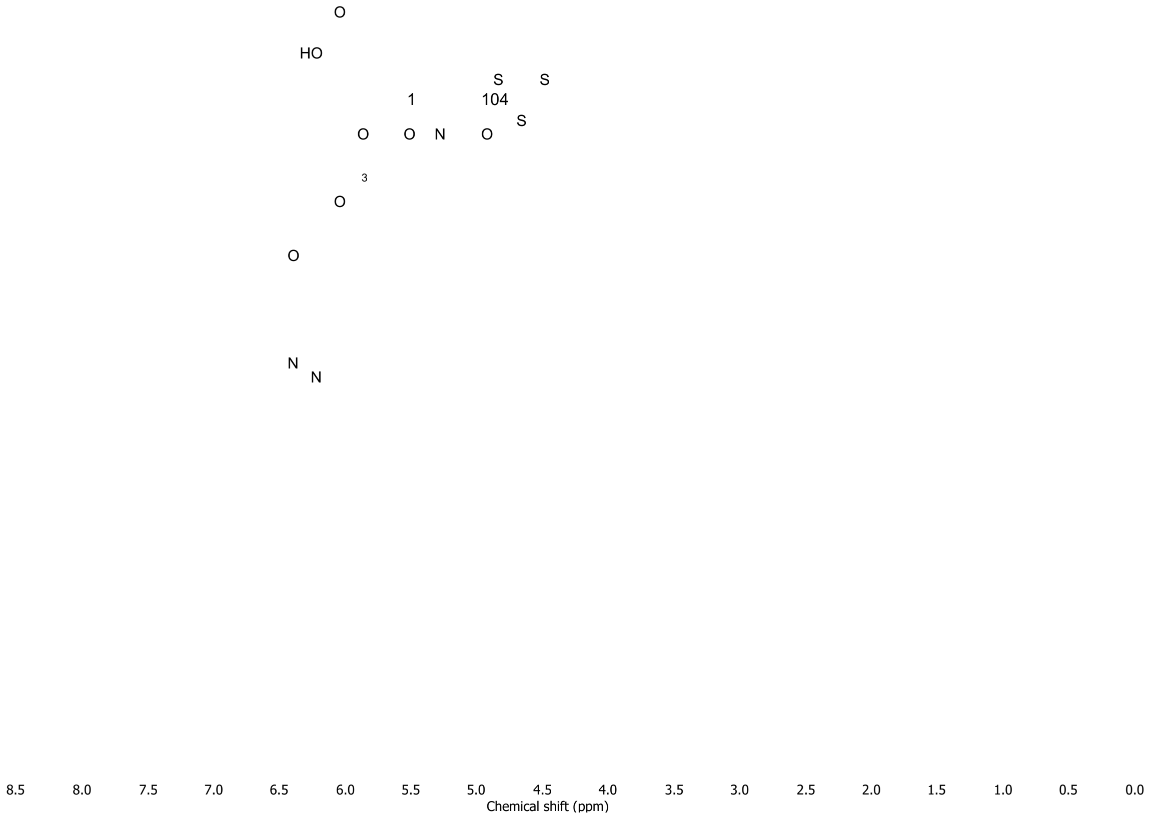


Figure S47: $^1$H NMR spectra of p(DMA$_{99}$-s-[DEG-Azo]$_1$) and p(DMA$_{99}$-s-[TEG-Azo]$_1$) in $CDCl_3$.

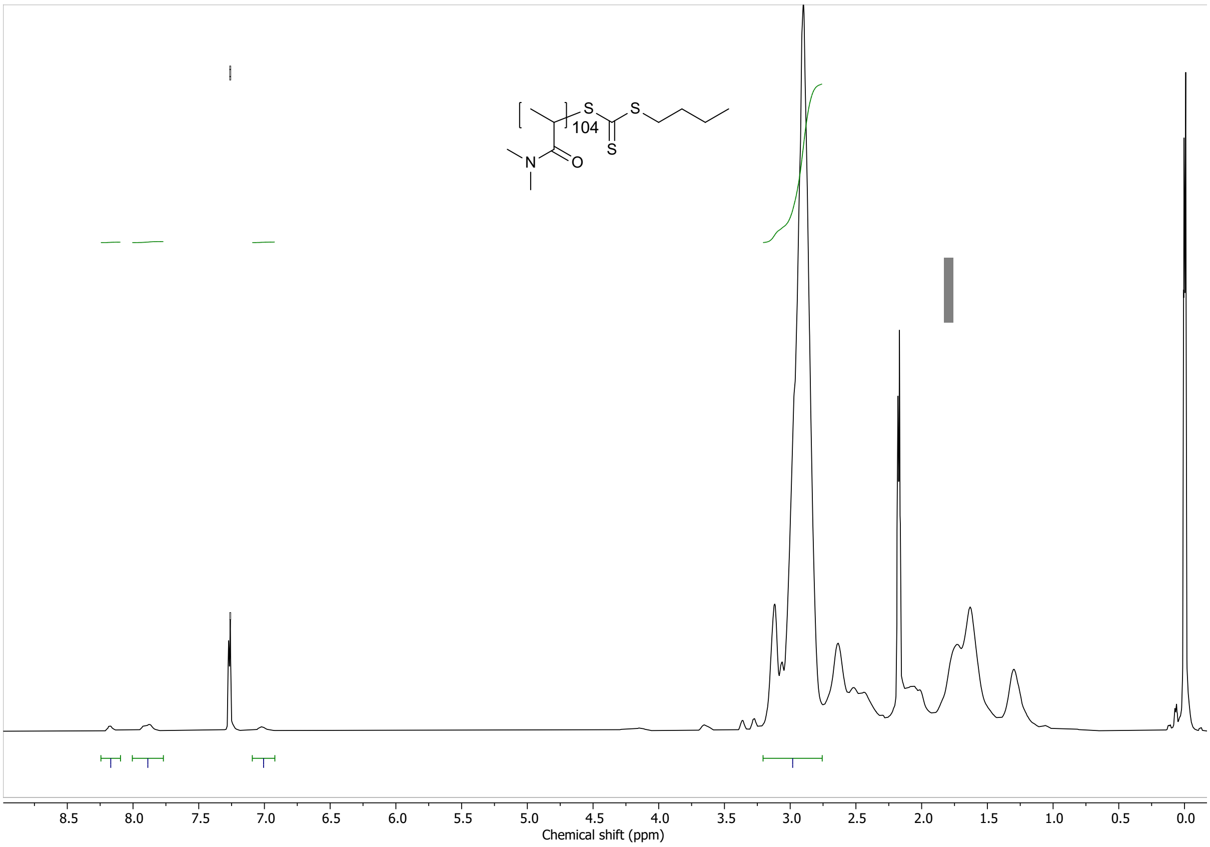


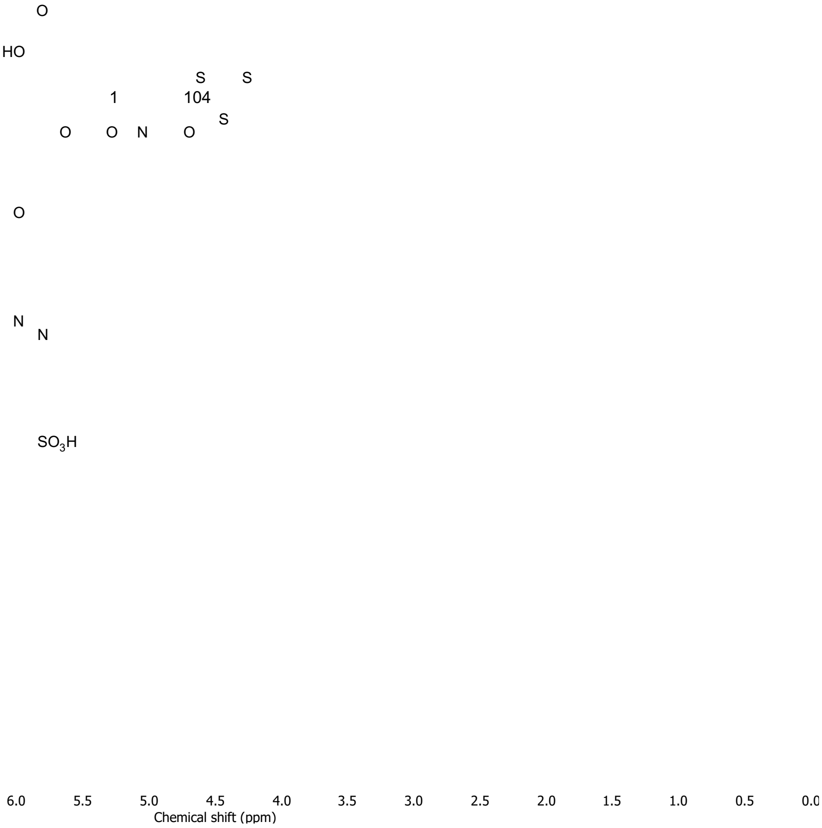


Figure S48: $^{1}H$ NMR spectra of p($DMA_{99}$-s-[Azo-COOH]$_1$) and p($DMA_{99}$-s-[Azo-$SO_3H$]$_1$) in $CDCl_3$.

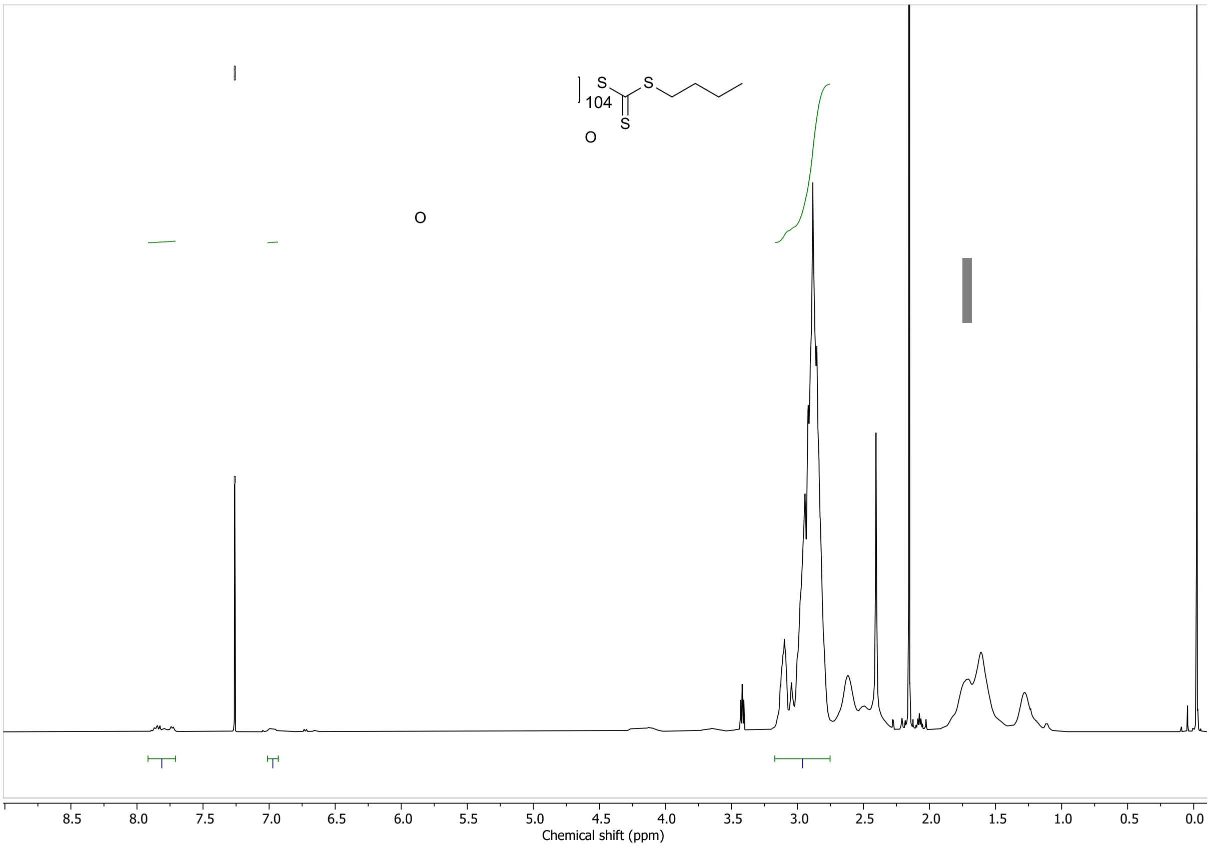


Figure S49: $^{1}H$ NMR spectrum of p($DMA_{99}$-s-[Azo-Amide]$_1$) in $CDCl_3$.

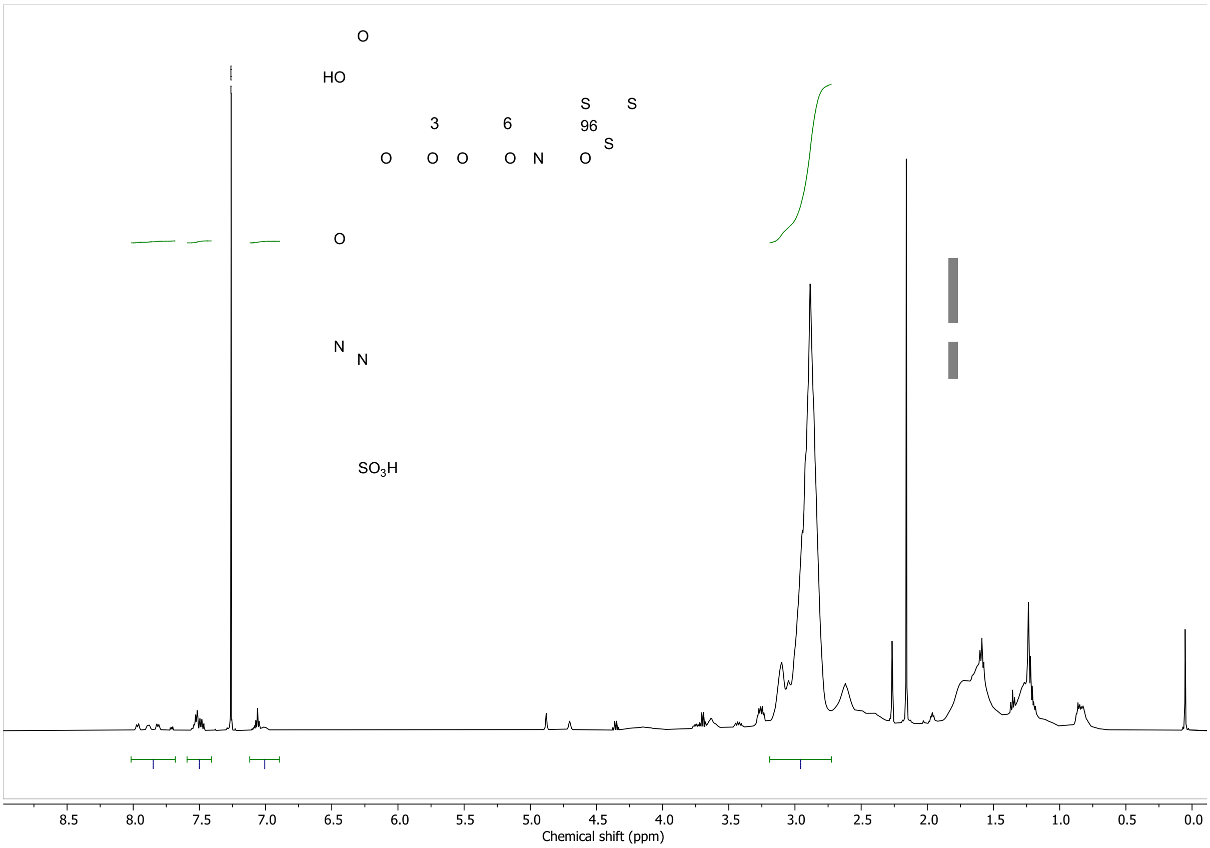


Figure S50: $^{1}H$ NMR spectrum of pp($DMA_{90}$-s-[Azo-$SO_3H$]$_3$) in $CDCl_3$.

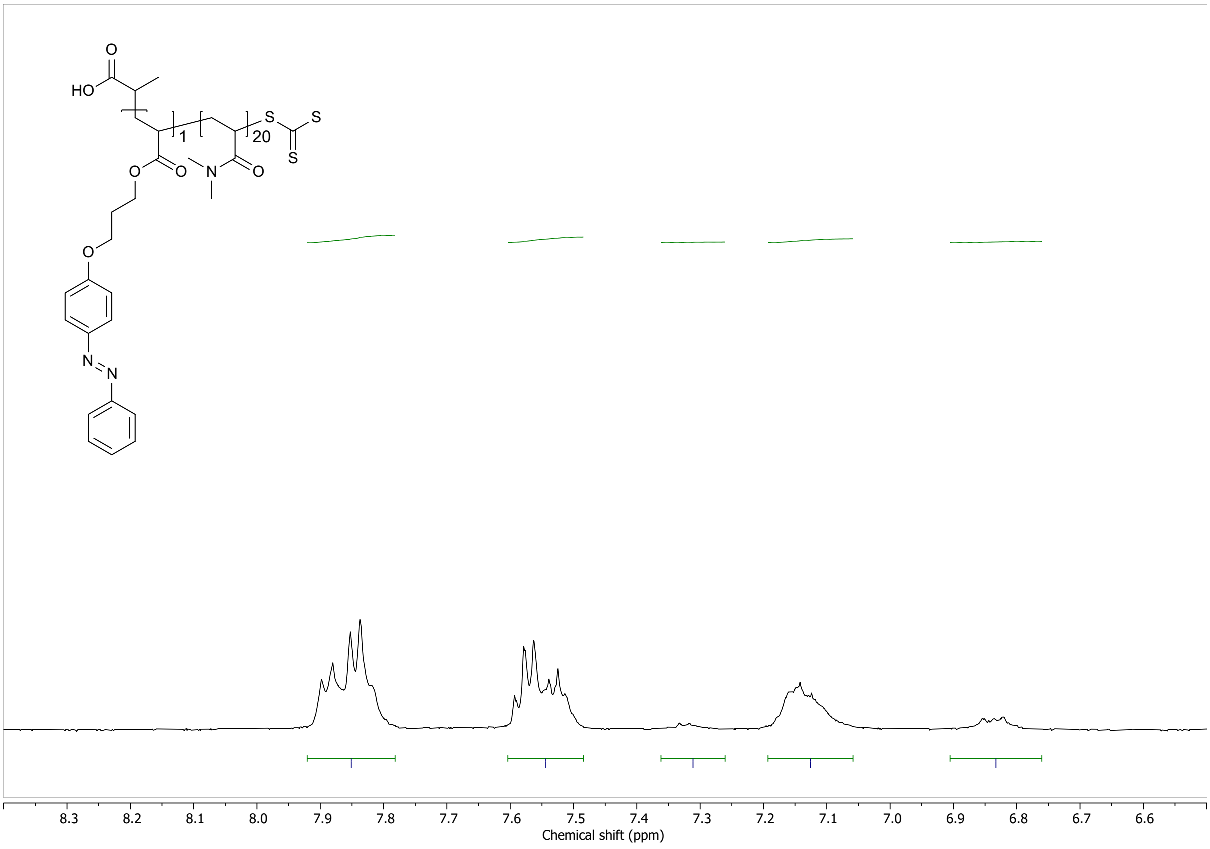


O

HO

S S

1 20

S

O O N O

O

N

N

8.3 8.2 8.1 8.0 7.9 7.8 7.7 7.6 7.5 7.4 7.3 7.2 7.1 7.0 6.9 6.8 6.7 6.6

Chemical shift (ppm)

Figure S51: $^{1}$H NMR spectra of p($DMA_{19}$-*s*-$Azo_1$) before and after irradiation with UV light (365 nm) in DMSO-$d_6$.

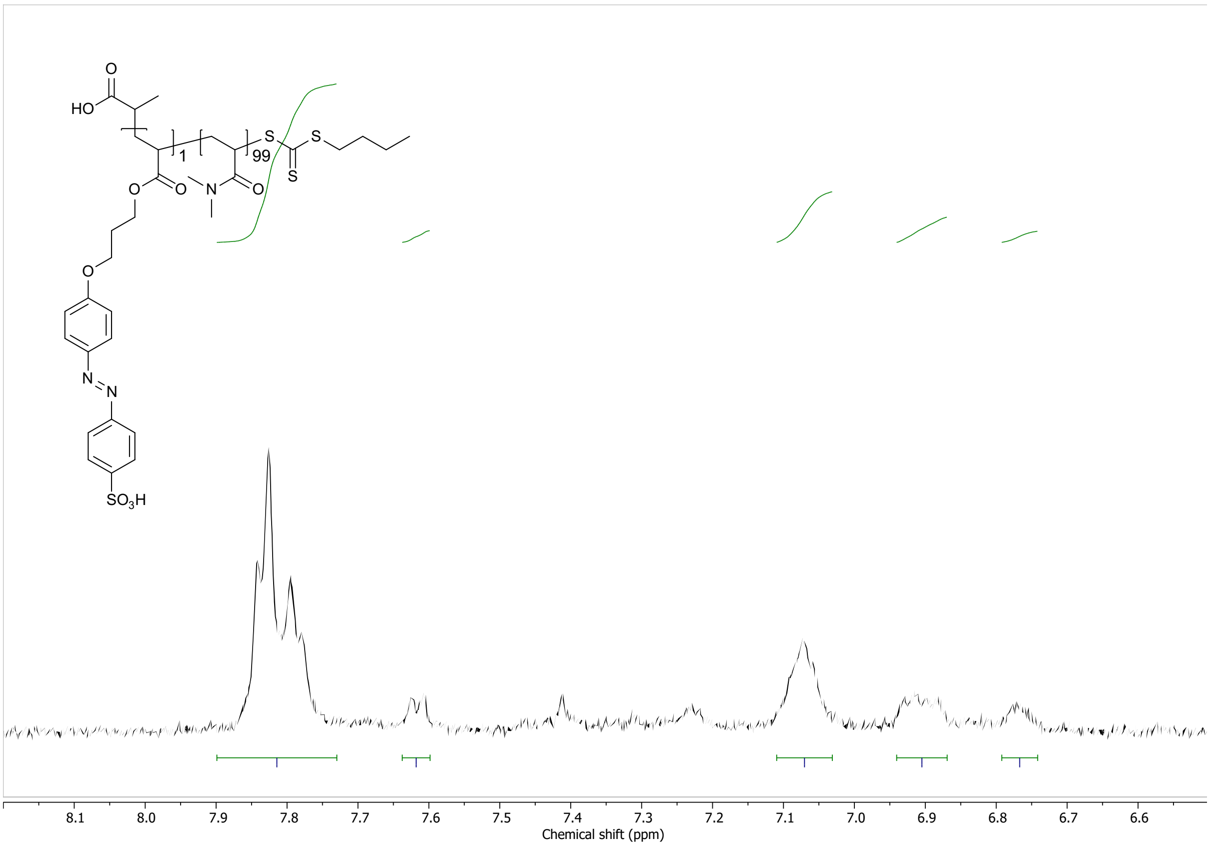


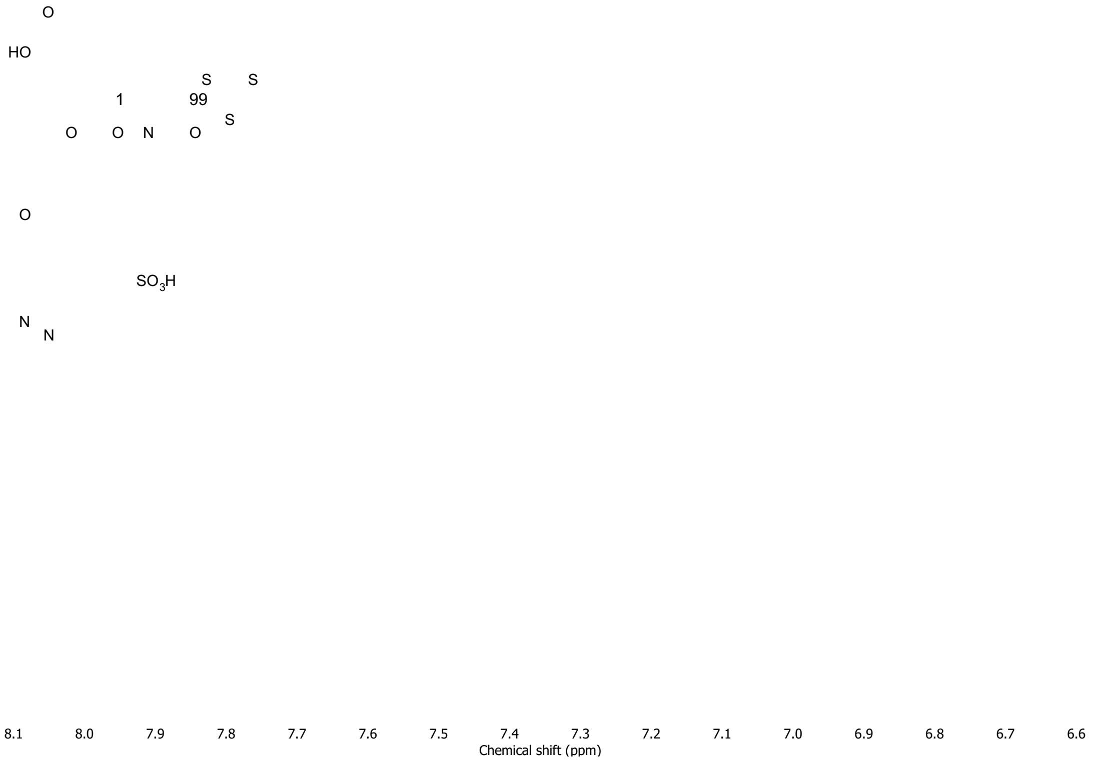


Figure S52: $^{1}H$ NMR spectra of p($DMA_{99}$-s-[Azo-$SO_3H$]$_1$) before and after irradiation with UV light (365 nm) in $D_2O$.

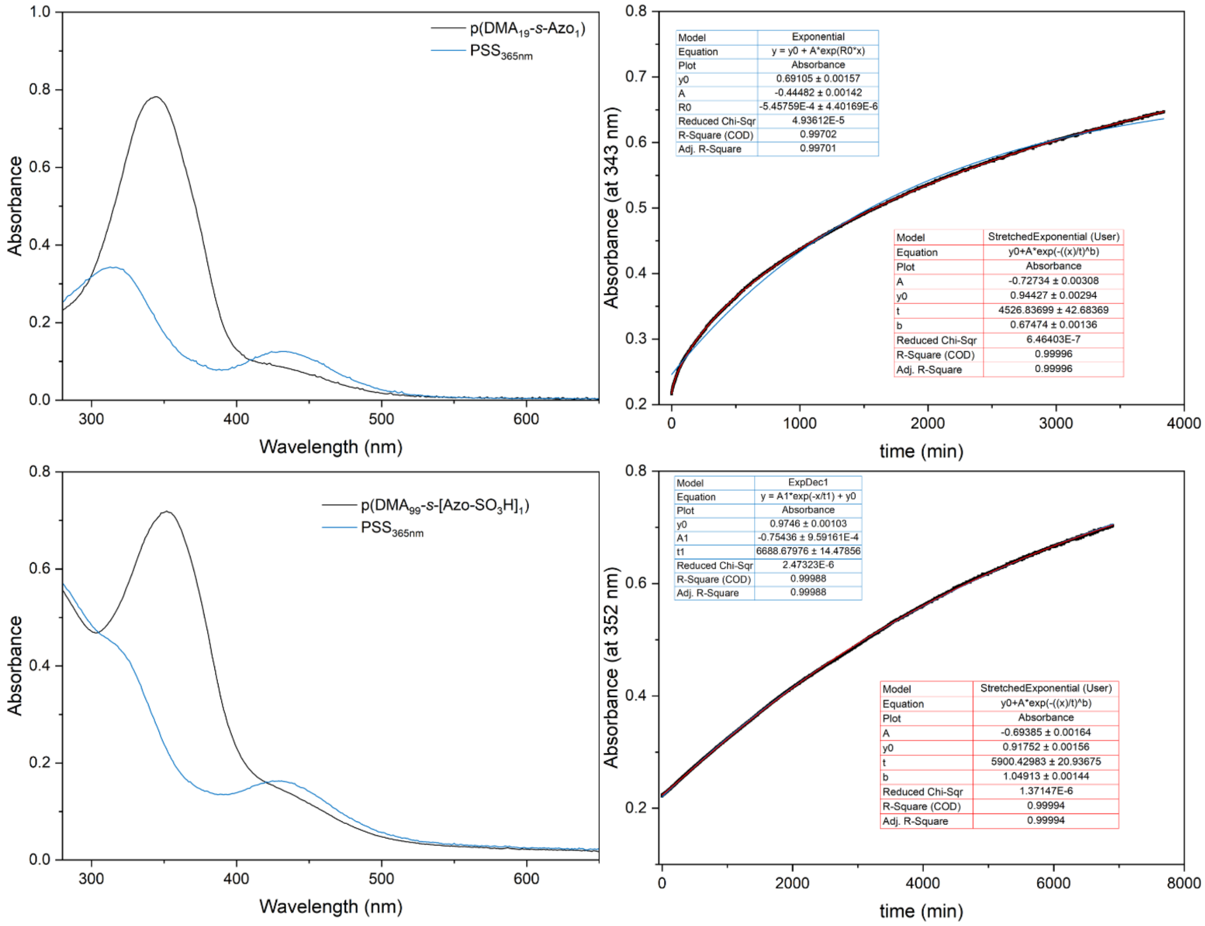


Figure S53: Half-life time studies of p($DMA_{19}$-*s*-$Azo_1$) and p($DMA_{99}$-*s*-[Azo-$SO_3H$]$_1$).
